# Diagnostic utility of clinical genome reanalysis in rare pediatric disorders using long-read sequencing

**DOI:** 10.1016/j.xhgg.2026.100620

**Published:** 2026-04-27

**Authors:** Elizabeth A. Werren, Purva Vats, Gabriel E. Rech, Michael Peracchio, Cameron King, Elizabeth J. Charnysh, Ryan D. Gorham, Peter A. Audano, Peter N. Robinson, Melissa A. Kelly, Adam P. Matson, Mark D. Adams, Louisa Kalsner

**Affiliations:** 1 The Jackson Laboratory for Genomic Medicine, Farmington, CT 06032, USA; 2 Division of Genetics, Connecticut Children’s, Hartford, CT 06106, USA; 3 Department of Research, Connecticut Children’s, Hartford, CT 06106, USA; 4 Department of Pediatrics, University of Connecticut School of Medicine, Farmington, CT 06030, USA; 5 Division of Neonatology, Connecticut Children’s, Hartford, CT 06106, USA; 6 Department of Immunology, UConn Health, Farmington, CT 06030, USA; 7 Division of Neurology, Connecticut Children’s, Hartford, CT 06106, USA

**Keywords:** long-read genome sequencing, rare disease, clinical variant interpretation

## Abstract

Over half of presumed genetic disease cases remain undiagnosed following short-read exome sequencing (SR-ES) or genome sequencing (SR-GS). Long-read GS (LR-GS) shows promise for uncovering etiologies missed by SR genetic testing, particularly structural variants (SVs). However, SV interpretation remains challenging due to limitations in call reliability, population allele frequency estimates, and functional impact prediction. To advance clinical LR-GS implementation, we analyzed the genomes of 19 children with suspected rare genetic conditions and prior negative or inconclusive clinical SR-GS/SR-ES as well as their parents using PacBio HiFi LR-GS. One additional family with limited DNA underwent Illumina SR-GS only, and 11 probands received SR-GS to complement small-variant detection. LR-GS data were processed using phased-assembly and read-based variant-calling pipelines validated in SV-positive control subjects, while SR-GS data were processed with the Illumina DRAGEN pipeline. Variants were prioritized using phenotype-driven approaches. Diagnostic variants (likely pathogenic or pathogenic) were identified in 2/20 (10%) families, while an additional 5/20 (25%) harbored findings of uncertain diagnostic significance, including variants of uncertain significance (VUSs) and variants in genes of uncertain significance (GUSs). All reported variants were detected independently of LR-GS by research SR-GS or by reanalysis of prior clinical SR data. Several LR-GS SV candidates were excluded after population allele frequency filtering, underscoring its importance in clinical SV interpretation. Overall, the observed 10% increase in diagnostic yield was achievable through SR analysis alone, as LR-GS was not required to identify diagnostic variants in this cohort. Functional studies are needed to clarify the clinical relevance of uncertain findings.

## Teaser

We benchmarked a long-read genome sequencing pipeline in children with suspected rare genetic disease and prior negative short-read testing. While all diagnostic variants were detectable through short-read data, our findings highlight the value of reanalysis and the need for improved interpretation frameworks for long-read data plus expanded population references.

## Main text

Developmental disorders exhibit high genetic heterogeneity, often with a constellation of non-specific features, posing major challenges for molecular diagnosis. While exome sequencing (ES) and genome sequencing (GS) have improved diagnostic yield, more than half of individuals with rare disease remain on diagnostic odysseys.^1^^,^^2^ The limitation of short-read GS (SR-GS) technologies in assessing the full repertoire of disease variation, such as complex structural variants (SVs) and altered methylation, may contribute to the low diagnostic yield of genetic testing.^2^^,^^3^ Long-read GS (LR-GS) is increasingly used to elucidate genetic etiologies of disease^4^^,^^5^^,^^6^^,^^7^^,^^8^; however, its clinical adoption remains limited, as few laboratories currently offer clinically validated LR diagnostic assays.^9^ Here, we applied LR-GS with state-of-the-art tools to a small, rare disease cohort where genetic diagnosis may have been missed due to the contributions of genes of uncertain significance (GUSs), difficult-to-detect SVs, non-coding variation, and/or epigenetic alterations to disease phenotypes. Our findings demonstrate that LR-GS has the potential to resolve some of these diagnostic gaps while underscoring the need for further computational and interpretative development to fully unlock its clinical utility.

As part of the effort to assess LR-GS utility in the diagnosis of rare developmental disorders, we recruited children and young adults with a suspected genetic condition and negative prior exome or SR-GS testing, together with their biological parents (Tables 1 and S1). All study participants provided informed consent in accordance with the ethical standards for human research subjects established by the institutional review board (IRB) committees at the Connecticut Children’s (CC) and The Jackson Laboratory for Genomic Medicine (JGM). See the supplemental material and methods for comprehensive details on participant recruitment, consenting, and protocols. Whole-blood samples were obtained from 19 trios, each comprising a biological mother (M), biological father (F), and affected child (proband [P]), as well as from one quad (M, F, and two affected siblings: P-1 and P-2). All affected participants presented with non-specific, syndromic conditions (Table S1; see the supplemental note). The most common phenotypes observed in the cohort include global developmental delay and/or intellectual disability (17/21, 81.0%), seizure (13/21, 61.9%), facial dysmorphisms (13/21, 61.9%), hypotonia (11/21, 52.4%), microcephaly (6/21, 28.6%), and failure to thrive (5/21, 23.8%) (Table S1; see the supplemental note).

**Table 1:** Summary of cohort demographics and genetic testing

**Proband demographics**	

Female, no. (%)	8/21 (38.1%)
Male, no. (%)	13/21 (61.9%)
Median age, years (IQR)	8 (10–4)
Consanguinity, no. (%)	1/21 (4.8%)

**Prior genetic testing**	**No. of probands (%)**

Short-read genome sequencing	2/21 (9.5%)
Exome sequencing	20/21 (95.2%)
Gene panel sequencing	13/21 (61.9%)
Chromosomal microarray	15/21 (71.4%)
Mitochondrial sequencing	4/21 (19.0%)
RNA sequencing	1/21 (4.8%)

**Present study testing**	**No. of families (%)**

PacBio HiFi long-read genome sequencing	
Trio	18/20 (95%)
Quad	1/20 (5%)
Proband only	0/20 (0%)
Illumina short-read genome sequencing	
Trio	1/20 (5%)
Quad	0/20 (0%)
Proband only	11/20 (55%)
RNA sequencing	
Trio	2/20 (10%)
Quad	0/20 (0%)
Proband only	0/20 (0%)

**Figure 1:**
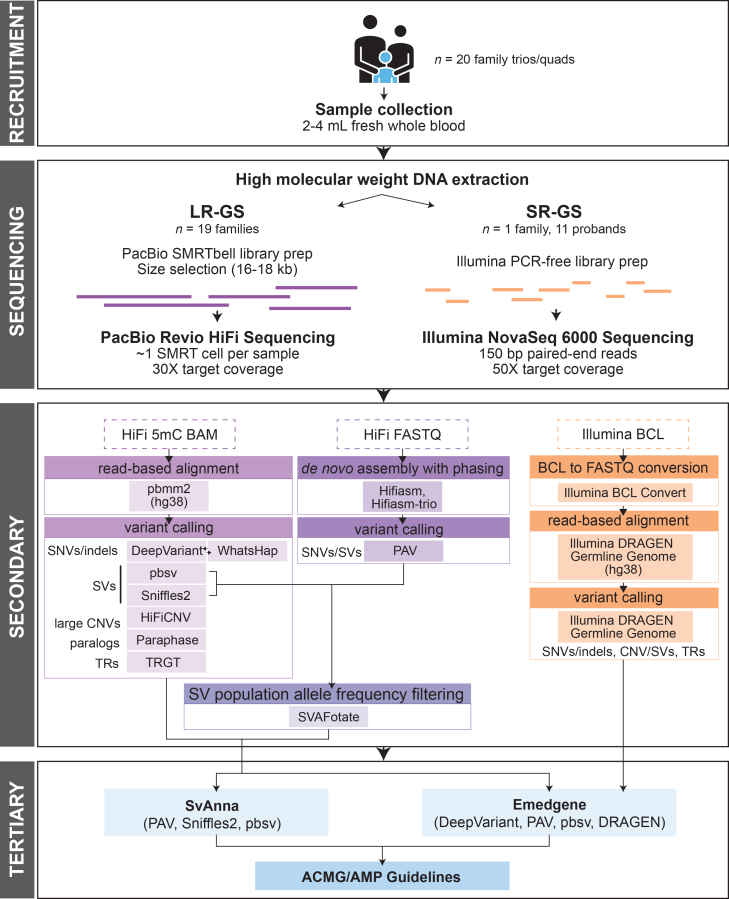
Diagram of study design Twenty families (19 trios and one quad) were enrolled in the study. Whole-blood samples were collected for high-molecular-weight DNA extraction. Samples with sufficient DNA yield underwent PacBio HiFi genome sequencing on a PacBio Revio system targeting 30× mean coverage (18 trios and one quad; 58 samples). A subset of long-read-sequenced families also received proband-only SR-GS (11 probands) on a NovaSeq 6000 targeting 50× mean coverage. One trio with insufficient DNA yield underwent Illumina SR-GS only. Secondary analysis for LR-GS incorporated assembly- and read-based approaches. SR-GS data were processed using the Illumina DRAGEN pipeline. All variants subsequently underwent tertiary analysis for classification and reporting.

PacBio HiFi LR-GS was performed on genomic DNA extracted from fresh whole blood using a Revio system on 19 families (18 trios and 1 quad); 11 of the probands’ samples also underwent concurrent Illumina SR-GS. One trio (2598) yielded limited DNA and underwent Illumina SR-GS only (supplemental material and methods). Samples were sequenced to an average mean coverage of 31.2× for LR-GS and 45.5× for SR-GS (Table S2). For LR-GS analysis, an in-house pipeline was developed to leverage both phased assembly-based^10^^,^^11^ and read-based^12^^,^^13^^,^^14^ tools to call a wide range of variant types against the hg38 no-alt reference genome,^10^ including single-nucleotide variants (SNVs), insertions/deletions under 50 bp (indels), SVs, and repeat expansions (Figure 1; supplemental material and methods). Benchmarking showed high performance across all callers (Figure S1; supplemental material and methods). SR-GS analysis was performed using the Illumina DRAGEN Germline Genome analysis pipeline and hg38 reference for variant calling of small variants (SNVs/indels), SVs, and repeat expansions (supplemental material and methods). All variants for LR-GS and SR-GS were prioritized using phenotype-driven filtering based on Human Phenotype Ontology (HPO) terms and interpreted according to the American College of Medical Genetics and Genomics (ACMG) and the Association for Molecular Pathology (AMP) guidelines.^15^ Tertiary analysis leveraged a combination of commercial and publicly available solutions to support structured evidence review and variant annotation, including the Illumina Emedgene software (LR-GS and SR-GS) and SvAnna (LR-GS only).^16^ Emedgene applies AI-driven phenotype-genotype matching algorithms to identify candidate variants from LR- and SR-called variants (SNVs, indels, repeat expansions, and SVs, excluding inversions), while SvAnna predicts pathogenicity for all LR-called SVs using an HPO-informed pathogenicity of structural variation (psv) score (supplemental material and methods).^16^ All monoallelic candidate variants affecting autosomal recessive genes were further evaluated for potential variants in *trans* (including both small variants and SVs) through both expert manual review and automated compound heterozygous detection in Emedgene.

**Figure 2:**
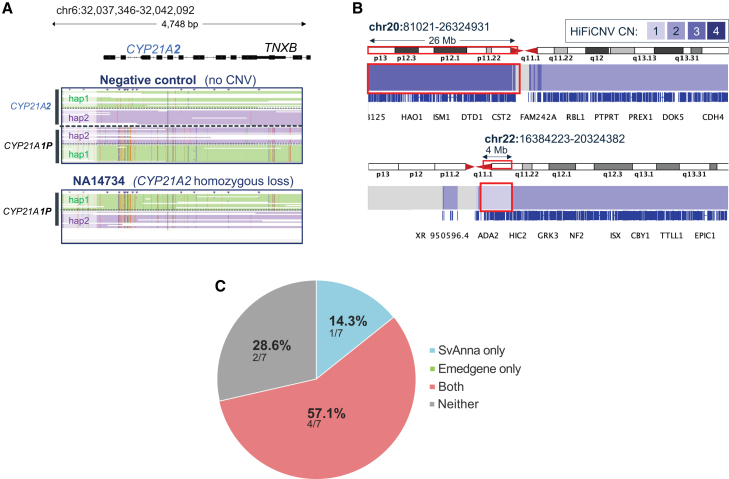
Evaluating LR-GS pipeline SV sensitivity (A) IGV of phased re-aligned BAMs from Paraphase at the *CYP21A2* locus in NA14734 compared to a control subject without CNVs affecting the locus. No reads with matching haplotypes to the *CYP21A2* paralog are observed, signifying homozygous loss. (B) IGV of HiFiCNV copy-number BEDGRAPHs, colored by copy number, capturing the translocation event involving the copy gain of the chr20 p arm and copy loss of a 4 Mb region on the chr22 q arm. (C) Pie chart summary of tertiary analysis approaches of positive SV controls. Of the 7 causal variants, 2 were not prioritized by either software (gray), 4 were prioritized by both (red), and 1 inversion was prioritized by SvAnna only (blue).

Given the growing recognition that SVs may represent a major source of disease heritability not captured by SR-GS, we first sought to ensure that our LR-GS analysis was robust for causal SV detection using previously characterized samples. To test the sensitivity of the pipeline for detecting and prioritizing known disease-causing SVs, we sequenced two positive disease control subjects with well-characterized pathogenic SVs (Coriell samples NA14734 and NA02944; Table S2). NA14734 is an individual with congenital adrenal hyperplasia due to 21-hydroxylase deficiency (MIM: 201910) with compound heterozygous deletion of the *CYP21A2* paralog, and NA02944 is an individual with DiGeorge syndrome (MIM: 188400) due to an unbalanced translocation (46,XY,+der(20)t(20;22)(q11.2;q11.2),−22.arr[hg38] 20p13p11.1(81021–26324931)x3,22q11.1q11.21(16384223–20324382)x1) (Figures 2A and 2B; Table S3). In addition, we obtained PacBio HiFi sequencing files from 5 families (PC01–PC05) harboring previously published causal SVs from the Genome Answers for Kids (GA4K) cohort,^4^^,^^8^ providing a broad repertoire of SV types for testing (Tables S2 and S3). For secondary and tertiary analyses, all causal variants were detected using assembly- and/or read-based variant callers and prioritized by SvAnna and/or Emedgene tools (Table S3). The compound heterozygous deletion involving the *CYP21A2* paralog in NA14734 was detected by the Phased Assembly Variant (PAV) and Paraphase callers and highly prioritized by SvAnna (psv = 83.6) and Emedgene (AI candidate and present in preset filters). The unbalanced translocation in NA02944 was captured only by HiFiCNV, the outputs of which are not compatible with SvAnna or Emedgene. The *KMT2E* c.729+113_1359−612del (GenBank: NM_182931.3) (p.Ala244Ter) variant segregating in family PC01, the *AARS2* 6p21.1(44306618–44310699)x1 deletion in family PC02, and the partial *NLRP12* deletion in family PC04 were detected by PAV, PacBio structural variant (pbsv), and Sniffles2 callers and ranked highly by both SvAnna and Emedgene. The *ACOX1* inversion in PC03 was detected only by PAV and Sniffles2, demonstrating variation in inversion sensitivity across SV callers. In addition, the inversion was ranked highly by SvAnna; inversion analysis is not currently supported by the Emedgene software. Lastly, the *STARD7* tandem repeat expansion segregating in family PC05 was detected fully in all family members with the PacBio Tandem Repeat Genotyping Tool (TRGT), as an insertion call with pbsv in two affected siblings (PC05P-1 and PC05P-2), and as an insertion call with Sniffles2 in one affected cousin (PC05C). The pbsv insertion call in individual PC05P-1 was present in one user-defined filter but was not labeled as a candidate by Emedgene, highlighting another area for tertiary analysis improvement. In summary, Emedgene and/or SvAnna were able to prioritize causal variants in 5/7 (71.4%) positive control subjects, with 2/7 (28.6%) requiring manual review of specialty caller outputs (Figure 2C).

**Figure 3:**
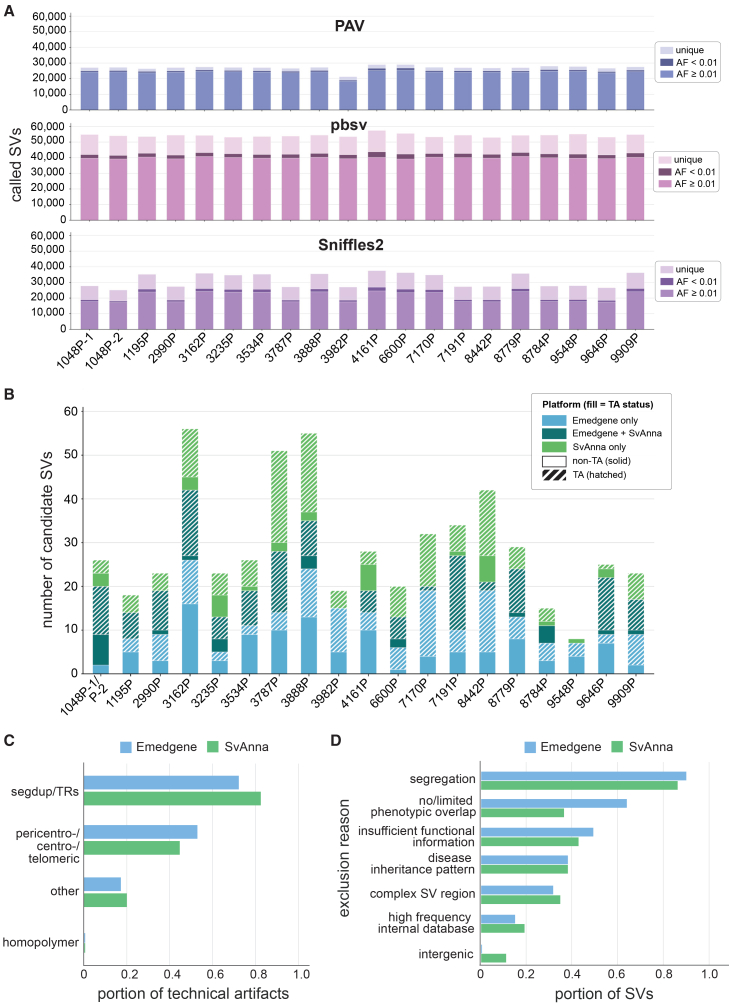
Structural variant filtering and prioritization in the LR-GS rare disease cohort (A) Plot showing the portion of called SVs per proband (*n* = 20) by LR SV caller before additional filtering with the following allele frequency (AF) in population databases: ≥0.01, <0.01, and 0. Frequencies were determined by SVAFotate^17^ against the following LR- and SR-GS population databases: HGSVC2/3, CoLoRSdb, GA4K, TOPMed, gnomAD, 1000 Genomes, and NHGRI-CCDG.^4^^,^^10^^,^^17^^,^^18^^,^^19^^,^^20^^,^^21^ (B) Plot showing the number of candidate SVs in Emedgene only (blue), SvAnna only (green, psv ≥ 2), or both platforms (teal) per proband (*n* = 20). Note: 1048P-1 and 1048P-2 were processed together in the same family and thus share the same candidates; only one bar is shown. Solid fill depicts true SVs, whereas hatched fill depicts the portion of SVs that were deemed technical artifacts. (C) Plot showing the portion of technical artifact calls by underlying reason(s) for all probands (*n* = 20). (D) Plot showing the portion of SVs across all probands (*n* = 20) that met different exclusion criteria. Emedgene is in blue, and SvAnna is in green.

Having established SV detection performance, we next evaluated the diagnostic yield of SVs in our LR-GS rare disease cohort. To optimize SV prioritization in the study cohort where causal variants are unknown, SVs with <80% reciprocal overlap with an allele frequency ≥0.01 in the following LR- and SR-GS population databases were first filtered out: Human Genome Structural Variation Consortium (HGSVC) v.2 and v.3, Consortium of Long Read Sequencing (CoLoRS), GA4K, Trans-Omics for Precision Medicine (TOPMed), Genome Aggregation Database (gnomAD), 1000 Genomes, and the National Human Genome Research Institute Centers for Common Disease Genomics (NHGRI-CCDG) program.^4^^,^^10^^,^^17^^,^^18^^,^^19^^,^^20^ Population filtering reduces the SV load for tertiary analysis by 66.2%–88.9%, from an average per individual of 27,076–54,261 SVs to 3,004–14,317 SVs, depending on the caller (Figure 3A). As the first filtering step in our workflow, the relatively higher SV counts for pbsv likely reflect more false positives, particularly in low-complexity repetitive regions, which are partially mitigated by Sniffles2’s coverage-adaptive, repeat-aware filtering and PAV’s assembly-based breakpoint resolution. Downstream tertiary quality filtering combined with manual review further refined the candidate SV set. For SvAnna, a prioritization threshold of psv ≥ 2 was applied, aligning the number of candidates more closely with those from Emedgene (Figure 3B; supplemental material and methods). A total of 386 and 321 candidate SVs were prioritized by Emedgene and SvAnna, respectively, across all 19 LR-GS cases, with an average of 20 Emedgene and 17 SvAnna SV candidates per individual (Figure 3B). Of these, a total of 159 candidates were prioritized by both tools, with an average of 8 candidates per individual (Figure 3B). Manual inspection of aligned sequence reads in the Integrative Genomics Viewer (IGV) revealed that a substantial number of candidate SVs were technical artifacts, either entirely absent or with insufficient, ambiguous evidence supporting the SV. A high proportion of SvAnna-only (124/162, 76.5%) and Emedgene-SvAnna-shared (135/159, 84.9%) candidate SVs were determined to be technical artifacts compared to roughly half of the Emedgene-only candidates (109/227, 48%) (Figure 3B). Technical artifact calls primarily arose from genomic regions containing segmental duplications, tandem repeats, and/or other repetitive loci, including homopolymer stretches, centromeres, pericentromeres, and telomeres (Figure 3C). For remaining candidates, SVs were most often excluded due to segregation of the variant in unaffected family members (87%–91%) and/or no or limited phenotypic overlap with the disease-associated gene(s) affected by the SV (37%–65%) (Figure 3D). In addition, several SVs affected loci with insufficient functional information (e.g., regulatory or intronic variants; 39%) or that fell within a genomic region populated with other SVs that are common, overlapping, or nearby in LR-GS population databases (32%–35%) (Figure 3D). A few SVs affected intergenic regions of unknown functional importance (1%–11%) (Figure 3D). Lastly, some SVs were common within the study cohort (15%–19%), underscoring the need for continual expansion of LR-GS population databases (Figure 3D).

Despite rigorous optimization and benchmarking of SV detection, diagnostic findings in our cohort were exclusively small variants (SNVs and indels) that were identifiable with SR-GS. Research use only (RUO) findings were shared with the medical team for 14/20 (70%) of families: 13/20 (65%) had small variant findings and 1/20 (5%) had SV findings (Table S4). Upon review of these candidate variants with the medical team, half were deemed inconclusive, resulting in a total of 7/20 (35%) families with potential diagnostic findings (Tables 2 and S4; Figure S2). The inconclusiveness of variants was attributed to factors such as mode of disease inheritance, uncertain population frequency data, limited phenotypic overlap, and insufficient molecular evidence. For instance, individuals 9909P, 3787P, and 7191P harbor a single inherited pathogenic or likely pathogenic variant for autosomal-recessive conditions with strong phenotypic match; however, systematic evaluation for additional variants in *trans*—including small variants and SVs—did not reveal a second pathogenic allele. Individual 4161P harbors a *de novo* frameshift variant in a GUS—*PTPDC1*—with several nearby or downstream predicted loss-of-function (LoF) variants observed in the general population (gnomAD v.4).^22^ A hemizygous variant in the 3′ UTR of *FGF13* was identified in 1195P; while absent from gnomAD v.4^22^ and phenotypically consistent with *FGF13*-related X-linked intellectual developmental disorder 110 (MIM: 301095), this variant lacks evidence of functional impact. Individual 2990P is homozygous for a ∼50 kb deletion within a segmental duplication on 8p23.1 spanning genes *ZNF705D*, *FAM66D*, *USP17L2*, and *USP17L7*, which lies adjacent to a region with no coverage in all analyzed individuals, most likely reflecting a rare or false duplication in the reference assembly (Figure S3; Table S4). While all genes within the deletion interval are GUSs, the *FAM66D* long non-coding RNA (lncRNA) is highly expressed in cortical neurons,^23^ and its dysregulation has been implicated in Dravet syndrome^24^—biology and clinical features consistent with 2990P (Table S1; see the supplemental note). However, this deletion is part of a larger ∼247 kb deletion that is present in the heterozygous state at an allele frequency of ∼4.6% (6/130 alleles) in the newest release of HGSVC (v.3),^20^ suggesting that it may be too common to cause disease. Lastly, compound heterozygous variants in a *VPS26C*, including a possible downstream regulatory variant and a missense variant, were prioritized for individual 8784P due to a recent report implicating bi-allelic *VPS26C* variants in two related individuals with a neurodevelopmental disorder (NDD)^25^ with phenotypic overlap to 8784P (Table S1; see the supplemental note); however, preliminary RNA sequencing (RNA-seq) findings from participant peripheral blood mononuclear cells (PBMCs) suggest that expression of *VPS26C* is unaffected (Figures S4 and S5A).

**Table 2:** Summary of clinically confirmed diagnostic variants in present study (all small variants)

Individual	Gene	Variant type	Variant	Classification (ACMG codes)	Inheritance, zygosity	OMIM	Detection method: Research study^a^	Detection method: Clinical test site^b^	Diagnostic significance
8442P	*PAK2*	missense	c.1273G>A (GenBank: NM_002577.4) (p.Asp425Asn) (GenBank: NP_002568.2)	likely pathogenic (PM2_Mod, PS2_Strong)	*de novo*, heterozygous	AD - Knobloch syndrome 2 (MIM: 618458)	LR-GS, SR-GS	ES reanalysis	DV
2598P	*DDX17*	nonsense	c.1077G>A (GenBank: NM_006386.5) (p.Trp359∗) (GenBank: NP_006377.2)	pathogenic (PVS1_VS, PS2_Strong, PM2_Mod)	*de novo*, heterozygous	AD - DDX17 neurodevelopmental disorder^26^	LR-GS, SR-GS	Sanger sequencing	DV
1048P-1/1048P-2	*DOCK4*	missense	c.2498T>A (GenBank: NM_014705.4) (p.Val833Asp) (GenBank: NP_055520.3)	VUS (PM2_Mod, PP1_Supp, PP3_Supp)	maternal, heterozygous	AD - DOCK4-associated neurodevelopmental delay and microcephaly^27^	LR-GS	ES reanalysis	VUDS
7170P	*SOCS1*	missense	c.340G>A (GenBank: NM_003745.2) (p.Ala114Thr) (GenBank: NP_003736.1)	VUS (PM2_Mod)	maternal, heterozygous	AD - autoinflammatory syndrome, familial, with or without immunodeficiency (MIM: 619375)	LR-GS, SR-GS	ES reanalysis	VUDS
3982P	*GEMIN5*	missense	c.863C>G (GenBank: NM_015465.5) (p.Thr288Arg) (GenBank: NP_056280.2)	VUS (PM2_Mod)	maternal, heterozygous	AR - neurodevelopmental disorder with cerebellar atrophy and motor dysfunction (MIM: 619333)	LR-GS	ES reanalysis	VUDS
		missense	c.1291G>A (GenBank: NM_015465.5) (p.Ala431Thr) (GenBank: NP_056280.2)	VUS (PM2_Mod)	paternal, heterozygous	AR - neurodevelopmental disorder with cerebellar atrophy and motor dysfunction (MIM: 619333)	LR-GS	ES reanalysis	VUDS
8779P	*TRAK1*	missense	c.757G>A (GenBank: NM_001042646.3) (p.Val253Met) (GenBank: NP_001036111.1)	VUS (PM2_Mod)	paternal, heterozygous	AR - developmental and epileptic encephalopathy 68 (MIM: 618201)	LR-GS, SR-GS	ES reanalysis; Sanger sequencing	VUDS
		intronic	c.2066+915G>C (GenBank: NM_001042646.3)	VUS (PM2_Mod)	maternal, heterozygous	AR - developmental and epileptic encephalopathy 68 (MIM: 618201)	LR-GS, SR-GS	Sanger sequencing	VUDS
9548P	*TIMM23*	missense	c.563C>A (GenBank: NM_006327.4) (p.Thr188Asn) (GenBank: NP_006318.1)	GUS - N/A	biparental, homozygous	N/A	LR-GS	ES reanalysis; Sanger sequencing	VUDS

AD, autosomal dominant; AR, autosomal recessive; VUS, variant of uncertain significance; GUS, gene of uncertain significance; Mod, moderate; VS, very strong; Supp, supporting; DV, diagnostic variant; VUDS, variant of uncertain diagnostic significance; N/A, not applicable.

a Method(s) that independently detected the variant in the present research study.

b Method performed during clinical confirmation by the original clinical laboratory for the proband.

For the remaining eight individuals from seven families with clinically relevant findings, all nine variants were clinically confirmed by the source testing laboratory using clinical SR data or Sanger sequencing, with results returned to the families (Tables 2 and S4). Of these eight individuals, research SR-GS was performed for four individuals and successfully detected all five variants (Table 2), demonstrating that LR-GS was not required for their detection. Reported variants were stratified into the following two diagnostic categories based on existing classification schemes^28^: diagnostic variant (DV) and variant of uncertain diagnostic significance (VUDS) (Table 2).

**Figure 4:**
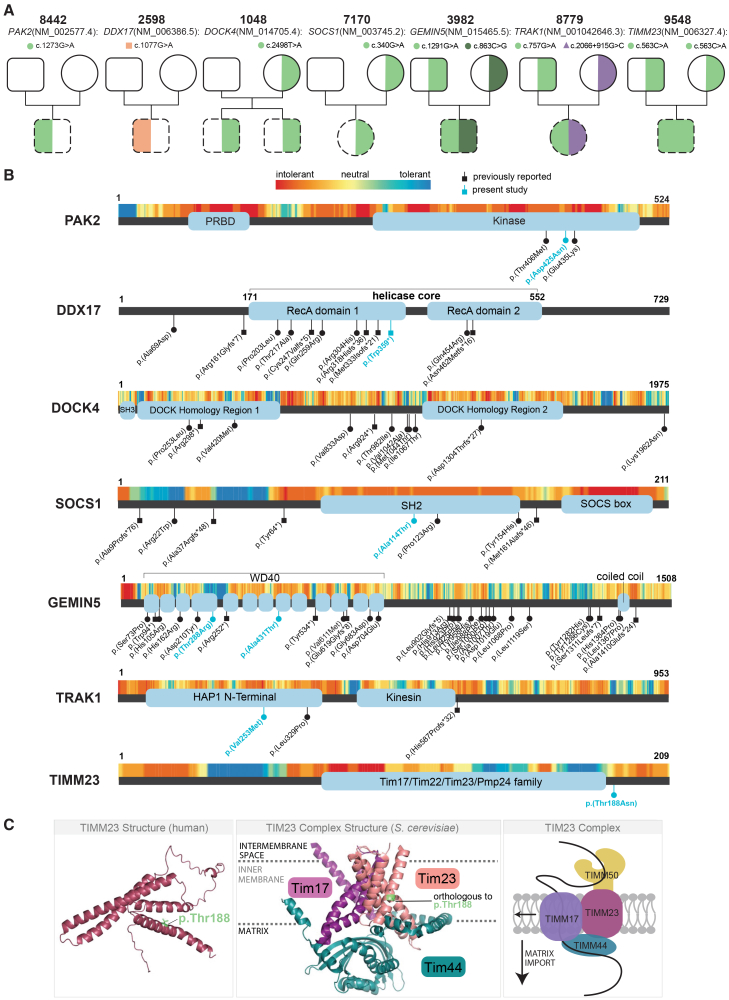
. Individuals with clinically confirmed diagnostic findings (A) Family pedigrees. Squares and circles represent assigned male and female at birth, respectively. Missense, nonsense, and potential regulatory variants are represented by green, orange, and purple shading, respectively. Heterozygous is indicated by half shading and homozygous by full shading. Affected individuals are represented with a dashed black outline. (B) Linear protein map of PAK2, DDX17, DOCK4, SOCS1, GEMIN5, TRAK1, and TIMM23 with annotated protein domains (UniProt.org) and rare disease-associated variants mapped. Variants detected in the present study are shown in blue; previously reported variants are in black. Above the protein map is a missense intolerance heatmap by residue (intolerant in red to tolerant in blue) based on dN/dS ratios (MetaDome^29^). (C) Human TIMM23 protein structure (AlphaFold^30^ AF-O14925 F1; PyMOL) with p.Thr188 residue mapped (left). Yeast TIM23 complex cryoelectron microscopy (cryo-EM) structure (PDB: 8E1M^31^; PyMOL) with the orthologous residue to p.Thr188 mapped (middle) and a TIM23 complex cartoon (right).

DVs were reported for two families. In family 8442, 8442P is heterozygous for a *de novo* likely pathogenic missense variant, c.1273G>A (GenBank: NM_002577.4) (p.Asp425Asn), in *PAK2* (Figures 4A and 4B). Pathogenic variants in *PAK2* cause Knobloch syndrome 2 (MIM: 618458), an autosomal-dominant disorder that strongly overlaps 8442P’s clinical phenotypes of global developmental delay, pyloric stenosis, and retinal detachment^32^ (Tables 2, S1, and S4; see the supplemental note). Following clinical confirmation and result sharing with the family, this molecular diagnosis prompted referral for reproductive genetic counseling, which informed family planning decisions. This result also provided diagnostic closure for the family, aiding understanding and acceptance of disease etiology and facilitating more focused care management.

In family 2598, who received SR-GS only, a pathogenic *de novo* variant was discovered in 2598P in *DDX17*, c.1077G>A (GenBank: NM_006386.5) (p.Trp359Ter) (Figures 4A, 4B, and S2A; Tables 2 and S4). *DDX17*, an RNA helicase, is highly intolerant to heterozygous predicted LoF variants in the general population (probability of LoF Intolerance [pLI] = 1, LoF observed/expected upper bound fraction [LOEUF] = 0.25; gnomAD v.4),^22^ suggestive of haploinsufficiency. Consistent with this, a recent report implicated *de novo* missense and predicted LoF variants in *DDX17* in individuals with NDD features, several of which overlap 2598P, including global developmental delay, speech difficulties, and hypotonia (Table S1; see the supplemental note).^26^ *DDX17* c.1077G>A (p.Trp359Ter) lies within the RecA domain 1 of DDX17, wherein the majority of reported variants cluster, and upstream of one reported frameshift variant (Figure 4B).^26^ While previously published *DDX17* disease variants are heterozygous, the variant allele fraction (VAF) of *DDX17* c.1077G>A in 2598P in our SR-GS data was 0.31, and clinical confirmation performed by the original clinical testing site was consistent with mosaicism. Taken together, additional clinical cases are needed to define the core phenotypic spectrum of this recently characterized disorder.

VUDSs were reported in five families. Similarly affected siblings 1048P-1 and 1048P-2 harbor a maternally inherited missense variant, c.2498T>A (GenBank: NM_014705.4) (p.Val833Asp), in *DOCK4* (Figures 4A, 4B, and S2B; Tables 2 and S4). Pathogenic variants in *DOCK4* have been implicated in autosomal-dominant *DOCK4-*associated neurodevelopmental delay and microcephaly with reports of incomplete penetrance.^27^ Although the disorder shows phenotypic overlap with each sibling’s clinical features (Table S1; see the supplemental note), c.2498T>A (GenBank: NM_014705.4) is a VUDS because it lacks supporting functional evidence. A maternally inherited missense variant was also detected in individual 7170P, c.340G>A (GenBank: NM_003745.2) (p.Ala114Thr), in *SOCS1* (Figures 4A, 4B, and S2C; Tables 2 and S4). *SOCS1* is associated with autosomal-dominant familial autoinflammatory syndrome with or without immunodeficiency (MIM: 619375), in which incomplete penetrance and variable expressivity are common, possibly underlying the phenotypic variability observed in affected maternal family members of 7170P (Figure 3D; Table S1; see the supplemental note).

Individual 3982P is compound heterozygous for two missense VUDSs that lie within tryptophan-aspartic acid (WD) repeats of *GEMIN5* and affect highly conserved residues: c.863C>G (GenBank: NM_015465.5) (p.Thr288Arg) and c.1291G>A (GenBank: NM_015465.5) (p.Ala431Thr) (Figures 4A, 4B, and S2D; Tables 2 and S4). GEMIN5 p.Ala431Thr affects a residue intolerant to missense variation (non-synonymous to synonymous substitution rate (dN/dS) = 0.29, MetaDome^29^; Figure 4B), and computational tools predict a deleterious effect of GEMIN5 p.Thr288Arg (Table S4). *GEMIN5* is implicated in autosomal-recessive NDD with cerebellar atrophy and motor dysfunction (NEDCAM; MIM: 619333), overlapping intellectual disability, seizures, hypotonia, absent speech, and motor delay in 3982P (Table S1; see the supplemental note). The majority of reported variants in *GEMIN5-*associated NEDCAM are missense (22/30, 73.3%), 6 of which also lie within the WD40 domain near GEMIN5 p.Ala431Thr and p.Thr288Arg (Figure 3B).^33^

Similarly, individual 8779P is compound heterozygous for VUDSs in *TRAK1*: c.757G>A (GenBank: NM_001042646.3) (p.Val253Met) and c.2066+915G>C (GenBank: NM_001042646.3) (Figures 4A, 4B, and S2E; Tables 2 and S4). *TRAK1* is associated with autosomal-recessive developmental and epileptic encephalopathy 68 (DEE68; MIM: 618201), which overlaps part of 8779P’s described phenotype: seizure, global developmental delay, intellectual disability, motor delay, absent speech, apnea, and hypotonia (Table S1; see the supplemental note). TRAK1 p.Val253Met lies within the HAP1 N-terminal domain of the protein, affecting a residue intolerant to missense variation (dN/dS = 0.26, MetaDome^29^) nearby a previously reported missense variant in DEE68^34^ (Figure 4B). The c.2066+915G>C (GenBank: NM_001042646.3) variant lies within intron 15 of 15 and affects a region with a proximal enhancer-like signature (ENCODE Candidate *Cis*-regulatory Elements database).^35^ This variant also results in a missense change in an alternative transcript of *TRAK1* (c.2079G>C [GenBank: NM_001349247.2] [p.Glu693Asp). *TRAK1* isoform GenBank: NM_001349247 (ENST00000327628.9) demonstrates low or absent expression in adult brain or developing cortex tissue, respectively, with expression observed in adult skin and heart tissue (GTEx v.8, ENCODE v.4, and PacBio Iso-Seq; Figure S6).^36^^,^^37^^,^^38^ Further molecular investigation of the functional consequence of this variant in both transcript contexts, as well as of TRAK1 p.Val253Met, is needed to substantiate their clinical significance in 8779P.

The final individual with a reported VUDS lies within a GUS. Individual 9548P is homozygous for *TIMM23* missense variant c.563C>A (GenBank: NM_006327.4) (p.Thr188Asn) (Figures 4 and S2F; Tables 2 and S4). While *TIMM23* has not been implicated in human disease to date, it demonstrates slight intolerance to missense variation gene wide (Z = 1.51, LOEUF = 0.84; gnomAD v.4^22^). The c.563C>A (GenBank: NM_006327.4) variant is present in 4/1,614,174 alleles (2.5 × 10^−6^) in the heterozygous state only in gnomAD v.4^22^ and affects a residue (p.Thr188) intolerant to missense variants (dN/dS = 0.59, MetaDome^29^) (Figure 4B; Table S4). Computational tools predict a deleterious effect of TIMM23 p.Thr188Asn on the protein (Table S4), with a deep learning prediction of a slightly destabilizing change in Gibbs free energy (ΔΔG) of −0.2 kcal/mol (DDMut^39^). TIMM23 is a key component of the translocase of the inner membrane (TIM23) complex, which mediates the import of preproteins to the inner mitochondrial membrane and mitochondrial matrix.^31^^,^^40^ While *TIMM23* has not been implicated in disease to date, pathogenic variants in other TIM23 complex subunits cause developmental disorders with partial phenotypic overlap to 9548P, including *TIMM50* in 3-methylglutaconic aciduria type IX (MIM: 617698), *DNAJC19* in 3-methylglutaconic aciduria type V (MIM: 610198), and *HSPA9* in EVEN-plus (epiphyseal, vertebral, ear, nose dysplasia plus associated findings) syndrome (MIM: 616854) (Table S1; see the supplemental note). Preliminary investigation of RNA expression (see supplemental material and methods) in PBMCs obtained from family 9548 does not suggest a change in expression of the TIM23 complex members (Figure S5B), nor of genes encoding mitochondria-associated proteins (data not shown). Immune signaling genes appear negatively enriched in 9548P PBMCs (Figures S5C–S5E), a finding consistent with previous transcriptomic evidence showing immune suppression via downregulation of adaptive immunity in PBMCs derived from individuals with mitochondrial disease^41^ as well as immunodeficiencies observed in individuals with mitochondrial disease.^42^ However, the lack of additional affected individuals to serve as biological replicates and the limited *TIMM23* expression in PBMCs warrant a more robust investigation to implicate *TIMM23* in human disease and evaluate the role of c.563C>A (GenBank: NM_006327.4) (p.Thr188Asn) in the clinical presentation of 9548P. Taken together, all described VUDSs in this study necessitate further functional investigation to determine their pathogenicity in the respective disorder.

In summary, diagnostic yield ranged from 10% for DV findings to 25% for VUDS findings. While these results are consistent with recent reports in larger LR-GS rare disease cohorts,^4^^,^^6^^,^^9^ the improved diagnostic yield in this small cohort can be attributed to genome reanalysis without increased detection from LR-GS. These results emphasize the need for periodic reanalysis, as new evidence can shift variant interpretation, exemplified by reclassifications of *PAK2* and *DDX17* variants in this study. In contrast, the reclassification of previously excluded variants as VUDSs in our study likely stems from inter-laboratory variability in clinical variant interpretation rather than emerging data. The absence of diagnostic SV findings, despite demonstrated analytic sensitivity, indicates that SVs were not a major contributor to disease in this small cohort. Several factors may have contributed to the lack of LR-specific findings in addition to low sample numbers, including ambiguity of SV interpretation. For instance, a proportion of candidate SVs that affected non-coding or intergenic regions were excluded in the present study given the lack of defined regulatory functions of affected regions. In addition, clinical interpretation strongly relies on allele frequency data from the general population to assess candidacy; however, LR SV population datasets are still incomplete compared to small-variant databases. Moreover, clinical SV databases such as ClinVar,^43^ ClinGen,^44^ and DECIPHER^45^ are sparsely curated. In some samples in the present study, SVs mapped to segmental duplication regions with reference bias and/or overlapped other similar or complex SVs in HGSVC^10^^,^^20^ data, requiring the use of arbitrary cutoffs to define SV identity for assessing allele frequency. In several samples, SVs that span multiple genes, including known disease-associated genes, were excluded from clinical consideration if segregation did not follow the expected inheritance pattern of the disease gene, reflecting a rigid interpretation based on monogenic Mendelian disease frameworks.

SVs may exert pathway-level, context-dependent, partial, or modifying effects, leading to incomplete penetrance and/or variable expressivity of disease—factors often underrecognized in clinical pipelines.^46^^,^^47^^,^^48^ Both common and rare SVs (including non-coding SVs lacking regulatory annotations) have been shown to affect the expression of multiple nearby genes, driving strong and/or pleiotropic effects on phenotypic diversity.^49^ Long-range regulation further complicates interpretation, as enhancers can act over large genomic distances, typically in *cis*^50^^,^^51^ but sometimes in *trans* via interchromosomal enhancer-gene interactions.^52^^,^^53^ Recurrent copy-number variant (CNV) syndromes often demonstrate incomplete penetrance,^54^^,^^55^ with recent analyses suggesting penetrance of just 1%–10%.^56^ Additionally, variable expressivity, dosage sensitivity, and modifier effects from other genomic variants contribute to clinical severity in recurrent CNV syndromes, notably for 1q21.1 deletions,^57^ 16p11.2 duplications,^58^ and Xq28 duplications.^59^ Collectively, these findings highlight the need for SV interpretation frameworks that move beyond single-gene, strong effect models to better incorporate regional effects, regulatory architecture, modifiers, and incomplete penetrance.^48^ In addition to genomic SVs, investigation into the contribution of non-coding variants and differential methylation to disease phenotypes in this cohort is currently ongoing, which may uncover missed molecular etiologies.

Nevertheless, evaluation of the LR analysis pipeline’s sensitivity using rare disease samples with known SVs highlighted the necessity of a multi-pronged approach due to the variable performance characteristics of read-based, assembly-based, and specialty SV callers across variant classes. Tertiary analysis demonstrated comparable performance between SvAnna and Emedgene for multiple SV types; however, it revealed a notable limitation of Emedgene in the annotation of inversion events. An abundance of small variants and SVs inherited from unaffected parents were prioritized by both SvAnna and Emedgene, highlighting the significance of trio-based analysis for effective candidate variant interpretation in rare disease. Likewise, the incorporation of population frequency filtering in LR-GS databases greatly refined SV prioritization by SvAnna and Emedgene, primarily by excluding common SVs not captured in SR datasets. Taken together, an integrative and systematic analysis is essential for maximizing the clinical value of LR-GS data. Although LR-GS did not provide additional diagnostic insight in this rare disease cohort, the findings underscore the importance of routine reanalysis to improve diagnostic yield.

## Data and code availability

All sequencing data used to support the findings of this study are restricted by the IRB committees at the CC and JGM to protect participant privacy. For participants who have consented to future data use, sequencing data have been deposited to the European Genome-phenome Archive (EGA). The accession number for the sequence data reported in this paper is EGA: EGAD50000002109 (Table S2). Variant classification data used to support the findings of this study have been deposited in the ClinVar repository for individual 8442C (ID 3081741). The LR-GS analysis pipeline has been converted to Nextflow and adapted for public release; the workflow is publicly available at https://github.com/TheJacksonLaboratory/jax-apml-lrs.

## Acknowledgments

We thank all families for their participation in this work. We thank Charles Lee, Juan C. Salazar, Christine R. Beck, Alyx Vogle, and Kunal Sanghavi for their intellectual contributions. We thank Tomi Pastinen and Emily Farrow at Children’s Mercy Hospital Kansas City for granting GA4K authorized data access. All sequencing was performed by Genome Technologies and the Advanced Precision Medicine Laboratory at the Jackson Laboratory for Genomic Medicine. This work was funded by Connecticut Children’s Research Institute and the 10.13039/100005946Jackson Laboratory.

## Author contributions

Manuscript and manuscript editing, E.A.W., M.A.K., G.E.R., M.D.A., A.P.M., L.K., and P.N.R.; figures, E.A.W.; participant recruitment and sample collection, A.P.M., L.K., M.P., and C.K.; sample extraction and sequencing, P.V. and R.D.G.; data analysis, E.A.W., M.A.K., G.E.R., and P.A.A.; IRB management, M.D.A., A.P.M., and E.J.C.

## Declaration of interests

The authors declare no competing interests.

## Web resources

AlphaMissense, https://alphamissense.hegelab.org/
ClinVar, https://www.ncbi.nlm.nih.gov/clinvar/
Combined Annotation Dependent Depletion (CADD), https://cadd.gs.washington.edu
dbGaP, https://dbgap.ncbi.nlm.nih.gov/home/
DDMut, https://biosig.lab.uq.edu.au/ddmut/
GenBank, https://www.ncbi.nlm.nih.gov/genbank/
MetaDome, stuart.radboudumc.nl/metadome
MetaScape, https://metascape.org/
Online Mendelian Inheritance in Man (OMIM), http://www.omim.org
PDB, https://www.rcsb.org
PolyPhen-2, http://genetics.bwh.harvard.edu/pph2/
SpliceAI, https://spliceailookup.broadinstitute.org/
UCSC Genome Browser, https://genome.ucsc.edu/
UniProt, https://www.uniprot.org/

## Supplemental information

Document S1. Figures S1–S6, supplemental note, and supplemental material and methods

Data S1. Tables S1–S4

Document S2. Article plus supplemental information
