# Supplementary material for: Diagnostic utility of clinical genome reanalysis in rare pediatric disorders using long-read sequencing
Source: HGG Adv. 2026 Apr 27;7(3):100620. doi: 10.1016/j.xhgg.2026.100620 (PMC13202554; doi:10.1016/j.xhgg.2026.100620)
Supplement: Document S1. Figures S1–S6, supplemental note, and supplemental material and methods [file mmc1.pdf]

HGGA, Volume 7

Supplemental information

Diagnostic utility of clinical genome reanalysis

in rare pediatric disorders using long-read sequencing

Elizabeth A. Werren, Purva Vats, Gabriel E. Rech, Michael Peracchio, Cameron King, Elizabeth J. Charnysh, Ryan D. Gorham, Peter A. Audano, Peter N. Robinson, Melissa A. Kelly, Adam P. Matson, Mark D. Adams, and Louisa Kalsner

TABLE OF CONTENTS

SUPPLEMENTAL NOTE: CLINICAL SUMMARIES 2

FIGURES 9

MATERIALS AND METHODS 18

REFERENCES 25

Supplemental Note: Clinical Summaries

8442P: Three-year-old male with hypotonia, global developmental delay, bilateral retinal detachment, and failure to thrive. He had feeding intolerance as a newborn and had pyloric stenosis repair and then gastrostomy tube placement and required jejunal feeding. He had a large patent ductus arteriosus which was closed surgically. He had abnormal eye movements and was diagnosed with bilateral retinal detachment. He can sit and scoot but does not walk. He uses a few words and signs. On exam he has microcephaly (Z= -2.8), with normal weight and height. He has simply shaped, posteriorly rotated ears and deep-set eyes. He has roving eye movements with intermittent nystagmus and irregular, non-reactive pupils. He has hypotonia and reduced reflexes. MRI brain showed prominent ventricles and sulci and abnormal signal within the globes of both eyes.

2598P: Four-year-old male with hypotonia, speech delay, hearing loss, and unsteady gait. Hypotonia was noted by one year of age and he walked late, after age two. He has mild to moderate high frequency sensorineural hearing loss bilaterally and wears hearing aids. On exam there are no dysmorphic features. He has scanning quality to his speech with articulation difficulty. He has hypotonia with mild ataxia and dysmetria. He had normal brain MRI.

1048P-1: Nine-year-old male with hypotonia, global developmental delay with intellectual disability, and history of epilepsy with infantile spasms. He had feeding difficulty at birth. He presented with infantile spasms at two months of age, successfully treated with ACTH. He is non-ambulatory and non-verbal. He has history of obstructive sleep apnea. Exam is notable for short stature (Z= -2), brachycephaly with relatively larger head circumference (Z=0) and a broad forehead. He has bilateral esotropia, diffuse hypotonia, and reduced reflexes. MRI of the brain was normal.

1048P-2: Four-year-old male with global developmental delay, hypotonia, and failure to thrive. He is non-ambulatory and non-verbal. He had gastrostomy tube placed due to failure to thrive but has continued poor weight gain. He has thoracic scoliosis. He had surgery to address esotropia of the right eye with amblyopia. Exam is notable for small size involving weight (Z= -3) and height (Z = -2.2) with relatively larger head size (Z=0). MRI spine identified a cauda equina arachnoid cyst. Brain MRI is normal.

7170P: Sixteen-year-old female with multiple medical issues including severe GI dysmotility, recurrent sinopulmonary infections, progressive dystonia of the right foot, postural orthostatic tachycardia syndrome (POTS), and joint hypermobility. She receives IVIG infusions for possible common variable immune deficiency (CVID). She had rectal prolapse and severe constipation leading to appendicocecostomy. Dystonia of the right foot began at 14 years of age and is treated with botulinum toxin injections. Physical exam is notable for dystonia of the right foot and brisk reflexes in both lower extremities. She had normal MRI of the brain and spine and normal nerve conduction study.

3982P: Eleven-year-old male with hypotonia, autism spectrum disorder, intellectual disability and epilepsy. He remains non-verbal and has prominent stereotypies. He drinks formula only and will not eat solid foods. Seizures began at age six and are controlled with two anti-epileptic medications. He has persistent hypotonia and tires quickly when ambulating. Exam is notable for triangular facial shape with widely spaced teeth, and long fingers and toes. EEG showed right sided focal spikes. MRI brain was normal.

8779P: Eight-year-old female with global developmental impairment and refractory epilepsy. She had feeding difficult with poor weight gain in infancy. She remains non-ambulatory and non-verbal.

Seizures began at age three years and have been refractory to treatment. She has daily seizures despite treatment with three anti-epileptic medications. She has amblyopia. On exam she has relative macrocephaly, large simply shaped ears, high arched palate, narrow nose and furrowed tongue. She has facial weakness and diffuse hypotonia. She had a normal brain MRI.

9548P: Nine-year-old male with global developmental delay with intellectual disability, movement disorder and epilepsy. He had failure to thrive in infancy leading to gastrostomy tube placement which continues to provide most of his nutrition. He developed chorea in early childhood and later dystonia managed with trihexyphenidyl and amantadine. He had two episodes of status epilepticus at age seven and is seizure free on levetiracetam. He is non-ambulatory and can speak in single words. He has normal growth parameters. On exam, he has no dysmorphic features. He has hypotonia, orofacial and limb dystonia, and choreiform movements. He has diffuse hypomyelination on brain MRI.

3162P: Four-year-old female with severe global developmental delay, feeding difficulty and mild hepatomegaly. She can sit briefly without support and can crawl for short distances. She is non-verbal. Diet is limited to formula and pureed foods. She has mildly enlarged liver on ultrasound with normal liver enzymes. Physical exam is notable for low weight and height ($Z < -2$). She has partially erupted teeth and prominent gums, full cheeks, heavy eyebrows, and up-turned nose. She has mildly distended abdomen with translucent skin and prominent veins over her chest. She has hypotonia and reduced muscle bulk in her legs with brisk reflexes. Brain MRI was notable for scattered non-specific signal change in the supratentorial white matter.

3534P: Seven-year-old male with refractory epilepsy, spastic quadriparesis and global developmental impairment. He was non-ambulatory and non-verbal. He had daily seizures despite treatment with three anti-epileptic medications. He was fed by gastrostomy tube due to

oropharyngeal dysphagia. Head imaging showed progressive cerebral volume loss. Family history was notable for parents being first cousins. He had a progressive decline in neurological function including autonomic dysfunction. He passed away in the setting of viral pneumonia with respiratory failure at the age of nine.

4161P: Six-year-old male with global developmental delay and autism spectrum disorder. He had gross motor delay, walking independently at 27 months of age. He used a few words but had mild regression after age three and is now nonverbal. He had a gastrostomy tube placed to augment nutrition as he takes only formula and purees by mouth. He has depressed nasal bridge, long eyelashes and prominent forehead as well as mild diffuse hypotonia. He had normal brain MRI.

6600P: Sixteen-year-old female with refractory epilepsy beginning at 6 months of age with infantile spasms and later evolution to Lennox-Gastaut syndrome. Her epilepsy is refractory to treatment with numerous medications, vagal nerve stimulator, and ketogenic diet and she continues to have daily seizures. She has severe global developmental impairment with loss of skills such as ability to crawl and ambulate with a walker. She is non-verbal. She has a gastrostomy tube for nutrition due to poor weight gain. She has intermittent hand wringing and unusual respiratory pattern with periodic hyperventilation followed by breath holding. Physical exam is notable for low weight and height (Z = < -3 SD). She has small ears, mild prognathism, and smooth philtrum. She has a broad chest and abdomen, narrow feet, and long toes. She has increased tone in her extremities with brisk reflexes. MRI is notable for cavum septum vergae and mild cerebellar vermian hypoplasia or volume loss.

3888P: Nine-year-old female with global developmental impairment and refractory epilepsy and clinical diagnosis of Aicardi syndrome. Seizures began on the third day of life. She had gastrostomy tube placed due to oropharyngeal dysphagia. She had refractory epileptic spasms,

not responding to treatment with steroids and multiple medications and has persistent myoclonic and tonic-clonic seizures. She can sit with support but cannot crawl or walk and is non-verbal. On exam, she has short stature (Z= -2) and microcephaly (Z= -2.4). She has left-sided coloboma and microphthalmia. She has up-turned nares and ears with over-folded superior helix. She has axial weakness with spastic diplegia of her lower extremities with brisk reflexes and contractures at the knees. Brain MRI shows bilateral peri-sylvian polymicrogyria and subependymal nodular heterotopia along the margins of the lateral ventricles. The posterior aspect of the corpus callosum is hypoplastic and dysmorphic and massa intermedia is enlarged.

3235P: Two-and-a-half-year-old male who had status epilepticus in the setting of a febrile illness at 22 months of age and was found to have leukodystrophy on brain imaging. He has mild developmental delay, walking at 17 months of age, with persistent unsteady gait. He has speech delay, using only a few words. He is non-dysmorphic on exam, though with borderline microcephaly (Z= -1.7) and mildly ataxic gait. Brain MRI revealed extensive confluent signal abnormality involving the cerebral and cerebellar white matter suggestive of a leukoencephalopathy. Immune and infectious work-up was negative.

1195P: Twenty-three-year-old male with autism spectrum disorder, intellectual disability, short stature, sensorineural hearing loss, and epilepsy. He has a history of hypospadias with penoscrotal transposition repaired in childhood. He has central hypothyroidism. Seizures began at 11 months of age, and he remains on medication for focal epilepsy. He is non-verbal and has progressive sensorineural hearing loss, now severe to profound bilaterally. He has aggressive behavior treated with aripiprazole. Physical exam is notable for short stature (Z= -2.5) and bifid uvula. Brain MRI is normal.

9909P: Two-year-old male with global developmental delay and dysmorphic features. He has dysphagia and does not eat solid foods. He can sit but cannot walk. He has pointy shaped ears with bow shaped upper lip, flattened nasal bridge, and small nose. He has hypotonia and stereotypic hand movements. Brain MRI was normal.

2990P: Eighteen-year-old female with autism spectrum disorder, intellectual disability and epilepsy. She was diagnosed with autism at age two. She uses a few single words. She has convulsive seizures treated with topiramate. EEG demonstrated focal and generalized spike wave discharges. MRI brain showed mild cerebellar tonsillar ectopia.

3787P: Newborn male with severe metabolic decompensation. Baby was born at term with birth weight of 7 lb 9 oz. Apnea was noted shortly after delivery, and he was intubated and required chest compressions. He had severe metabolic acidosis with pH of 6.63. Ammonia was elevated to 507, and lactic acid rose to > 20 raising concern for an inborn error of metabolism. He had bleeding with disseminated intravascular coagulation (DIC). He developed abnormal posturing and therapeutic cooling was initiated. He had profound hypotonia and developed fixed, dilated pupils. He expired at 24 hours of life after withdrawal of care.

7191P: One-year-old female born prematurely at 26.5 weeks with agenesis of corpus callosum, dysmorphic facial features and persistent oropharyngeal dysphagia requiring gastrostomy tube. She has had slow weight gain, shorter stature and microcephaly (Z= -2.2). She remains mostly tube fed. She has short palpebral fissures, and small nose and mouth with flattened appearance to facial features. She has trichiasis of the lower eyelids and nasolacrimal duct obstruction. MRI revealed agenesis of the corpus callosum and small optic nerves and chiasm.

9646P: Eight-year-old female with muscle weakness and autism spectrum disorder. She walked at 16 months of age but has persistent weakness with positive Gower maneuver. She has mild scoliosis. She was diagnosed with megalocornea without glaucoma. She has macrocephaly (Z= 2.2) and partial 2/3 syndactyly of the toes. She has reduced muscle bulk in her legs with weakness and trace to absent lower extremity reflexes. She had normal CPK and normal EMG/nerve conduction study. She had normal MRI of brain and lumbar spine.

8784P: Twenty-one-year-old male with intellectual disability, autism, and epilepsy. He had developmental delay noted in the first months of life. He was able to sit at four years and began taking steps at 10 years. He is non-verbal. He has contractures at the knees but can take a few steps independently. He has thoracic scoliosis. Tonic seizures began at 19 years of age and are controlled with lamotrigine. He did not lose his deciduous teeth. On exam, he has microcephaly (Z = -4.5), short stature (Z= -2.7) and low weight (Z= -2). He has prognathism and prominent columella, prominent interphalangeal joints and small feet with hammer toes. He has pectus excavatum and reduced muscle bulk, and walks with crouched gait. Brain MRI was normal.

Figures

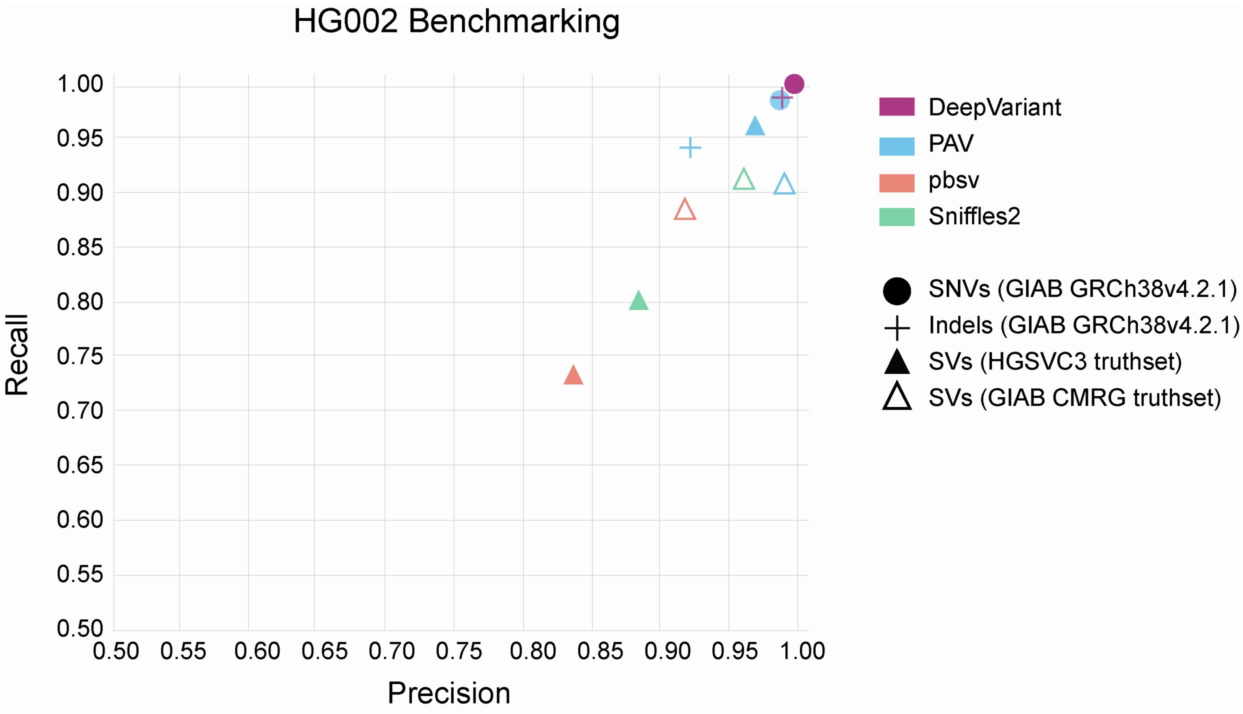

Figure S1. Benchmarking variant calling in LR-GS pipeline. Concordance plot of precision vs. recall values across variant callers (DeepVariant (dark blue), PAV (light blue), pbsv (purple), Sniffles2 (green)) and variant types (SNVs (·), indels (+), SVs (▲)) against the benchmarks: HG002 GIAB GRCh38 v4.2.1 (SNVs/indels), HGSVC3 (SVs, ▲), and GIAB challenging medically relevant genes (CMRG, SVs, △). For small variants, the highest precision (p) and recall (r) was obtained for DeepVariant SNV calls (p=99.9%, r=99.9%), followed by DeepVariant indels (p= 98.9%, r=98.6%), PAV SNVs (p= 98.7%, r=98.3%), and lastly PAV indels (p=92.3%, r=94.0%). For SVs, the highest concordance was observed for PAV (p=96.9%, r=96.1%), followed by Sniffles2 (p=88.4%, r=80.1%), and lastly pbsv (p=83.7%, r=73.3%). We further compared with the GIAB challenging medically relevant gene calls and find the highest precision for PAV (p=99.0%, r=90.7%), highest recall for Sniffles2 (p=96.1%, r=91.2%), and the lowest prevision and recall for pbsv (p=91.8%, r=88.4%).

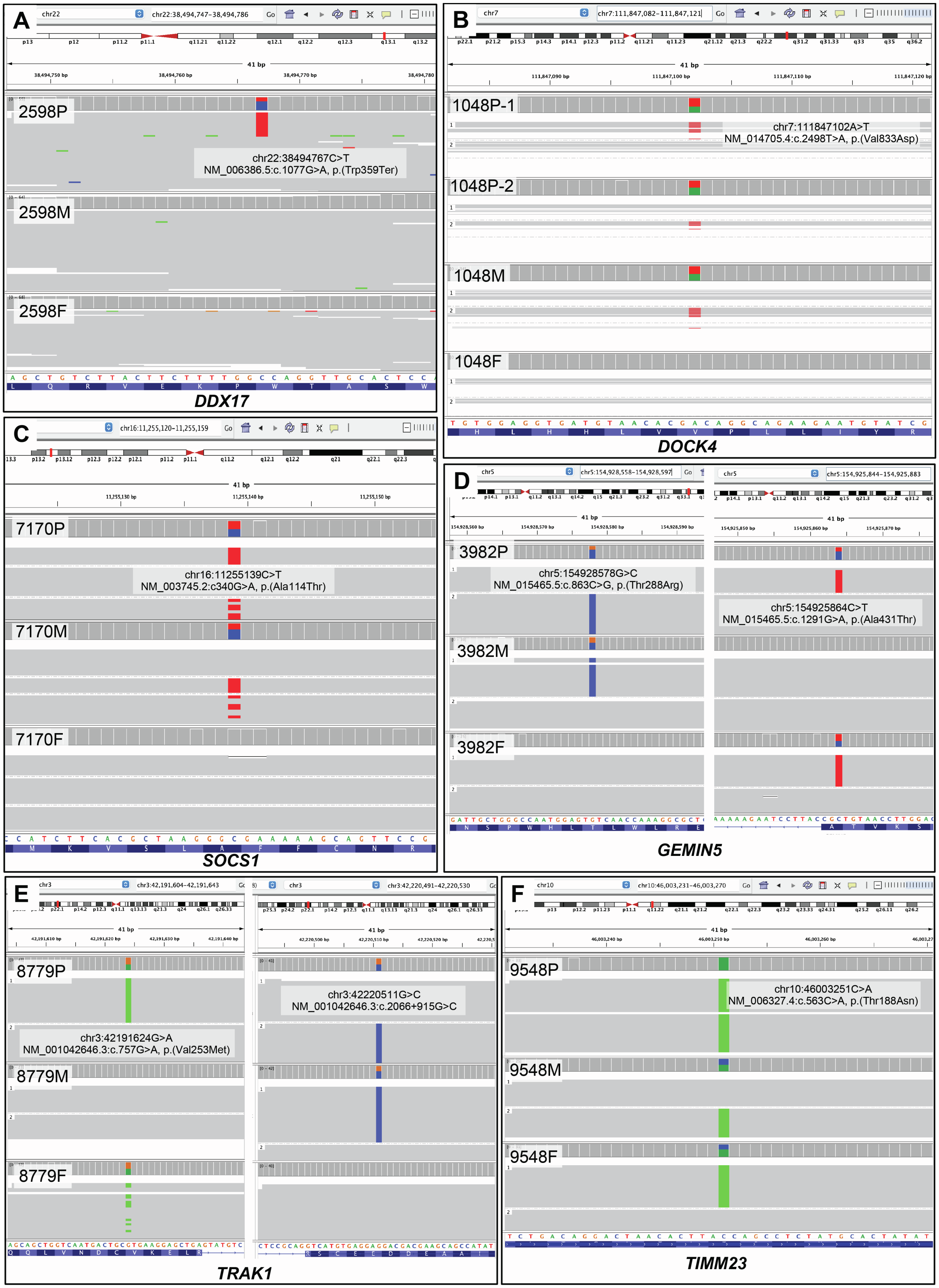
A
2598P
chr22:38494767C>T
NM_006386.5:c.1077G>A, p.(Trp359Ter)
2598M
2598F
DDX17
B
1048P-1
chr7:111847102A>T
NM_014705.4:c.2498T>A, p.(Val833Asp)
1048P-2
1048M
1048F
DOCK4
C
7170P
chr16:11255139C>T
NM_003745.2:c340G>A, p.(Ala114Thr)
7170M
7170F
SOCS1
D
3982P
chr5:154928578G>C
NM_015465.5:c.863C>G, p.(Thr288Arg)
chr5:154925864C>T
NM_015465.5:c.1291G>A, p.(Ala431Thr)
3982M
3982F
GEMIN5
E
8779P
chr3:42191624G>A
NM_001042646.3:c.757G>A, p.(Val253Met)
chr3:42220511G>C
NM_001042646.3:c.2066+915G>C
8779M
8779F
TRAK1
F
9548P
chr10:46003251C>A
NM_006327.4:c.563C>A, p.(Thr188Asn)
9548M
9548F
TIMM23

Figure S2. IGV assessment of clinically confirmed variants in Illumina SR bams aligned to hg38 no-alt reference for family 2598 (A) and in PacBio HiFi whatshap-phased bams aligned to hg38 no-alt reference, grouped by phase for families 1048 (B), 7170 (C), 3982 (D), 8779 (E), 9548 (F).

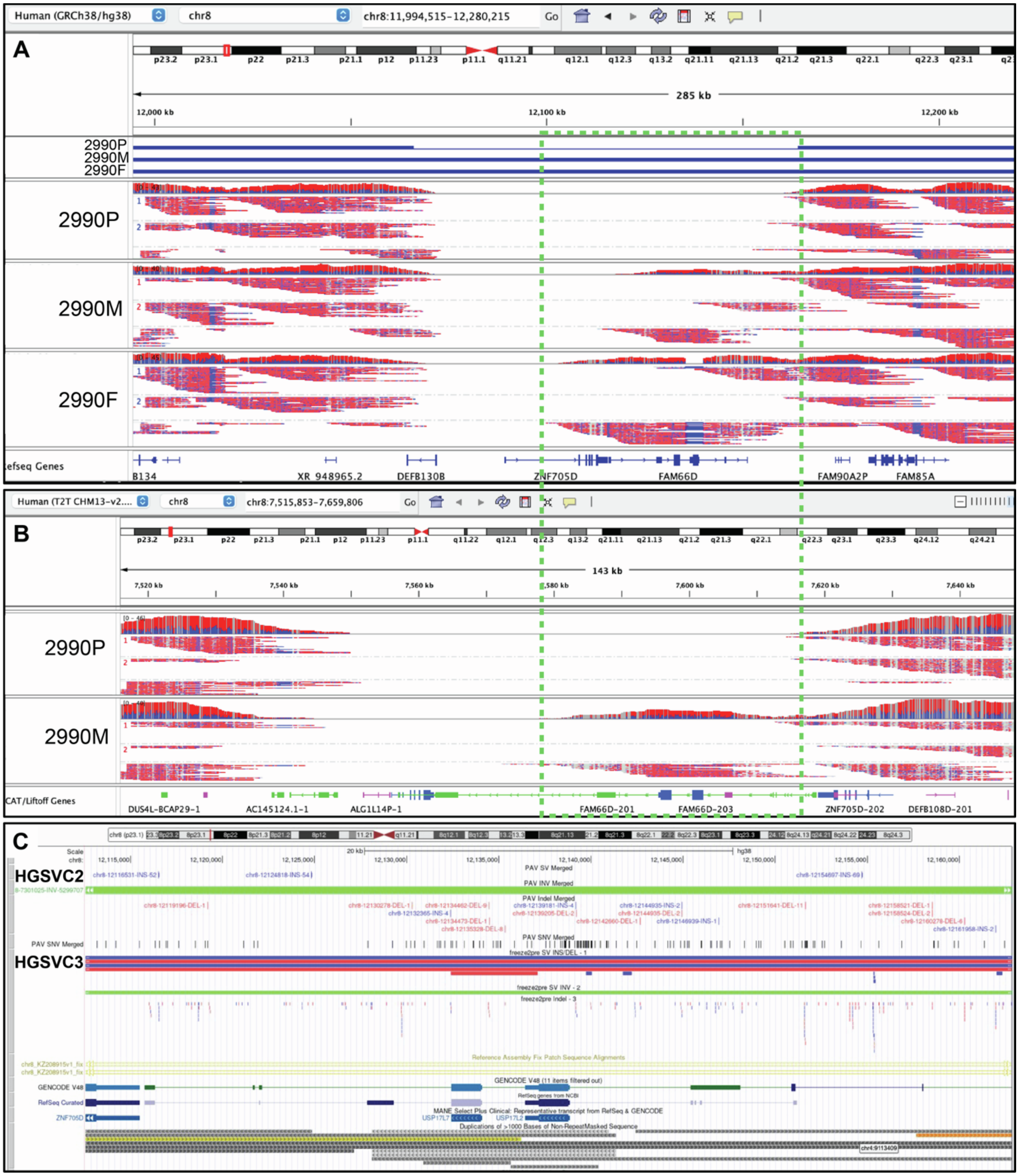

Figure S3. 8p23.1 deletion in family 2990 in (A) IGV of whatshap-phased bams aligned to reference hg38, with HiFiCNV copy number bedgraphs (top, blue), and (B) IGV of 2990P and 2990M whatshap-phased bams aligned to reference hs1 (T2T CHM13-v2.0). Tracks colored by 5mC tag and grouped by phase. Green box shows homozygous deletion region in 2990P. C) UCSC Genome Browser with HGSVC2 and HGSVC3 track hubs displayed, showing SVs in general population within region.

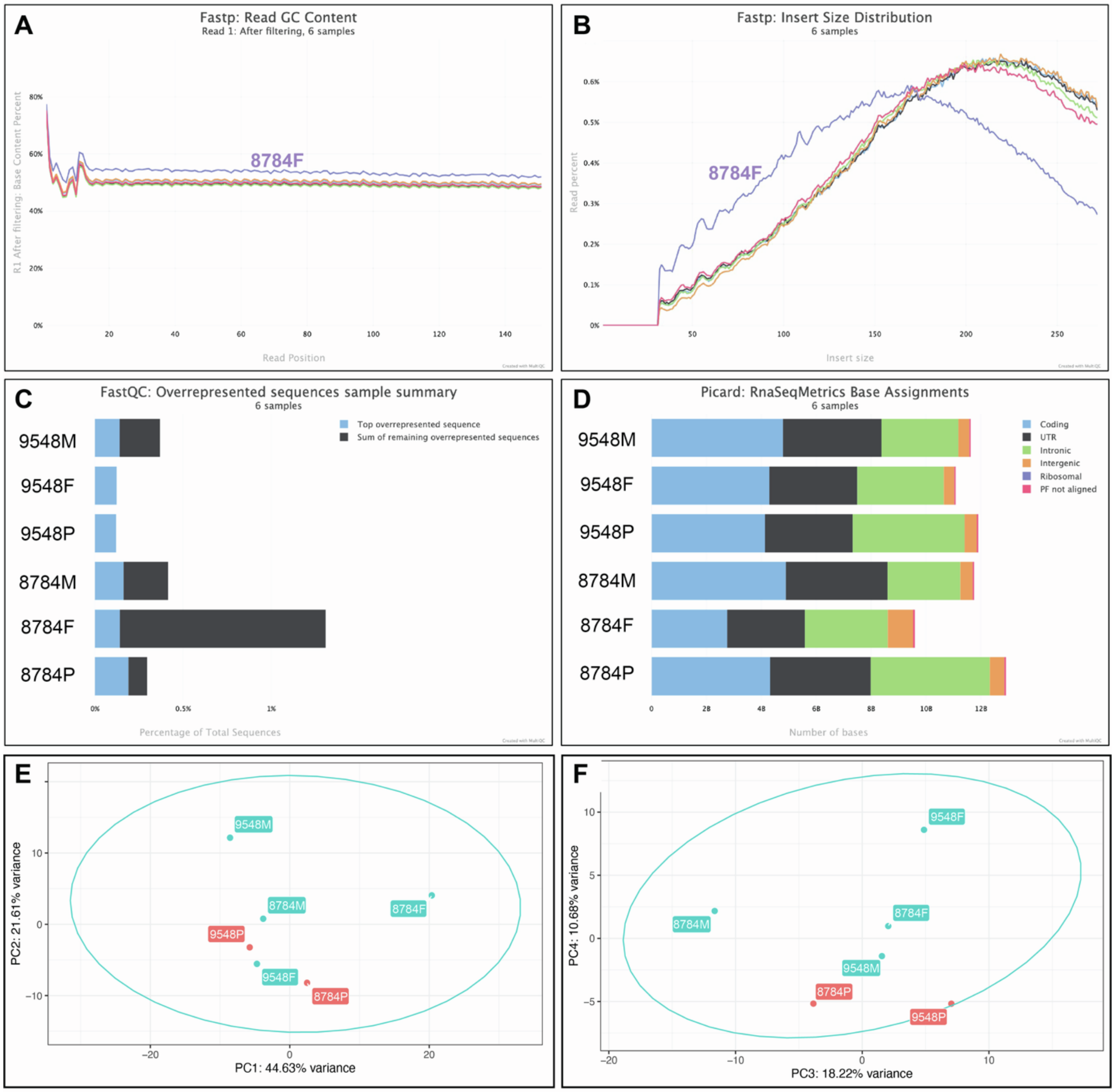

Figure S4. Quality metrics of PBMC RNAseq data for family trios 8784 and 9548. A) Fastp read GC content. B) Fastp insert size distribution. C) FastQC summary of overrepresented sequences. D) Picard base assignments. PCA plot of PC1 vs PC2 (E), and PC3 vs PC4 (D). Red represents affected children; blue represents unaffected parents; ellipse represents the 95% confidence interval.

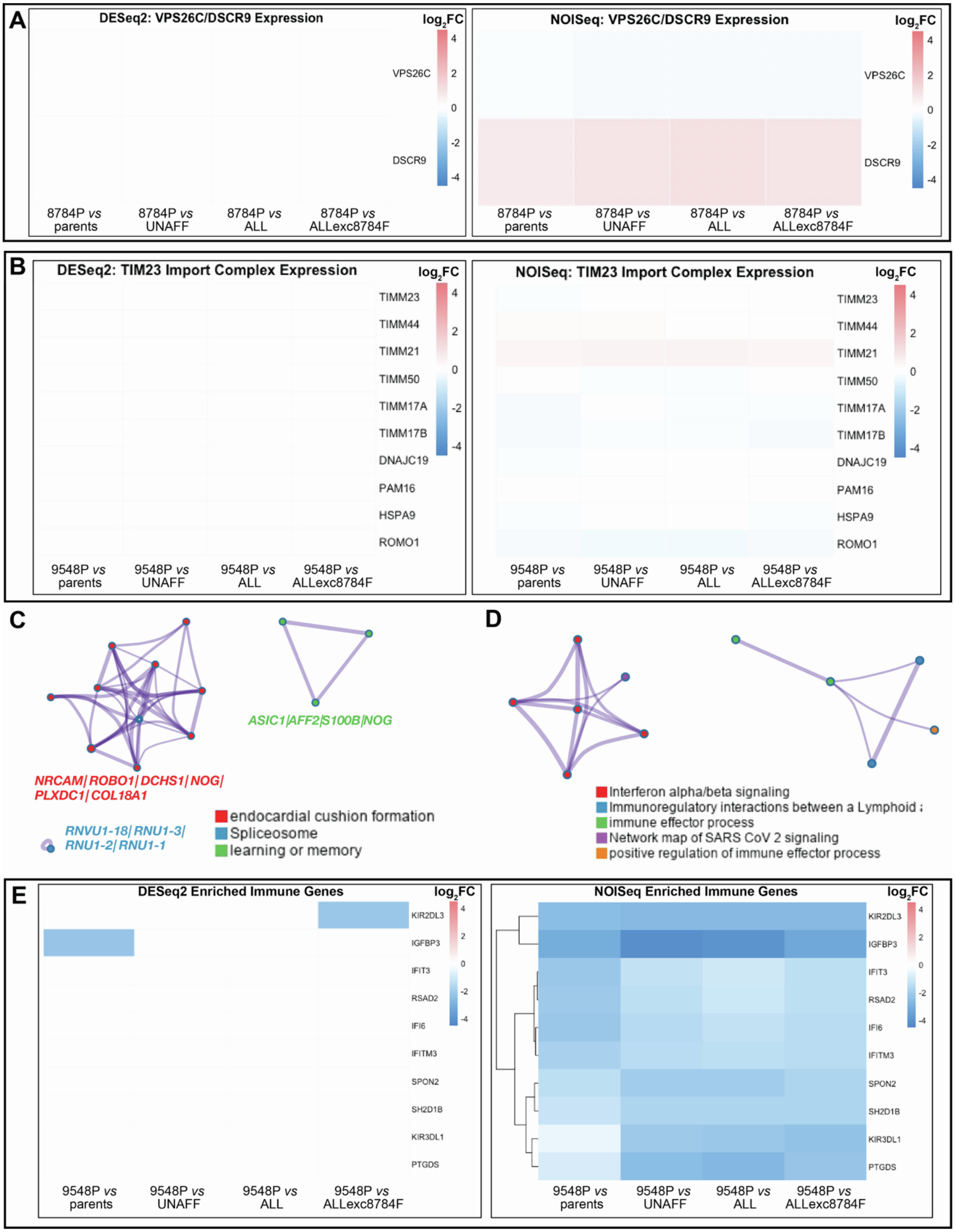

Figure S5. PBMC RNAseq findings in family trios 8784 and 9548. A) Heatmaps of DESeq2 and NOISeq Log$_2$FC data across expression comparisons for (A) *VPS26C* (and nearby lncRNA

DSCR9) in 8784P and (B) TIM23 complex members in 9548P. Comparisons: UNAFF, all unaffected adults; ALL, all other samples; ALLexc8784F, all other samples excluding 8784F. (C) MetaScape network graphs of enriched pathway and processes in genes showing elevated (C) and reduced (D) expression in 9548P PBMCs compared to unaffected PBMCs. (E) Heatmaps of DESeq2 and NOISeq Log_2FC data across expression comparisons of negatively enriched immune signaling genes identified in the MetaScape enrichment analysis.

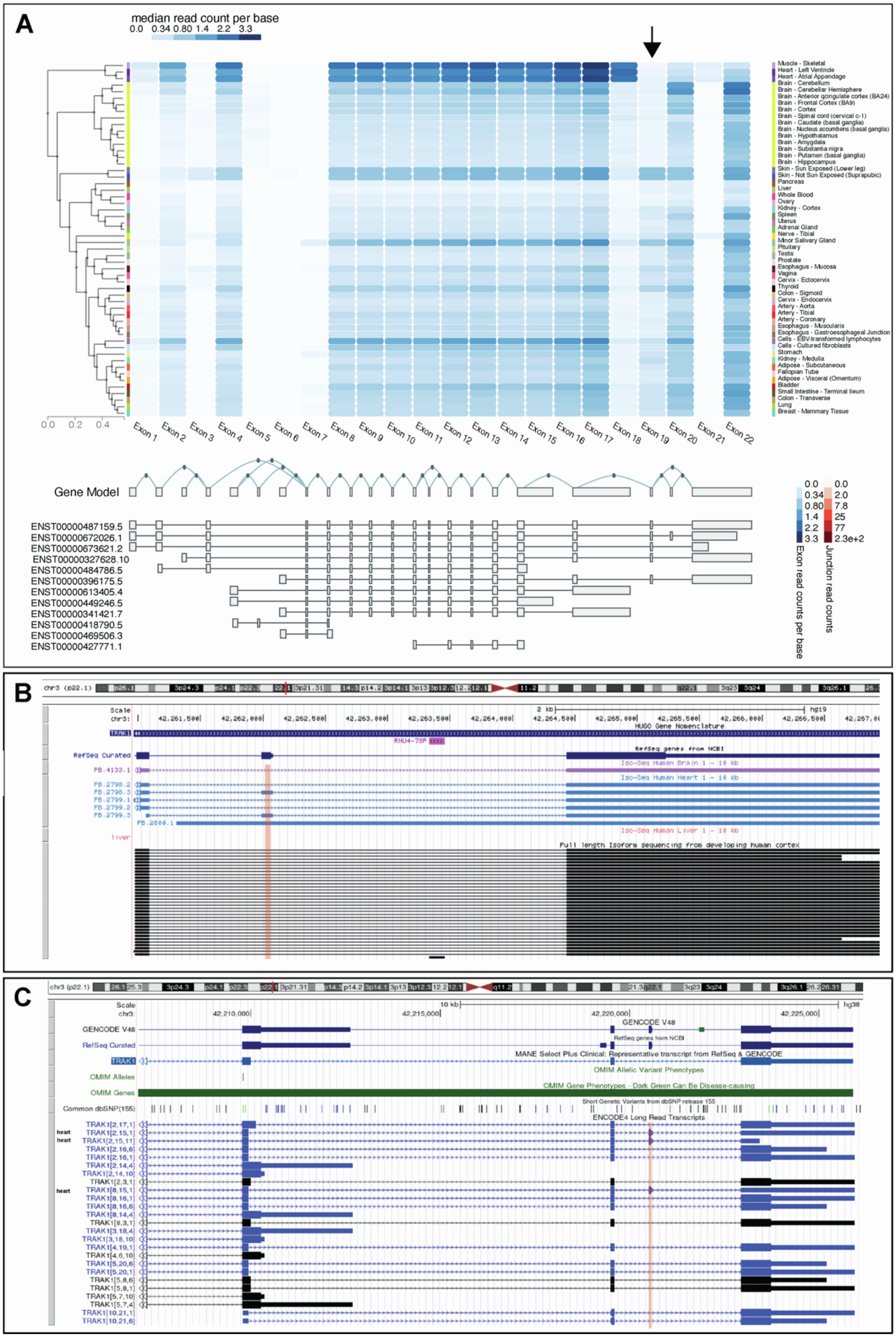
A
median read count per base
0.0 0.34 0.80 1.4 2.2 3.3
Muscle - Skeletal
Heart - Left Ventricle
Heart - Atrial Appendage
Brain - Cerebellum
Brain - Cerebellar Hemisphere
Brain - Anterior cingulate cortex (BA24)
Brain - Frontal Cortex (BA9)
Brain - Cortex
Brain - Spinal cord (cervical c-1)
Brain - Caudate (basal ganglia)
Brain - Nucleus accumbens (basal ganglia)
Brain - Hypothalamus
Brain - Amygdala
Brain - Substantia nigra
Brain - Putamen (basal ganglia)
Brain - Hippocampus
Skin - Sun Exposed (Lower leg)
Skin - Not Sun Exposed (Suprapubic)
Pancreas
Liver
Whole Blood
Ovary
Kidney - Cortex
Spleen
Uterus
Adrenal Gland
Nerve - Tibial
Minor Salivary Gland
Pituitary
Testis
Prostate
Esophagus - Mucosa
Vagina
Cervix - Ectocervix
Thyroid
Colon - Sigmoid
Cervix - Endocervix
Artery - Aorta
Artery - Tibial
Artery - Coronary
Esophagus - Muscularis
Esophagus - Gastroesophageal Junction
Cells - EBV-transformed lymphocytes
Cells - Cultured fibroblasts
Stomach
Kidney - Medulla
Adipose - Subcutaneous
Fallopian Tube
Adipose - Visceral (Omentum)
Bladder
Small Intestine - Terminal Ileum
Colon - Transverse
Lung
Breast - Mammary Tissue
0.0 0.2 0.4
Exon 1 Exon 2 Exon 3 Exon 4 Exon 5 Exon 6 Exon 7 Exon 8 Exon 9 Exon 10 Exon 11 Exon 12 Exon 13 Exon 14 Exon 15 Exon 16 Exon 17 Exon 18 Exon 19 Exon 20 Exon 21 Exon 22
Gene Model
ENST00000487159.5
ENST00000672026.1
ENST00000673621.2
ENST00000327628.10
ENST00000484786.5
ENST00000396175.5
ENST00000613405.4
ENST00000449246.5
ENST00000341421.7
ENST00000418790.5
ENST00000469506.3
ENST00000427771.1
0.0 0.34 0.80 1.4 2.2 3.3
Exon read counts per base
0.0 2.0 7.8 25 77 2.3e+2
Junction read counts
B
chr3 (p22.1)
Scale
chr3:
2 kb
hg19
42,261,500 42,262,000 42,262,500 42,263,000 42,263,500 42,264,000 42,264,500 42,265,000 42,265,500 42,266,000 42,266,500 42,267,00
HUGO Gene Nomenclature
TRAK1
RNU4-78P
RefSeq genes from NCBI
RefSeq Curated
Iso-Seq Human Brain 1 - 10 kb
PB.4133.1
Iso-Seq Human Heart 1 - 10 kb
PB.2798.2
PB.2798.3
PB.2799.1
PB.2799.2
PB.2799.3
PB.2800.1
Iso-Seq Human Liver 1 - 10 kb
liver
Full length Isoform sequencing from developing human cortex
C
chr3 (p22.1)
Scale
chr3:
10 kb
hg38
42,210,000 42,215,000 42,220,000 42,225,000
GENCODE V48
GENCODE V48
RefSeq Curated
RefSeq genes from NCBI
TRAK1
MANE Select Plus Clinical: Representative transcript from RefSeq & GENCODE
OMIM Alleles
OMIM Allelic Variant Phenotypes
OMIM Genes
OMIM Gene Phenotypes - Dark Green Can Be Disease-causing
Common dbSNP(155)
Short Genetic Variants from dbSNP release 155
ENCODE4 Long Read Transcripts
TRAK1[2,17,1]
heart TRAK1[2,15,1]
heart TRAK1[2,15,11]
TRAK1[2,16,6]
TRAK1[2,16,1]
TRAK1[2,14,4]
TRAK1[2,14,10]
TRAK1[2,3,1]
heart TRAK1[8,15,1]
TRAK1[8,16,1]
TRAK1[8,16,6]
TRAK1[8,14,4]
TRAK1[8,3,1]
TRAK1[3,18,4]
TRAK1[3,18,10]
TRAK1[4,19,1]
TRAK1[4,6,10]
TRAK1[5,20,6]
TRAK1[5,20,1]
TRAK1[5,8,6]
TRAK1[5,8,1]
TRAK1[5,7,10]
TRAK1[5,7,4]
TRAK1[10,21,1]
TRAK1[10,21,6]

Figure S6. Expression data of *TRAK1* alternative isoform predicted to harbor NM_001349247.2:c.2079G>C, p.(Glu693Asp) in individual 8779C. A) Heat map of exon expression as median read counts per base for TRAK1 isoforms across different adult tissue types in the Adult Genotype Tissue Expression (GTEx) database, with isoform maps illustrated below.[1] Black arrow pointing to exon 19 which harbors NM_001349247.2:c.2079. UCSC Genome Browser screenshot surrounding NM_001349247.2:c.2079 region (red box) in PacBio Iso-Seq data from human brain, heart, and liver as well as developing cortex[2] in hg19 (B) and (C) the ENCODE long-read transcript dataset in hg38.[3]

Materials and Methods

Participant Recruitment and Informed Consent Procedures

All study participants were identified via their medical provider, or if they had previously participated in other suspected genetic disorder studies, at Connecticut Children's (CC). Participant eligibility was determined by CC and The Jackson Laboratory for Genomic Medicine (JGM) investigators based on the following criteria: age (21 or under), medical and family history related to a suspected inherited disorder, and inconclusive prior genetic testing findings. Participants under 18 years of age were approached only if parent(s) were present. If eligible and interested, parents or adult participants were then referred to a genetic counselor within the study team for pre-enrollment counseling.

Following pre-enrollment counseling, interested parents or adult participants were provided a copy of the IRB-approved Informed Consent Form/HIPAA Authorization form (ICF) followed by a consent discussion. A designated member of the study team reviewed the ICF section by section in a private area at CC or via phone based on participant preference, encouraging questions and providing thorough responses. Parents who elected to participate with their child also completed a separate form.

Once complete, participants were asked if they wanted to join the study and were given the option to receive clinical findings (if any) after confirmation through a clinically ordered confirmatory test. Results not clinically confirmed were not returned. Results of confirmatory testing were entered into the affected participant's medical record and shared with their provided clinical care provider. A signed copy of the ICF by both participants and study team member was provided to all participants. Informed consent included the authorization for the use and disclosure of their Protected Health Information collected for use in this study by both CC and JGM study investigators. All enrolled participants were assigned a unique participant identifier (PID). The

original signed ICF was stored separately from the research record and with other study documents that contain personal identifiers.

Genome sequencing and analysis

Venous whole blood samples (at least 2 mL) were obtained from all consented subjects at CC or at Quest Diagnostics—CLIA/CAP-certified clinical laboratory and a contracted service provider for CC—using standard collection procedures and EDTA tubes. Labeled tubes were then securely transported to JGM within 24-48 hours of collection. Research trio/quad LR-GS and singleton SR-GS were performed at The Jackson Laboratory for Genomic Medicine.

High-molecular genomic DNA (gDNA) was extracted from whole blood using the PreAnalytix PAXgene Blood DNA kit (Cat. #761133) or the PacBio Nanobind PanDNA kit (PN: 103-260-000) (Table S2), according to the manufacturer's instructions. DNA concentration was measured by Qubit fluorometer with the Qubit 1X dsDNA HS Assay Kit (Thermo Fisher Scientific, Q33230). DNA integrity and fragment size distribution were assessed using the Agilent Femto Pulse system with the gDNA 165 kb Analysis Kit (Agilent FP-1002-0275). Samples with a genome quality number (GQN) ≥ 7.0 at 10 kb were selected for HiFi library preparation. For Single Molecule, Real-Time (SMRT) sequencing on the Pacific Biosciences platform, high–molecular weight genomic DNA (gDNA) was extracted from whole blood using the PreAnalytiX PAXgene Blood DNA Kit (Cat. #761133) or the PacBio Nanobind PanDNA Kit (PN: 103-260-000) (Table S2), according to the manufacturers' instructions.

DNA concentration was measured using a Qubit fluorometer with the Qubit 1X dsDNA HS Assay Kit (Thermo Fisher Scientific, Q33230). DNA integrity and fragment size distribution were assessed using the Agilent Femto Pulse system with the gDNA 165 kb Analysis Kit (Agilent FP-1002-0275). Samples with a genome quality number (GQN) ≥ 7.0 at 10 kb were selected for HiFi library preparation. Genomic DNA was sheared to a target fragment size of 16–18 kb using the Megaruptor 3 system (Diagenode, B06010003). HiFi SMRTbell libraries were prepared using the

SMRTbell Template Prep Kit 3.0 (PacBio, 102-182-700) and barcoded with SMRTbell Barcoded Adapters (PacBio, 102-009-200) to enable multiplexed sequencing. Libraries were size selected using the PippinHT system (Sage Science) to enrich for library fragments >10 kb. followed by purification, quantification, and size distribution assessment. Final size-selected HiFi SMRTbell libraries were sequenced on the PacBio Revio sequencing platform to generate highly accurate HiFi long reads achieving 30X genome coverage for comprehensive genomic analysis. For SR-GS, gDNA was fragmented and ligated to unique index adapters using an Illumina DNA PCR-free library preparation (Cat. 20041794). The library was assessed for quality using Qubit (Qubit HSDNA kit; ThermoFisher #Q32854), TapeStation (Agilent D1000 High Sensitivity Reagents #5067-5585), qPCR (KAPA library quantification universal kit; Roche #7960336001). High quality libraries were sequenced on an Illumina S2 flow cell using an Illumina NovaSeq 6000 instrument (150-bp paired-end reads) for 30X target mean coverage.

LR-GS analysis was performed on Revio fastq and unaligned bam output files with 5mC kinetics turned on using custom, in-house secondary and tertiary analysis pipelines with phased-assembly and read-based analysis workflows. In-house pipelines were run using custom scripts on the Google Cloud Platform. For the *de novo* assembly-based pipeline, trio-informed phased-assembly was performed using Hifiasm with Trio binning (v0.20.0), followed by variant calling with PAV (v2.3.4) and variant prioritization by Human Phenotype Ontology (HPO) terms using SvAnna (v1.0.5)[4,5]. For PAV, calls were made from the phased assembly against the no-alternative (no-ALT) hg38 reference genome assembly by Human Genome Structural Variation (HGSV) (ftp://ftp.1000genomes.ebi.ac.uk/vol1/ftp/data_collections/HGSVC2/technical/reference/20200513_hg38_NoALT/). For the alignment-based workflow, pbmm2 (v1.13.1) was used to align reads to the no-ALT hg38 reference. For 2990P and 2990M, alignment to the hs1 (T2T CHM13-v2.0) reference was also performed. Read-based phasing was then performed using DeepVariant (v1.6.1) and WhatsHap (v2.3)[6,7]. Small variant calling was performed with Deepvariant (v1.6.1)[6]. Structural variants were called with PacBio pbsv (v2.9.0) and Sniffles2 (v2.3.3) [8]. Quality control

analysis was performed using Nanoplot (v1.42.0) and Samtools (v1.19). CNVs were called with HiFiCNV (v1.0.0). Repeat expansions in the following 30 clinically relevant loci were called using TRGT (v1.0.0): *AFF2, AR, ATN1, ATXN1, ATXN10, ATXN2, ATXN3, ATXN7, ATXN8OS, C9ORF72, CACNA1A, CNBP, CSTB, DIP2B, DMPK, FMR1, FXN, GIPC1, GLS, HTT, JPH3, NOP56, PABPN1, PHOX2B, PPP2R2B, RFC1, STARD7, TBP, TCF4,* and *FGF14.* Variants in the following 16 clinically relevant high identity paralogs were called with Paraphase (v3.1.1): *SMN1-2, CYP21A2, TNXB, C4A/C4B, PMS2, STRC, IKBKG, NCF1, NEB, F8, CFC1, OPN1LW/OPN1MW, HBA1-2, GBA, CYP11B1-2,* and *CFH/CFHR1-4.* Population filtering was performed with SVAFotate (v0.2.0) [9] against the following LR- and SR-GS population databases, using a reciprocal overlap of 80%: Human Genome Structural Variation Consortium (HGSVC) versions 2-3[10,11], Consortium of Long Read Sequencing (CoLoRS) (colorsdb.org), Genome Answers for Kids (GA4K)[12], Trans-Omics for Precision Medicine (TOPMed),[13] Genome Aggregation Database (gnomAD)[14], 1000 Genomes[14], and the National Human Genome Research Institute Centers for Common Disease Genomics (NHGRI-CCDG) program[15]. For more information on the LR-GS pipeline, please see https://github.com/TheJacksonLaboratory/jax-apml-lrs.

Variant prioritization by HPO terms was performed using SvAnna (v1.0.4)[4,5]. Tertiary analysis was performed via ingestion of DeepVariant, pbsv, and PAV VCFs into Illumina Emedgene Software (v36.7.0). HPO terms for the proband were added and each family was analyzed as a trio or quad for segregation analysis. All 'candidate' and 'most likely' candidate variants annotated by Emedgene's explainable AI underwent variant interpretation. In addition, all variants present in several user-defined filter presets, including variants not flagged by Emedgene, were reviewed. Lastly, variant interpretation was performed on SvAnna outputs using a pathogenicity of structural variation (psv) score of >= 2 for the following tools: Sniffles2, pbsv, and PAV. Small variant (SNVs and indels) concordance analysis was performed using vcfeval within RTG Tools v3.12.1 (https://github.com/RealTimeGenomics/rtg-tools).

To benchmark the performance of the LR-GS pipeline, GIAB sample HG002 (NA24385) was sequenced to a mean coverage of 29.9X and analyzed (Table S2). Concordance of small variants (SNVs, indels) and SVs was evaluated for assembly- (PAV) and read-based (Sniffles2, pbsv, DeepVariant) variant callers. Small variant (SNVs and indels) concordance analysis was performed using vcfeval within RTG Tools v3.12.1 (https://github.com/RealTimeGenomics/rtg-tools) against the Genome-In-A-Bottle (GIAB) HG002 benchmark for small variants (GRCh38 v4.2.1)[16]. SV concordance was performed using Truvari v4.0.0 against the HGSVC3 callset for SVs[11] omitting low-confidence regions in GRCh38[10]. This approach was taken to avoid liftover operations from GRCh37 that could skew results for SVs (Figure S1). In addition, HG002 SV calls were also compared to the GIAB challenging medically relevant gene (CMRG) regions benchmark[17].

For SR-GS, output sequencing data were converted from BCL to FASTQ format using BCLConvert v4.2.7 within Illumina Connected Analytics. Alignment to the human reference genome GRCh38, variant calling, and annotation were performed using Illumina DRAGEN Germline Genome pipeline v10 within Emedgene (v36.7.0). Phenotype prioritization for both LR-GS and SR-GS pipelines was performed using the HPO terms provided in Table S1. Variants were interpreted using ACMG guidelines [18]. All variants were visually inspected in Integrative Genomics Viewer (IGV) v2.16.0.

RNA sequencing and analysis

PBMCs were isolated from whole blood samples collected from 8784 and 9548 family trios. Total RNA was extracted from ~9 million PBMCs per sample using Qiagen All prep RNA kit (#80404) according to manufacturer's instructions. Samples were ribo-depleted followed by 151 bp paired-end sequencing on the Illumina NovaSeq X Plus, 10B flow cell, ~30 million reads per sample. ERCC spike-ins (Invitrogen #4456740) were added as sequencing controls with starting concentrations according to the manufacturer's instructions (100 ng input with 1:500 dilution of

ERCC spiked in). Library preparation and sequencing were performed by the Genome Technologies Laboratory at The Jackson Laboratory for Genomic Medicine.

FASTQ preprocessing was performed using fastp (v0.23.2) (https://github.com/OpenGene/fastp). Read quality was assessed by FASTQC[19] (v0.11.9) (http://www.bioinformatics.babraham.ac.uk/projects/fastqc) and visualized with MultiQC[20] (v1.25.2) (https://github.com/MultiQC/MultiQC) (Figure S3). FASTQ reads were mapped to GRCh38.p13 reference genome with GTF annotation files as well as to the ERCC spike-in FASTA and GTF annotation files using STAR alignment (https://github.com/alexdobin/STAR). Gene and isoform expression counts were obtained using RSEM[21] (https://deweylab.github.io/RSEM). Principal component analysis (PCA) was performed on raw gene count data using pcaExplorer[22] package in R (v4.5.0). Based on PCA results, ERCC spike-in counts were used for normalization in differential expression analysis.

Given the lack of biological replicates for this analysis, differential expression analysis was performed using two computational approaches: DESeq2[23] (v1.49.4) and NOISeq[24] (v2.53.0). DESeq2 uses parametric negative binomial distribution modeling and requires biological replicates for accurate dispersion estimation with empirical Bayes shrinking. Since no biological replicates were present, we focused on hits with a $\log_2$ fold change (FC) value of ≥ |1| to explore expression trends. Conversely, NOISeq is a nonparametric tool that utilizes empirical noise modeling and does not require biological replicates when using the NOISeq-sim function, which can simulate noise for no-replicate datasets. Probabilistic score (p) of > 0.9 and $\log_2$FC > |1| were considered for analysis. To optimize information obtained from no-replicate data, and potentially reduce age or outlier effects, the following four comparisons were performed using DESeq2 and NOISeq for each trio: (1) proband (8784P or 9548P) vs parents, (2) proband vs unaffected adults (8784M/F, 9548M/F), (3) proband vs all other samples (8784M/F, 9548M/F, and 8784P or 9548P), (4) proband vs all other samples excluding 8784F (8784M, 9548M/F, and 8784P or 9548P) due to potential outlier effects as suggested by PCA and QC data. DESeq2 hits with $\log_2$FC ≥ |1| (and

NOISeq hits with $\log_2FC \geq |1|$ plus $p > 0.9$) were merged across all four comparisons and approach per proband establishing a candidate list, and any hits that were also present in the other proband's candidate list were removed. Pathway and process enrichment analysis were performed on these candidate gene lists using MetaScape (https://metascape.org/)[25]. Plots were created using ggplot2 and pheatmap packages in R.

Computational tools and online resources

Combined Annotation Dependent Depletion (CADD) Phred scores were obtained using CADD v1.7 against GRCh38 (https://cadd.gs.washington.edu). MetaDome analysis was performed on primary transcripts for research findings using the online tool (stuart.radboudumc.nl/metadome).[26] The following additional *in silico* and deep learning tools were used for variant effect predictions against GRCh38 (Table S4): AlphaMissense (https://alphamissense.hegelab.org/)[27], PolyPhen-2 (http://genetics.bwh.harvard.edu/pph2/)[28], SpliceAI (https://spliceailookup.broadinstitute.org/)[29], PrimateAI (https://github.com/Illumina/PrimateAI)[30], and DDMut (https://biosig.lab.uq.edu.au/ddmut/)[31]. Visualization of the *H. sapiens* AlphaFold[32] model AF-O14925 F1 and *S. cerevisiae* TIM23 Complex cryogenic electron microscopy (cryo-EM) structure was performed using the PDB:8E1M model[33] in PyMOL (v2.5.2). Disease information was obtained from the Online Mendelian Inheritance in Man (OMIM, http://www.omim.org). Protein information was obtained from Uniprot.org.

References

1. Consortium, G. (2013). The Genotype-Tissue Expression (GTEx) project. Nat Genet *45*, 580-585. 10.1038/ng.2653.
2. Patowary, A., Zhang, P., Jops, C., Vuong, C.K., Ge, X., Hou, K., Kim, M., Gong, N., Margolis, M., Vo, D., et al. (2023). Developmental isoform diversity in the human neocortex informs neuropsychiatric risk mechanisms. bioRxiv. 10.1101/2023.03.25.534016.
3. Reese, F., Williams, B., Balderrama-Gutierrez, G., Wyman, D., Çelik, M.H., Rebboah, E., Rezaie, N., Trout, D., Razavi-Mohseni, M., Jiang, Y., et al. (2023). The ENCODE4 long-read RNA-seq collection reveals distinct classes of transcript structure diversity. bioRxiv. 10.1101/2023.05.15.540865.
4. Robinson, P.N., Kohler, S., Oellrich, A., Sanger Mouse Genetics, P., Wang, K., Mungall, C.J., Lewis, S.E., Washington, N., Bauer, S., Seelow, D., et al. (2014). Improved exome prioritization of disease genes through cross-species phenotype comparison. Genome Res *24*, 340-348. 10.1101/gr.160325.113.
5. Danis, D., Jacobsen, J.O.B., Balachandran, P., Zhu, Q., Yilmaz, F., Reese, J., Haimel, M., Lyon, G.J., Helbig, I., Mungall, C.J., et al. (2022). SvAnna: efficient and accurate pathogenicity prediction of coding and regulatory structural variants in long-read genome sequencing. Genome Med *14*, 44. 10.1186/s13073-022-01046-6.
6. Poplin, R., Chang, P.C., Alexander, D., Schwartz, S., Colthurst, T., Ku, A., Newburger, D., Dijamco, J., Nguyen, N., Afshar, P.T., et al. (2018). A universal SNP and small-indel variant caller using deep neural networks. Nat Biotechnol *36*, 983-987. 10.1038/nbt.4235.
7. Martin, M., Ebert, P., and Marschall, T. (2023). Read-Based Phasing and Analysis of Phased Variants with WhatsHap. Methods Mol Biol *2590*, 127-138. 10.1007/978-1-0716-2819-5_8.
8. Smolka, M., Paulin, L.F., Grochowski, C.M., Horner, D.W., Mahmoud, M., Behera, S., Kalef-Ezra, E., Gandhi, M., Hong, K., Pehlivan, D., et al. (2024). Detection of mosaic and population-level structural variants with Sniffles2. Nat Biotechnol. 10.1038/s41587-023-02024-y.
9. Nicholas, T.J., Cormier, M.J., and Quinlan, A.R. (2022). Annotation of structural variants with reported allele frequencies and related metrics from multiple datasets using SVAFotate. BMC Bioinformatics *23*, 490. 10.1186/s12859-022-05008-y.
10. Ebert, P., Audano, P.A., Zhu, Q., Rodriguez-Martin, B., Porubsky, D., Bonder, M.J., Sulovari, A., Ebler, J., Zhou, W., Serra Mari, R., et al. (2021). Haplotype-resolved diverse human genomes and integrated analysis of structural variation. Science *372*. 10.1126/science.abf7117.
11. Logsdon, G.A., Ebert, P., Audano, P.A., Loftus, M., Porubsky, D., Ebler, J., Yilmaz, F., Hallast, P., Prodanov, T., Yoo, D., et al. (2025). Complex genetic variation in nearly complete human genomes. Nature *644*, 430-441. 10.1038/s41586-025-09140-6.
12. Cohen, A.S.A., Farrow, E.G., Abdelmoity, A.T., Alaimo, J.T., Amudhavalli, S.M., Anderson, J.T., Bansal, L., Bartik, L., Baybayan, P., Belden, B., et al. (2022). Genomic answers for children: Dynamic analyses of >1000 pediatric rare disease genomes. Genet Med *24*, 1336-1348. 10.1016/j.gim.2022.02.007.
13. Taliun, D., Harris, D.N., Kessler, M.D., Carlson, J., Szpiech, Z.A., Torres, R., Taliun, S.A.G., Corvelo, A., Gogarten, S.M., Kang, H.M., et al. (2021). Sequencing of 53,831 diverse genomes from the NHLBI TOPMed Program. Nature *590*, 290-299. 10.1038/s41586-021-03205-y.
14. Koenig, Z., Yohannes, M.T., Nkambule, L.L., Zhao, X., Goodrich, J.K., Kim, H.A., Wilson, M.W., Tiao, G., Hao, S.P., Sahakian, N., et al. (2024). A harmonized public resource of

deeply sequenced diverse human genomes. Genome Res *34*, 796-809. 10.1101/gr.278378.123.

15. Abel, H.J., Larson, D.E., Regier, A.A., Chiang, C., Das, I., Kanchi, K.L., Layer, R.M., Neale, B.M., Salerno, W.J., Reeves, C., et al. (2020). Mapping and characterization of structural variation in 17,795 human genomes. Nature *583*, 83-89. 10.1038/s41586-020-2371-0.
16. Wagner, J., Olson, N.D., Harris, L., Khan, Z., Farek, J., Mahmoud, M., Stankovic, A., Kovacevic, V., Yoo, B., Miller, N., et al. (2022). Benchmarking challenging small variants with linked and long reads. Cell Genom *2*. 10.1016/j.xgen.2022.100128.
17. Wagner, J., Olson, N.D., Harris, L., McDaniel, J., Cheng, H., Fungtammasan, A., Hwang, Y.C., Gupta, R., Wenger, A.M., Rowell, W.J., et al. (2022). Curated variation benchmarks for challenging medically relevant autosomal genes. Nat Biotechnol *40*, 672-680. 10.1038/s41587-021-01158-1.
18. Richards, S., Aziz, N., Bale, S., Bick, D., Das, S., Gastier-Foster, J., Grody, W.W., Hegde, M., Lyon, E., Spector, E., et al. (2015). Standards and guidelines for the interpretation of sequence variants: a joint consensus recommendation of the American College of Medical Genetics and Genomics and the Association for Molecular Pathology. Genet Med *17*, 405-424. 10.1038/gim.2015.30.
19. Wingett, S.W., and Andrews, S. (2018). FastQ Screen: A tool for multi-genome mapping and quality control. F1000Res *7*, 1338. 10.12688/f1000research.15931.2.
20. Ewels, P., Magnusson, M., Lundin, S., and Käller, M. (2016). MultiQC: summarize analysis results for multiple tools and samples in a single report. Bioinformatics *32*, 3047-3048. 10.1093/bioinformatics/btw354.
21. Li, B., and Dewey, C.N. (2011). RSEM: accurate transcript quantification from RNA-Seq data with or without a reference genome. BMC Bioinformatics *12*, 323. 10.1186/1471-2105-12-323.
22. Marini, F., and Binder, H. (2019). pcaExplorer: an R/Bioconductor package for interacting with RNA-seq principal components. BMC Bioinformatics *20*, 331. 10.1186/s12859-019-2879-1.
23. Love, M.I., Huber, W., and Anders, S. (2014). Moderated estimation of fold change and dispersion for RNA-seq data with DESeq2. Genome Biol *15*, 550. 10.1186/s13059-014-0550-8.
24. Tarazona, S., García-Alcalde, F., Dopazo, J., Ferrer, A., and Conesa, A. (2011). Differential expression in RNA-seq: a matter of depth. Genome Res *21*, 2213-2223. 10.1101/gr.124321.111.
25. Zhou, Y., Zhou, B., Pache, L., Chang, M., Khodabakhshi, A.H., Tanaseichuk, O., Benner, C., and Chanda, S.K. (2019). Metascape provides a biologist-oriented resource for the analysis of systems-level datasets. Nat Commun *10*, 1523. 10.1038/s41467-019-09234-6.
26. Wiel, L., Baakman, C., Gilissen, D., Veltman, J.A., Vriend, G., and Gilissen, C. (2019). MetaDome: Pathogenicity analysis of genetic variants through aggregation of homologous human protein domains. Hum Mutat *40*, 1030-1038. 10.1002/humu.23798.
27. Minton, K. (2023). Predicting variant pathogenicity with AlphaMissense. Nat Rev Genet *24*, 804. 10.1038/s41576-023-00668-9.
28. Adzhubei, I., Jordan, D.M., and Sunyaev, S.R. (2013). Predicting functional effect of human missense mutations using PolyPhen-2. Curr Protoc Hum Genet *Chapter 7*, Unit7.20. 10.1002/0471142905.hg0720s76.
29. Jaganathan, K., Kyriazopoulou Panagiotopoulou, S., McRae, J.F., Darbandi, S.F., Knowles, D., Li, Y.I., Kosmicki, J.A., Arbelaez, J., Cui, W., Schwartz, G.B., et al. (2019). Predicting Splicing from Primary Sequence with Deep Learning. Cell *176*, 535-548.e524. 10.1016/j.cell.2018.12.015.

30. Sundaram, L., Gao, H., Padigepati, S.R., McRae, J.F., Li, Y., Kosmicki, J.A., Fritzilas, N., Hakenberg, J., Dutta, A., Shon, J., et al. (2018). Predicting the clinical impact of human mutation with deep neural networks. Nat Genet *50*, 1161-1170. 10.1038/s41588-018-0167-z.
31. Zhou, Y., Pan, Q., Pires, D.E.V., Rodrigues, C.H.M., and Ascher, D.B. (2023). DDMut: predicting effects of mutations on protein stability using deep learning. Nucleic Acids Res *51*, W122-W128. 10.1093/nar/gkad472.
32. Jumper, J., Evans, R., Pritzel, A., Green, T., Figurnov, M., Ronneberger, O., Tunyasuvunakool, K., Bates, R., Žídek, A., Potapenko, A., et al. (2021). Highly accurate protein structure prediction with AlphaFold. Nature *596*, 583-589. 10.1038/s41586-021-03819-2.
33. Sim, S.I., Chen, Y., Lynch, D.L., Gumbart, J.C., and Park, E. (2023). Structural basis of mitochondrial protein import by the TIM23 complex. Nature *621*, 620-626. 10.1038/s41586-023-06239-6.
