# Supplementary material for: Diagnostic utility of clinical genome reanalysis in rare pediatric disorders using long-read sequencing
Source: HGG Adv. 2026 Apr 27;7(3):100620. doi: 10.1016/j.xhgg.2026.100620 (PMC13202554; doi:10.1016/j.xhgg.2026.100620)
Supplement: Document S2. Article plus supplemental information [file mmc3.pdf]

HGG Advances

REPORT

Diagnostic utility of clinical genome reanalysis in rare pediatric disorders using long-read sequencing

Elizabeth A. Werren,[1] Purva Vats,[1] Gabriel E. Rech,[1] Michael Peracchio,[2] Cameron King,[3] Elizabeth J. Charnysh,[1] Ryan D. Gorham,[1] Peter A. Audano,[1] Peter N. Robinson,[1] Melissa A. Kelly,[1] Adam P. Matson,[4,5,6] Mark D. Adams,[1,8,*] and Louisa Kalsner[2,4,7,*]

Summary

Over half of presumed genetic disease cases remain undiagnosed following short-read exome sequencing (SR-ES) or genome sequencing (SR-GS). Long-read GS (LR-GS) shows promise for uncovering etiologies missed by SR genetic testing, particularly structural variants (SVs). However, SV interpretation remains challenging due to limitations in call reliability, population allele frequency estimates, and functional impact prediction. To advance clinical LR-GS implementation, we analyzed the genomes of 19 children with suspected rare genetic conditions and prior negative or inconclusive clinical SR-GS/SR-ES as well as their parents using PacBio HiFi LR-GS. One additional family with limited DNA underwent Illumina SR-GS only, and 11 probands received SR-GS to complement small-variant detection. LR-GS data were processed using phased-assembly and read-based variant-calling pipelines validated in SV-positive control subjects, while SR-GS data were processed with the Illumina DRAGEN pipeline. Variants were prioritized using phenotype-driven approaches. Diagnostic variants (likely pathogenic or pathogenic) were identified in 2/20 (10%) families, while an additional 5/20 (25%) harbored findings of uncertain diagnostic significance, including variants of uncertain significance (VUSs) and variants in genes of uncertain significance (GUSs). All reported variants were detected independently of LR-GS by research SR-GS or by reanalysis of prior clinical SR data. Several LR-GS SV candidates were excluded after population allele frequency filtering, underscoring its importance in clinical SV interpretation. Overall, the observed 10% increase in diagnostic yield was achievable through SR analysis alone, as LR-GS was not required to identify diagnostic variants in this cohort. Functional studies are needed to clarify the clinical relevance of uncertain findings.

Developmental disorders exhibit high genetic heterogeneity, often with a constellation of non-specific features, posing major challenges for molecular diagnosis. While exome sequencing (ES) and genome sequencing (GS) have improved diagnostic yield, more than half of individuals with rare disease remain on diagnostic odysseys.[1,2] The limitation of short-read GS (SR-GS) technologies in assessing the full repertoire of disease variation, such as complex structural variants (SVs) and altered methylation, may contribute to the low diagnostic yield of genetic testing.[2,3] Long-read GS (LR-GS) is increasingly used to elucidate genetic etiologies of disease[4–8]; however, its clinical adoption remains limited, as few laboratories currently offer clinically validated LR diagnostic assays.[9] Here, we applied LR-GS with state-of-the-art tools to a small, rare disease cohort where genetic diagnosis may have been missed due to the contributions of genes of uncertain significance (GUSs), difficult-to-detect SVs, non-coding variation, and/or epigenetic alterations to disease phenotypes. Our findings demonstrate that LR-GS has the potential to resolve some of these diagnostic gaps while underscoring the need for further computational and interpretative development to fully unlock its clinical utility.

As part of the effort to assess LR-GS utility in the diagnosis of rare developmental disorders, we recruited children and young adults with a suspected genetic condition and negative prior exome or SR-GS testing, together with their biological parents (Tables 1 and S1). All study participants provided informed consent in accordance with the ethical standards for human research subjects established by the institutional review board (IRB) committees at the Connecticut Children's (CC) and The Jackson Laboratory for Genomic Medicine (JGM). See the supplemental material and methods for comprehensive details on participant recruitment, consenting, and protocols. Whole-blood samples were obtained from 19 trios, each comprising a biological mother (M), biological father (F), and affected child (proband [P]), as well as from one quad (M, F, and two affected siblings: P-1 and P-2). All affected participants presented with non-specific, syndromic conditions (Table S1; see the supplemental note). The most common phenotypes observed in the cohort include global developmental delay and/or intellectual disability (17/21, 81.0%), seizure (13/21, 61.9%), facial

[1]The Jackson Laboratory for Genomic Medicine, Farmington, CT 06032, USA; [2]Division of Genetics, Connecticut Children's, Hartford, CT 06106, USA; [3]Department of Research, Connecticut Children's, Hartford, CT 06106, USA; [4]Department of Pediatrics, University of Connecticut School of Medicine, Farmington, CT 06030, USA; [5]Division of Neonatology, Connecticut Children's, Hartford, CT 06106, USA; [6]Department of Immunology, UConn Health, Farmington, CT 06030, USA; [7]Division of Neurology, Connecticut Children's, Hartford, CT 06106, USA
[8]Lead contact
*Correspondence: mark.adams@jax.org (M.D.A.), lkalsner@connecticutchildrens.org (L.K.)
https://doi.org/10.1016/j.xhgg.2026.100620.
© 2026 The Authors. Published by Elsevier Inc. on behalf of American Society of Human Genetics.
This is an open access article under the CC BY license (http://creativecommons.org/licenses/by/4.0/).

Table 1. Summary of cohort demographics and genetic testing

**Proband demographics**	
Female, no. (%)	8/21 (38.1%)
Male, no. (%)	13/21 (61.9%)
Median age, years (IQR)	8 (10–4)
Consanguinity, no. (%)	1/21 (4.8%)
**Prior genetic testing**	**No. of probands (%)**
Short-read genome sequencing	2/21 (9.5%)
Exome sequencing	20/21 (95.2%)
Gene panel sequencing	13/21 (61.9%)
Chromosomal microarray	15/21 (71.4%)
Mitochondrial sequencing	4/21 (19.0%)
RNA sequencing	1/21 (4.8%)
**Present study testing**	**No. of families (%)**
PacBio HiFi long-read genome sequencing	
Trio	18/20 (95%)
Quad	1/20 (5%)
Proband only	0/20 (0%)
Illumina short-read genome sequencing	
Trio	1/20 (5%)
Quad	0/20 (0%)
Proband only	11/20 (55%)
RNA sequencing	
Trio	2/20 (10%)
Quad	0/20 (0%)
Proband only	0/20 (0%)

dysmorphisms (13/21, 61.9%), hypotonia (11/21, 52.4%), microcephaly (6/21, 28.6%), and failure to thrive (5/21, 23.8%) (Table S1; see the supplemental note).

PacBio HiFi LR-GS was performed on genomic DNA extracted from fresh whole blood using a Revio system on 19 families (18 trios and 1 quad); 11 of the probands' samples also underwent concurrent Illumina SR-GS. One trio (2598) yielded limited DNA and underwent Illumina SR-GS only (supplemental material and methods). Samples were sequenced to an average mean coverage of 31.2× for LR-GS and 45.5× for SR-GS (Table S2). For LR-GS analysis, an in-house pipeline was developed to leverage both phased assembly-based[10,11] and read-based[12–14] tools to call a wide range of variant types against the hg38 no-alt reference genome,[10] including single-nucleotide variants (SNVs), insertions/deletions under 50 bp (indels), SVs, and repeat expansions (Figure 1; supplemental material and methods). Benchmarking showed high performance across all callers (Figure S1; supplemental material and methods). SR-GS analysis was performed using the Illumina DRAGEN Germline Genome analysis pipeline and hg38 reference for variant calling of small variants (SNVs/indels), SVs, and repeat expansions (supplemental material and methods). All variants for LR-GS and SR-GS were prioritized using phenotype-driven filtering based on Human Phenotype Ontology (HPO) terms and interpreted according to the American College of Medical Genetics and Genomics (ACMG) and the Association for Molecular Pathology (AMP) guidelines.[15] Tertiary analysis leveraged a combination of commercial and publicly available solutions to support structured evidence review and variant annotation, including the Illumina Emedgene software (LR-GS and SR-GS) and SvAnna (LR-GS only).[16] Emedgene applies AI-driven phenotype-genotype matching algorithms to identify candidate variants from LR- and SR-called variants (SNVs, indels, repeat expansions, and SVs, excluding inversions), while SvAnna predicts pathogenicity for all LR-called SVs using an HPO-informed pathogenicity of structural variation (psv) score (supplemental material and methods).[16] All monoallelic candidate variants affecting autosomal recessive genes were further evaluated for potential variants in *trans* (including both small variants and SVs) through both expert manual review and automated compound heterozygous detection in Emedgene.

Given the growing recognition that SVs may represent a major source of disease heritability not captured by SR-GS, we first sought to ensure that our LR-GS analysis was robust for causal SV detection using previously characterized samples. To test the sensitivity of the pipeline for detecting and prioritizing known disease-causing SVs, we sequenced two positive disease control subjects with well-characterized pathogenic SVs (Coriell samples NA14734 and NA02944; Table S2). NA14734 is an individual with congenital adrenal hyperplasia due to 21-hydroxylase deficiency (MIM: 201910) with compound heterozygous deletion of the *CYP21A2* paralog, and NA02944 is an individual with DiGeorge syndrome (MIM: 188400) due to an unbalanced translocation (46,XY,+der(20)t(20;22)(q11.2;q11.2),−22.arr[hg38] 20p13p11.1(81021–26324931)x3,22q11.1q11.21(16384223–20324382)x1) (Figures 2A and 2B; Table S3). In addition, we obtained PacBio HiFi sequencing files from 5 families (PC01–PC05) harboring previously published causal SVs from the Genome Answers for Kids (GA4K) cohort,[4,8] providing a broad repertoire of SV types for testing (Tables S2 and S3). For secondary and tertiary analyses, all causal variants were detected using assembly- and/or read-based variant callers and prioritized by SvAnna and/or Emedgene tools (Table S3). The compound heterozygous deletion involving the *CYP21A2* paralog in NA14734 was detected by the Phased Assembly Variant (PAV) and Paraphase callers and highly prioritized by SvAnna (psv = 83.6) and Emedgene (AI candidate and present in preset filters). The unbalanced translocation in NA02944 was captured only by HiFiCNV, the outputs of which are not compatible with SvAnna or Emedgene. The *KMT2E* c.729+113_1359−612del (GenBank: NM_182931.3) (p.Ala244Ter)

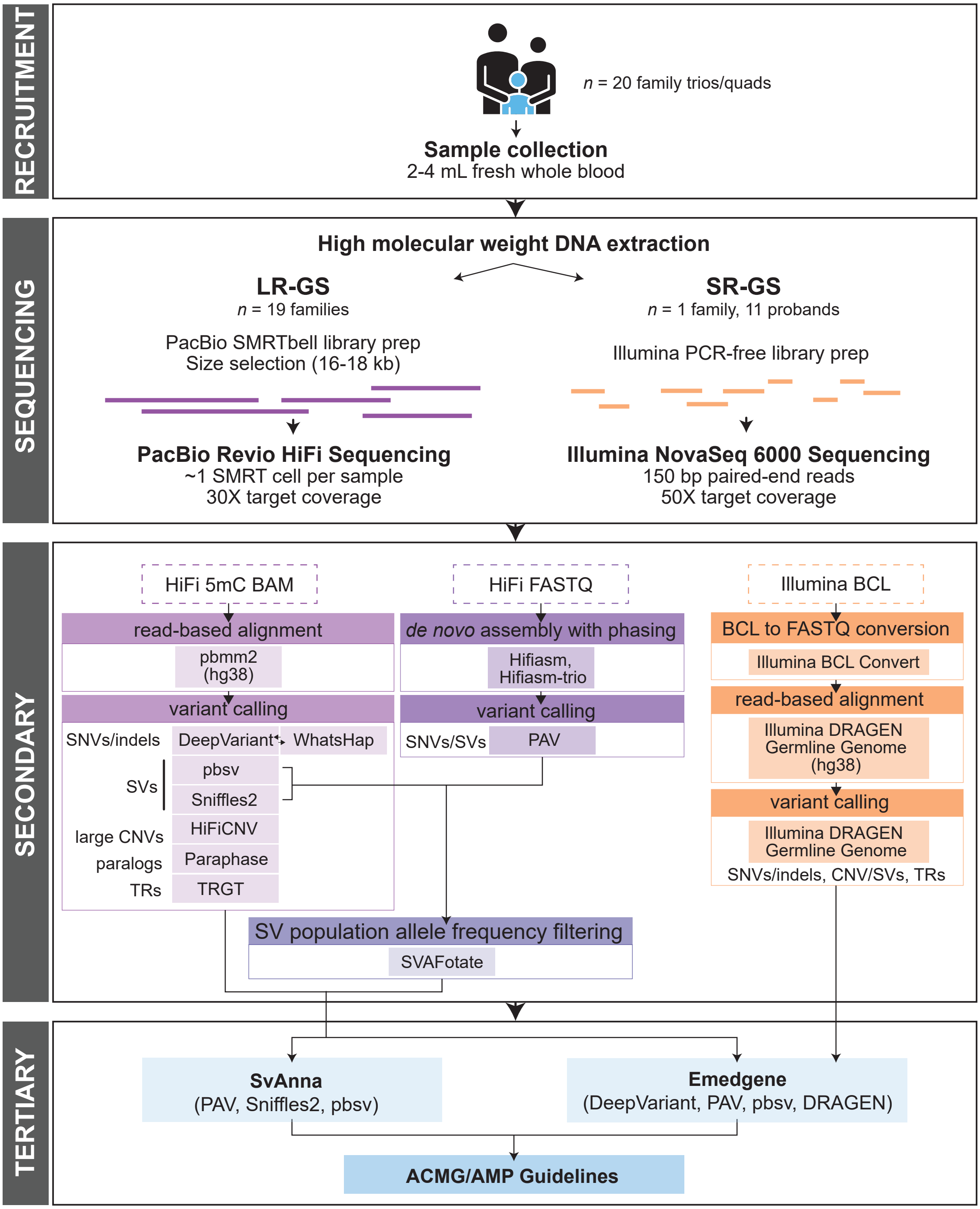

Figure 1. Diagram of study design

Twenty families (19 trios and one quad) were enrolled in the study. Whole-blood samples were collected for high-molecular-weight DNA extraction. Samples with sufficient DNA yield underwent PacBio HiFi genome sequencing on a PacBio Revio system targeting 30× mean coverage (18 trios and one quad; 58 samples). A subset of long-read-sequenced families also received proband-only SR-GS (11 probands) on a NovaSeq 6000 targeting 50× mean coverage. One trio with insufficient DNA yield underwent Illumina SR-GS only. Secondary analysis for LR-GS incorporated assembly- and read-based approaches. SR-GS data were processed using the Illumina DRAGEN pipeline. All variants subsequently underwent tertiary analysis for classification and reporting.

variant segregating in family PC01, the *AARS2* 6p21.1 (44306618–44310699)x1 deletion in family PC02, and the partial *NLRP12* deletion in family PC04 were detected by PAV, PacBio structural variant (pbsv), and Sniffles2 callers and ranked highly by both SvAnna and Emedgene. The *ACOX1* inversion in PC03 was detected only by PAV and Sniffles2, demonstrating variation in inversion sensitivity across SV callers. In addition, the inversion was ranked highly by SvAnna; inversion analysis is not currently supported by the Emedgene software. Lastly,

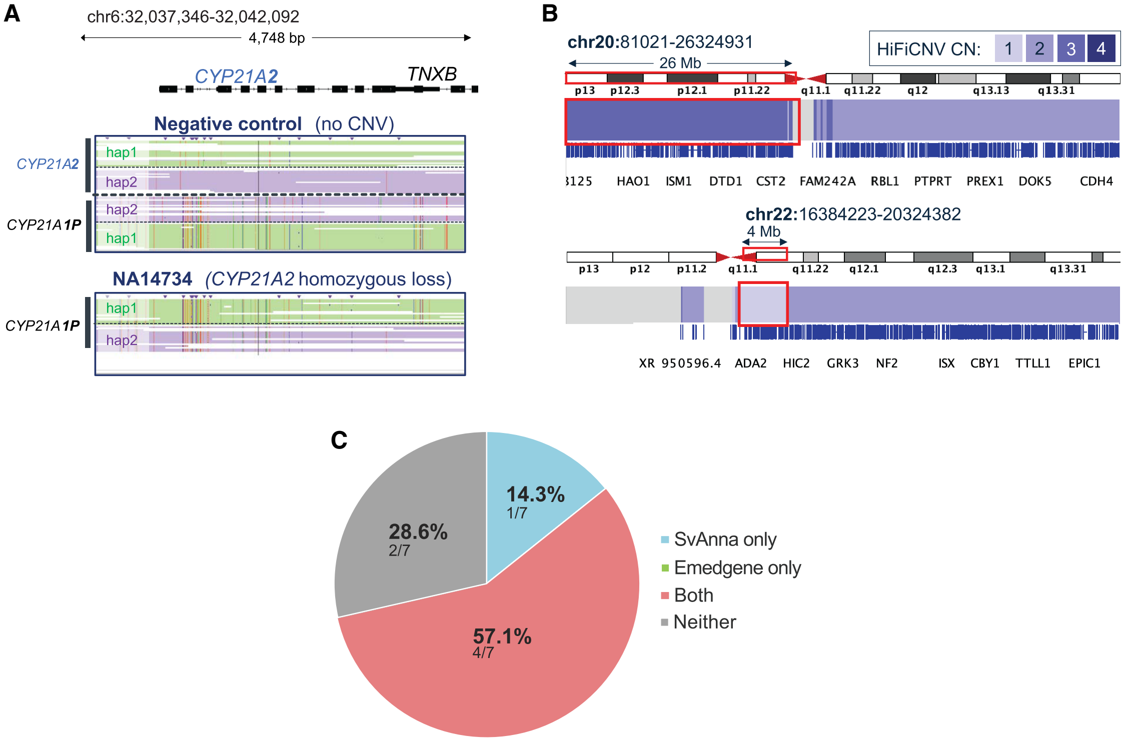

Figure 2. Evaluating LR-GS pipeline SV sensitivity

(A) IGV of phased re-aligned BAMs from Paraphase at the *CYP21A2* locus in NA14734 compared to a control subject without CNVs affecting the locus. No reads with matching haplotypes to the *CYP21A2* paralog are observed, signifying homozygous loss.
(B) IGV of HiFiCNV copy-number BEDGRAPHs, colored by copy number, capturing the translocation event involving the copy gain of the chr20 p arm and copy loss of a 4 Mb region on the chr22 q arm.
(C) Pie chart summary of tertiary analysis approaches of positive SV controls. Of the 7 causal variants, 2 were not prioritized by either software (gray), 4 were prioritized by both (red), and 1 inversion was prioritized by SvAnna only (blue).

the *STARD7* tandem repeat expansion segregating in family PC05 was detected fully in all family members with the PacBio Tandem Repeat Genotyping Tool (TRGT), as an insertion call with pbsv in two affected siblings (PC05P-1 and PC05P-2), and as an insertion call with Sniffles2 in one affected cousin (PC05C). The pbsv insertion call in individual PC05P-1 was present in one user-defined filter but was not labeled as a candidate by Emedgene, highlighting another area for tertiary analysis improvement. In summary, Emedgene and/or SvAnna were able to prioritize causal variants in 5/7 (71.4%) positive control subjects, with 2/7 (28.6%) requiring manual review of specialty caller outputs (Figure 2C).

Having established SV detection performance, we next evaluated the diagnostic yield of SVs in our LR-GS rare disease cohort. To optimize SV prioritization in the study cohort where causal variants are unknown, SVs with <80% reciprocal overlap with an allele frequency ≥0.01 in the following LR- and SR-GS population databases were first filtered out: Human Genome Structural Variation Consortium (HGSVC) v.2 and v.3, Consortium of Long Read Sequencing (CoLoRS), GA4K, Trans-Omics for Precision Medicine (TOPMed), Genome Aggregation Database (gnomAD), 1000 Genomes, and the National Human Genome Research Institute Centers for Common Disease Genomics (NHGRI-CCDG) program.[4,10,17–20] Population filtering reduces the SV load for tertiary analysis by 66.2%–88.9%, from an average per individual of 27,076–54,261 SVs to 3,004–14,317 SVs, depending on the caller (Figure 3A). As the first filtering step in our workflow, the relatively higher SV counts for pbsv likely reflect more false positives, particularly in low-complexity repetitive regions, which are partially mitigated by Sniffles2's coverage-adaptive, repeat-aware filtering and PAV's assembly-based breakpoint resolution. Downstream tertiary quality filtering combined with manual review further refined the candidate SV set. For SvAnna, a prioritization threshold of psv ≥ 2 was applied, aligning the number of candidates more closely with those from Emedgene (Figure 3B; supplemental material and methods). A total of 386 and 321 candidate SVs were prioritized by Emedgene and SvAnna, respectively, across all 19 LR-GS cases, with an average of 20 Emedgene and 17 SvAnna SV candidates per individual (Figure 3B). Of these, a total of 159 candidates were prioritized by both tools, with an average of 8 candidates per individual (Figure 3B). Manual inspection of aligned sequence reads in the Integrative Genomics Viewer (IGV) revealed that a substantial number of candidate SVs were technical artifacts, either entirely absent or with insufficient, ambiguous evidence supporting the SV. A high proportion of SvAnna-only (124/162, 76.5%) and Emedgene-SvAnna-shared (135/159, 84.9%)

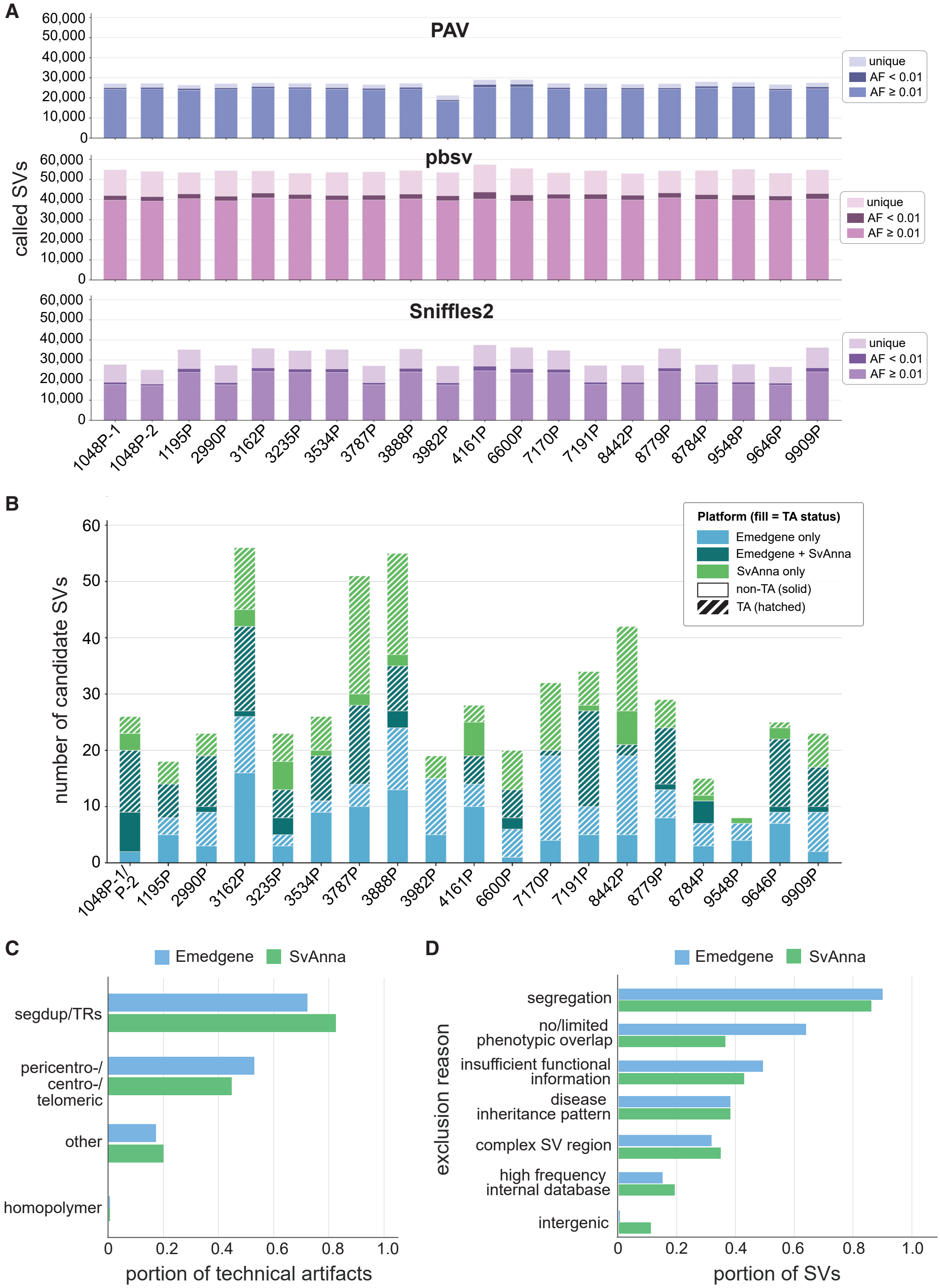

Figure 3. Structural variant filtering and prioritization in the LR-GS rare disease cohort

(A) Plot showing the portion of called SVs per proband ($n = 20$) by LR SV caller before additional filtering with the following allele frequency (AF) in population databases: ≥0.01, <0.01, and 0. Frequencies were determined by SVAFotate[17] against the following LR- and SR-GS population databases: HGSVC2/3, CoLoRSdb, GA4K, TOPMed, gnomAD, 1000 Genomes, and NHGRI-CCDG.[4,10,17–21]
(B) Plot showing the number of candidate SVs in Emedgene only (blue), SvAnna only (green, psv ≥ 2), or both platforms (teal) per proband ($n = 20$). Note: 1048P-1 and 1048P-2 were processed together in the same family and thus share the same candidates; only one bar is shown. Solid fill depicts true SVs, whereas hatched fill depicts the portion of SVs that were deemed technical artifacts.
(C) Plot showing the portion of technical artifact calls by underlying reason(s) for all probands ($n = 20$).
(D) Plot showing the portion of SVs across all probands ($n = 20$) that met different exclusion criteria. Emedgene is in blue, and SvAnna is in green.

candidate SVs were determined to be technical artifacts compared to roughly half of the Emedgene-only candidates (109/227, 48%) (Figure 3B). Technical artifact calls primarily arose from genomic regions containing segmental duplications, tandem repeats, and/or other repetitive loci, including homopolymer stretches, centromeres, pericentromeres, and telomeres (Figure 3C). For remaining candidates, SVs were most often excluded due to segregation of the variant in unaffected family members (87%–91%) and/or no or limited phenotypic overlap with the disease-associated gene(s) affected by the SV (37%–65%) (Figure 3D). In addition, several SVs affected loci with insufficient functional information (e.g., regulatory or intronic variants; 39%) or that fell within a genomic region populated with other SVs that are common, overlapping, or nearby in LR-GS population databases (32%–35%) (Figure 3D). A few SVs affected intergenic regions of unknown functional importance (1%–11%) (Figure 3D). Lastly, some SVs were common within the study cohort (15%–19%), underscoring the need for continual expansion of LR-GS population databases (Figure 3D).

Despite rigorous optimization and benchmarking of SV detection, diagnostic findings in our cohort were exclusively small variants (SNVs and indels) that were identifiable with SR-GS. Research use only (RUO) findings were shared with the medical team for 14/20 (70%) of families: 13/20 (65%) had small variant findings and 1/20 (5%) had SV findings (Table S4). Upon review of these candidate variants with the medical team, half were deemed inconclusive, resulting in a total of 7/20 (35%) families with potential diagnostic findings (Tables 2 and S4; Figure S2). The inconclusiveness of variants was attributed to factors such as mode of disease inheritance, uncertain population frequency data, limited phenotypic overlap, and insufficient molecular evidence. For instance, individuals 9909P, 3787P, and 7191P harbor a single inherited pathogenic or likely pathogenic variant for autosomal-recessive conditions with strong phenotypic match; however, systematic evaluation for additional variants in *trans*—including small variants and SVs—did not reveal a second pathogenic allele. Individual 4161P harbors a *de novo* frameshift variant in a GUS—*PTPDC1*—with several nearby or downstream predicted loss-of-function (LoF) variants observed in the general population (gnomAD v.4).[22] A hemizygous variant in the 3′ UTR of *FGF13* was identified in 1195P; while absent from gnomAD v.4[22] and phenotypically consistent with *FGF13*-related X-linked intellectual developmental disorder 110 (MIM: 301095), this variant lacks evidence of functional impact. Individual 2990P is homozygous for a ∼50 kb deletion within a segmental duplication on 8p23.1 spanning genes *ZNF705D*, *FAM66D*, *USP17L2*, and *USP17L7*, which lies adjacent to a region with no coverage in all analyzed individuals, most likely reflecting a rare or false duplication in the reference assembly (Figure S3; Table S4). While all genes within the deletion interval are GUSs, the *FAM66D* long non-coding RNA (lncRNA) is highly expressed in cortical neurons,[23] and its dysregulation has been implicated in Dravet syndrome[24]—biology and clinical features consistent with 2990P (Table S1; see the supplemental note). However, this deletion is part of a larger ∼247 kb deletion that is present in the heterozygous state at an allele frequency of ∼4.6% (6/130 alleles) in the newest release of HGSVC (v.3),[20] suggesting that it may be too common to cause disease. Lastly, compound heterozygous variants in a *VPS26C*, including a possible downstream regulatory variant and a missense variant, were prioritized for individual 8784P due to a recent report implicating bi-allelic *VPS26C* variants in two related individuals with a neurodevelopmental disorder (NDD)[25] with phenotypic overlap to 8784P (Table S1; see the supplemental note); however, preliminary RNA sequencing (RNA-seq) findings from participant peripheral blood mononuclear cells (PBMCs) suggest that expression of *VPS26C* is unaffected (Figures S4 and S5A).

For the remaining eight individuals from seven families with clinically relevant findings, all nine variants were clinically confirmed by the source testing laboratory using clinical SR data or Sanger sequencing, with results returned to the families (Tables 2 and S4). Of these eight individuals, research SR-GS was performed for four individuals and successfully detected all five variants (Table 2), demonstrating that LR-GS was not required for their detection. Reported variants were stratified into the following two diagnostic categories based on existing classification schemes[28]: diagnostic variant (DV) and variant of uncertain diagnostic significance (VUDS) (Table 2).

DVs were reported for two families. In family 8442, 8442P is heterozygous for a *de novo* likely pathogenic missense variant, c.1273G>A (GenBank: NM_002577.4) (p.Asp425Asn), in *PAK2* (Figures 4A and 4B). Pathogenic variants in *PAK2* cause Knobloch syndrome 2 (MIM: 618458), an autosomal-dominant disorder that strongly overlaps 8442P's clinical phenotypes of global developmental delay, pyloric stenosis, and retinal detachment[32] (Tables 2, S1, and S4; see the supplemental note). Following clinical confirmation and result sharing with the family, this molecular diagnosis prompted referral for reproductive genetic counseling, which informed family planning decisions. This result also provided diagnostic closure for the family, aiding understanding and acceptance of disease etiology and facilitating more focused care management.

In family 2598, who received SR-GS only, a pathogenic *de novo* variant was discovered in 2598P in *DDX17*, c.1077G>A (GenBank: NM_006386.5) (p.Trp359Ter) (Figures 4A, 4B, and S2A; Tables 2 and S4). *DDX17*, an RNA helicase, is highly intolerant to heterozygous predicted LoF variants in the general population (probability of LoF Intolerance [pLI] = 1, LoF observed/expected upper bound fraction [LOEUF] = 0.25; gnomAD v.4),[22] suggestive of haploinsufficiency. Consistent with this, a recent report implicated *de novo* missense and predicted LoF

Table 2. Summary of clinically confirmed diagnostic variants in present study (all small variants)

Individual	Gene	Variant type	Variant	Classification (ACMG codes)	Inheritance, zygosity	OMIM	Detection method: Research study[a]	Detection method: Clinical test site[b]	Diagnostic significance
8442P	*PAK2*	missense	c.1273G>A (GenBank: NM_002577.4) (p.Asp425Asn) (GenBank: NP_002568.2)	likely pathogenic (PM2_Mod, PS2_Strong)	*de novo*, heterozygous	AD - Knobloch syndrome 2 (MIM: 618458)	LR-GS, SR-GS	ES reanalysis	DV
2598P	*DDX17*	nonsense	c.1077G>A (GenBank: NM_006386.5) (p.Trp359*) (GenBank: NP_006377.2)	pathogenic (PVS1_VS, PS2_Strong, PM2_Mod)	*de novo*, heterozygous	AD - DDX17 neurodevelopmental disorder[26]	LR-GS, SR-GS	Sanger sequencing	DV
1048P-1/ 1048P-2	*DOCK4*	missense	c.2498T>A (GenBank: NM_014705.4) (p.Val833Asp) (GenBank: NP_055520.3)	VUS (PM2_Mod, PP1_Supp, PP3_Supp)	maternal, heterozygous	AD - DOCK4-associated neurodevelopmental delay and microcephaly[27]	LR-GS	ES reanalysis	VUDS
7170P	*SOCS1*	missense	c.340G>A (GenBank: NM_003745.2) (p.Ala114Thr) (GenBank: NP_003736.1)	VUS (PM2_Mod)	maternal, heterozygous	AD - autoinflammatory syndrome, familial, with or without immunodeficiency (MIM: 619375)	LR-GS, SR-GS	ES reanalysis	VUDS
3982P	*GEMIN5*	missense	c.863C>G (GenBank: NM_015465.5) (p.Thr288Arg) (GenBank: NP_056280.2)	VUS (PM2_Mod)	maternal, heterozygous	AR - neurodevelopmental disorder with cerebellar atrophy and motor dysfunction (MIM: 619333)	LR-GS	ES reanalysis	VUDS
		missense	c.1291G>A (GenBank: NM_015465.5) (p.Ala431Thr) (GenBank: NP_056280.2)	VUS (PM2_Mod)	paternal, heterozygous	AR - neurodevelopmental disorder with cerebellar atrophy and motor dysfunction (MIM: 619333)	LR-GS	ES reanalysis	VUDS
8779P	*TRAK1*	missense	c.757G>A (GenBank: NM_001042646.3) (p.Val253Met) (GenBank: NP_001036111.1)	VUS (PM2_Mod)	paternal, heterozygous	AR - developmental and epileptic encephalopathy 68 (MIM: 618201)	LR-GS, SR-GS	ES reanalysis; Sanger sequencing	VUDS
		intronic	c.2066+915G>C (GenBank: NM_001042646.3)	VUS (PM2_Mod)	maternal, heterozygous	AR - developmental and epileptic encephalopathy 68 (MIM: 618201)	LR-GS, SR-GS	Sanger sequencing	VUDS
9548P	*TIMM23*	missense	c.563C>A (GenBank: NM_006327.4) (p.Thr188Asn) (GenBank: NP_006318.1)	GUS - N/A	biparental, homozygous	N/A	LR-GS	ES reanalysis; Sanger sequencing	VUDS

AD, autosomal dominant; AR, autosomal recessive; VUS, variant of uncertain significance; GUS, gene of uncertain significance; Mod, moderate; VS, very strong; Supp, supporting; DV, diagnostic variant; VUDS, variant of uncertain diagnostic significance; N/A, not applicable.

[a]Method(s) that independently detected the variant in the present research study.

[b]Method performed during clinical confirmation by the original clinical laboratory for the proband.

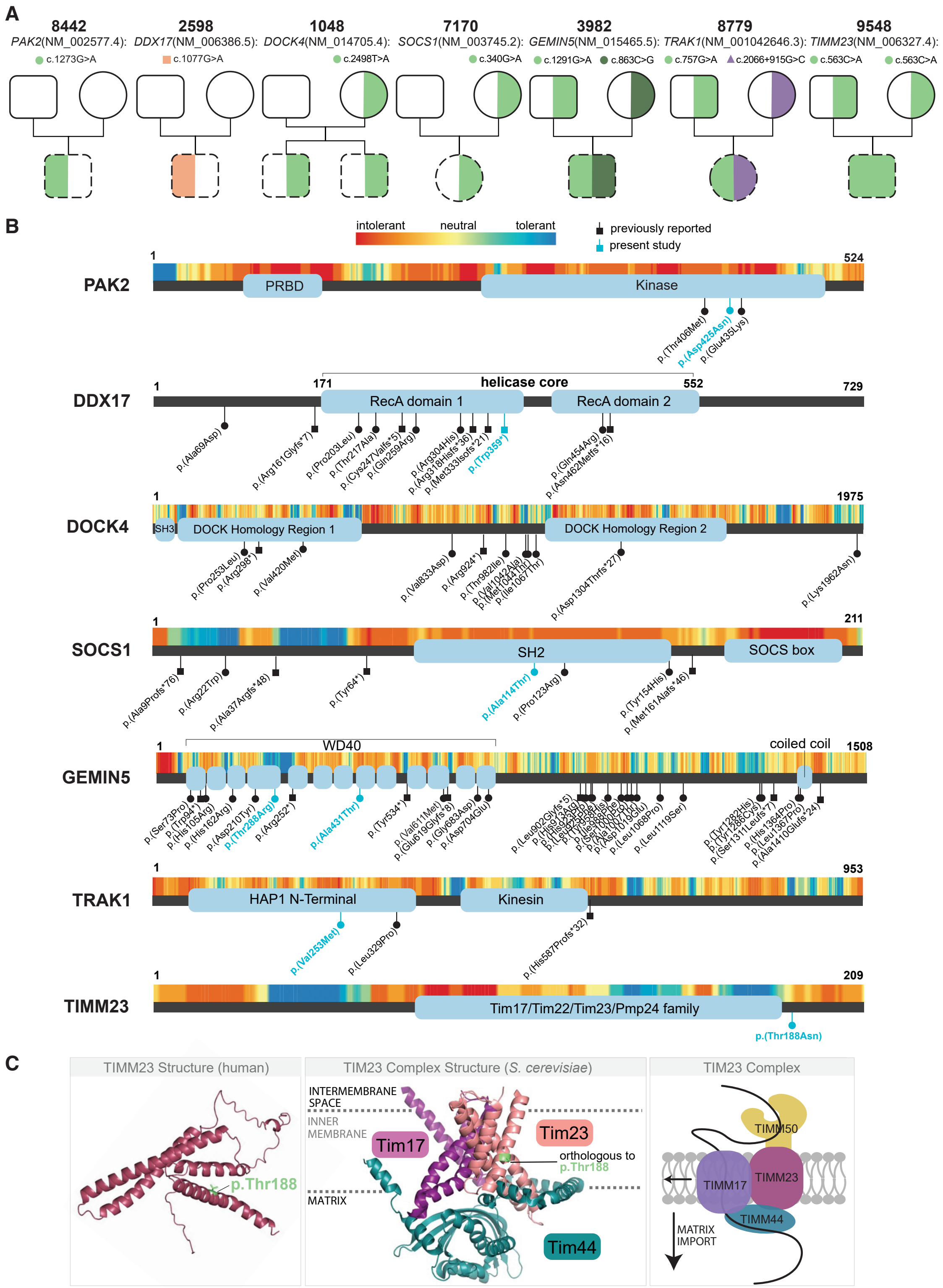

Figure 4. . Individuals with clinically confirmed diagnostic findings

(A) Family pedigrees. Squares and circles represent assigned male and female at birth, respectively. Missense, nonsense, and potential regulatory variants are represented by green, orange, and purple shading, respectively. Heterozygous is indicated by half shading and homozygous by full shading. Affected individuals are represented with a dashed black outline.

(B) Linear protein map of PAK2, DDX17, DOCK4, SOCS1, GEMIN5, TRAK1, and TIMM23 with annotated protein domains (UniProt.org) and rare disease-associated variants mapped. Variants detected in the present study are shown in blue; previously reported variants

(legend continued on next page)

variants in *DDX17* in individuals with NDD features, several of which overlap 2598P, including global developmental delay, speech difficulties, and hypotonia (Table S1; see the supplemental note).[26] *DDX17* c.1077G>A (p.Trp359Ter) lies within the RecA domain 1 of DDX17, wherein the majority of reported variants cluster, and upstream of one reported frameshift variant (Figure 4B).[26] While previously published *DDX17* disease variants are heterozygous, the variant allele fraction (VAF) of *DDX17* c.1077G>A in 2598P in our SR-GS data was 0.31, and clinical confirmation performed by the original clinical testing site was consistent with mosaicism. Taken together, additional clinical cases are needed to define the core phenotypic spectrum of this recently characterized disorder.

VUDSs were reported in five families. Similarly affected siblings 1048P-1 and 1048P-2 harbor a maternally inherited missense variant, c.2498T>A (GenBank: NM_014705.4) (p.Val833Asp), in *DOCK4* (Figures 4A, 4B, and S2B; Tables 2 and S4). Pathogenic variants in *DOCK4* have been implicated in autosomal-dominant *DOCK4*-associated neurodevelopmental delay and microcephaly with reports of incomplete penetrance.[27] Although the disorder shows phenotypic overlap with each sibling's clinical features (Table S1; see the supplemental note), c.2498T>A (GenBank: NM_014705.4) is a VUDS because it lacks supporting functional evidence. A maternally inherited missense variant was also detected in individual 7170P, c.340G>A (GenBank: NM_003745.2) (p.Ala114Thr), in *SOCS1* (Figures 4A, 4B, and S2C; Tables 2 and S4). *SOCS1* is associated with autosomal-dominant familial autoinflammatory syndrome with or without immunodeficiency (MIM: 619375), in which incomplete penetrance and variable expressivity are common, possibly underlying the phenotypic variability observed in affected maternal family members of 7170P (Figure 3D; Table S1; see the supplemental note).

Individual 3982P is compound heterozygous for two missense VUDSs that lie within tryptophan-aspartic acid (WD) repeats of *GEMIN5* and affect highly conserved residues: c.863C>G (GenBank: NM_015465.5) (p.Thr288Arg) and c.1291G>A (GenBank: NM_015465.5) (p.Ala431Thr) (Figures 4A, 4B, and S2D; Tables 2 and S4). GEMIN5 p.Ala431Thr affects a residue intolerant to missense variation (non-synonymous to synonymous substitution rate (dN/dS) = 0.29, MetaDome[29]; Figure 4B), and computational tools predict a deleterious effect of GEMIN5 p.Thr288Arg (Table S4). *GEMIN5* is implicated in autosomal-recessive NDD with cerebellar atrophy and motor dysfunction (NEDCAM; MIM: 619333), overlapping intellectual disability, seizures, hypotonia, absent speech, and motor delay in 3982P (Table S1; see the supplemental note). The majority of reported variants in *GEMIN5*-associated NEDCAM are missense (22/30, 73.3%), 6 of which also lie within the WD40 domain near GEMIN5 p.Ala431Thr and p.Thr288Arg (Figure 3B).[33]

Similarly, individual 8779P is compound heterozygous for VUDSs in *TRAK1*: c.757G>A (GenBank: NM_001042646.3) (p.Val253Met) and c.2066+915G>C (GenBank: NM_001042646.3) (Figures 4A, 4B, and S2E; Tables 2 and S4). *TRAK1* is associated with autosomal-recessive developmental and epileptic encephalopathy 68 (DEE68; MIM: 618201), which overlaps part of 8779P's described phenotype: seizure, global developmental delay, intellectual disability, motor delay, absent speech, apnea, and hypotonia (Table S1; see the supplemental note). TRAK1 p.Val253Met lies within the HAP1 N-terminal domain of the protein, affecting a residue intolerant to missense variation (dN/dS = 0.26, MetaDome[29]) nearby a previously reported missense variant in DEE68[34] (Figure 4B). The c.2066+915G>C (GenBank: NM_001042646.3) variant lies within intron 15 of 15 and affects a region with a proximal enhancer-like signature (ENCODE Candidate *Cis*-regulatory Elements database).[35] This variant also results in a missense change in an alternative transcript of *TRAK1* (c.2079G>C [GenBank: NM_001349247.2] [p.Glu693Asp). *TRAK1* isoform GenBank: NM_001349247 (ENST00000327628.9) demonstrates low or absent expression in adult brain or developing cortex tissue, respectively, with expression observed in adult skin and heart tissue (GTEx v.8, ENCODE v.4, and PacBio Iso-Seq; Figure S6).[36–38] Further molecular investigation of the functional consequence of this variant in both transcript contexts, as well as of TRAK1 p.Val253Met, is needed to substantiate their clinical significance in 8779P.

The final individual with a reported VUDS lies within a GUS. Individual 9548P is homozygous for *TIMM23* missense variant c.563C>A (GenBank: NM_006327.4) (p.Thr188Asn) (Figures 4 and S2F; Tables 2 and S4). While *TIMM23* has not been implicated in human disease to date, it demonstrates slight intolerance to missense variation gene wide (Z = 1.51, LOEUF = 0.84; gnomAD v.4[22]). The c.563C>A (GenBank: NM_006327.4) variant is present in 4/1,614,174 alleles (2.5×10^{-6}) in the heterozygous state only in gnomAD v.4[22] and affects a residue (p.Thr188) intolerant to missense variants (dN/dS = 0.59, MetaDome[29]) (Figure 4B; Table S4). Computational tools predict a deleterious effect of TIMM23 p.Thr188Asn on the protein (Table S4), with a deep learning prediction of a slightly destabilizing change in Gibbs free energy ($\Delta\Delta G$) of −0.2 kcal/mol (DDMut[39]). TIMM23 is a key component of the translocase of the inner membrane

are in black. Above the protein map is a missense intolerance heatmap by residue (intolerant in red to tolerant in blue) based on dN/dS ratios (MetaDome[29]).
(C) Human TIMM23 protein structure (AlphaFold[30] AF-O14925 F1; PyMOL) with p.Thr188 residue mapped (left). Yeast TIM23 complex cryoelectron microscopy (cryo-EM) structure (PDB: 8E1M[31]; PyMOL) with the orthologous residue to p.Thr188 mapped (middle) and a TIM23 complex cartoon (right).

(TIM23) complex, which mediates the import of preproteins to the inner mitochondrial membrane and mitochondrial matrix.[31,40] While *TIMM23* has not been implicated in disease to date, pathogenic variants in other TIM23 complex subunits cause developmental disorders with partial phenotypic overlap to 9548P, including *TIMM50* in 3-methylglutaconic aciduria type IX (MIM: 617698), *DNAJC19* in 3-methylglutaconic aciduria type V (MIM: 610198), and *HSPA9* in EVEN-plus (epiphyseal, vertebral, ear, nose dysplasia plus associated findings) syndrome (MIM: 616854) (Table S1; see the supplemental note). Preliminary investigation of RNA expression (see supplemental material and methods) in PBMCs obtained from family 9548 does not suggest a change in expression of the TIM23 complex members (Figure S5B), nor of genes encoding mitochondria-associated proteins (data not shown). Immune signaling genes appear negatively enriched in 9548P PBMCs (Figures S5C–S5E), a finding consistent with previous transcriptomic evidence showing immune suppression via downregulation of adaptive immunity in PBMCs derived from individuals with mitochondrial disease[41] as well as immunodeficiencies observed in individuals with mitochondrial disease.[42] However, the lack of additional affected individuals to serve as biological replicates and the limited *TIMM23* expression in PBMCs warrant a more robust investigation to implicate *TIMM23* in human disease and evaluate the role of c.563C>A (GenBank: NM_006327.4) (p.Thr188Asn) in the clinical presentation of 9548P. Taken together, all described VUDSs in this study necessitate further functional investigation to determine their pathogenicity in the respective disorder.

In summary, diagnostic yield ranged from 10% for DV findings to 25% for VUDS findings. While these results are consistent with recent reports in larger LR-GS rare disease cohorts,[4,6,9] the improved diagnostic yield in this small cohort can be attributed to genome reanalysis without increased detection from LR-GS. These results emphasize the need for periodic reanalysis, as new evidence can shift variant interpretation, exemplified by reclassifications of *PAK2* and *DDX17* variants in this study. In contrast, the reclassification of previously excluded variants as VUDSs in our study likely stems from inter-laboratory variability in clinical variant interpretation rather than emerging data. The absence of diagnostic SV findings, despite demonstrated analytic sensitivity, indicates that SVs were not a major contributor to disease in this small cohort. Several factors may have contributed to the lack of LR-specific findings in addition to low sample numbers, including ambiguity of SV interpretation. For instance, a proportion of candidate SVs that affected non-coding or intergenic regions were excluded in the present study given the lack of defined regulatory functions of affected regions. In addition, clinical interpretation strongly relies on allele frequency data from the general population to assess candidacy; however, LR SV population datasets are still incomplete compared to small-variant databases. Moreover, clinical SV databases such as ClinVar,[43] ClinGen,[44] and DECIPHER[45] are sparsely curated. In some samples in the present study, SVs mapped to segmental duplication regions with reference bias and/or overlapped other similar or complex SVs in HGSVC[10,20] data, requiring the use of arbitrary cutoffs to define SV identity for assessing allele frequency. In several samples, SVs that span multiple genes, including known disease-associated genes, were excluded from clinical consideration if segregation did not follow the expected inheritance pattern of the disease gene, reflecting a rigid interpretation based on monogenic Mendelian disease frameworks.

SVs may exert pathway-level, context-dependent, partial, or modifying effects, leading to incomplete penetrance and/or variable expressivity of disease—factors often underrecognized in clinical pipelines.[46–48] Both common and rare SVs (including non-coding SVs lacking regulatory annotations) have been shown to affect the expression of multiple nearby genes, driving strong and/or pleiotropic effects on phenotypic diversity.[49] Long-range regulation further complicates interpretation, as enhancers can act over large genomic distances, typically in *cis*[50,51] but sometimes in *trans* via interchromosomal enhancer-gene interactions.[52,53] Recurrent copy-number variant (CNV) syndromes often demonstrate incomplete penetrance,[54,55] with recent analyses suggesting penetrance of just 1%–10%.[56] Additionally, variable expressivity, dosage sensitivity, and modifier effects from other genomic variants contribute to clinical severity in recurrent CNV syndromes, notably for 1q21.1 deletions,[57] 16p11.2 duplications,[58] and Xq28 duplications.[59] Collectively, these findings highlight the need for SV interpretation frameworks that move beyond single-gene, strong effect models to better incorporate regional effects, regulatory architecture, modifiers, and incomplete penetrance.[48] In addition to genomic SVs, investigation into the contribution of non-coding variants and differential methylation to disease phenotypes in this cohort is currently ongoing, which may uncover missed molecular etiologies.

Nevertheless, evaluation of the LR analysis pipeline's sensitivity using rare disease samples with known SVs highlighted the necessity of a multi-pronged approach due to the variable performance characteristics of read-based, assembly-based, and specialty SV callers across variant classes. Tertiary analysis demonstrated comparable performance between SvAnna and Emedgene for multiple SV types; however, it revealed a notable limitation of Emedgene in the annotation of inversion events. An abundance of small variants and SVs inherited from unaffected parents were prioritized by both SvAnna and Emedgene, highlighting the significance of trio-based analysis for effective candidate variant interpretation in rare disease. Likewise, the incorporation of population frequency filtering in LR-GS databases greatly refined SV prioritization by SvAnna and Emedgene, primarily by excluding common SVs not captured in SR datasets. Taken together, an integrative and systematic analysis is essential for maximizing the

clinical value of LR-GS data. Although LR-GS did not provide additional diagnostic insight in this rare disease cohort, the findings underscore the importance of routine reanalysis to improve diagnostic yield.

Data and code availability

All sequencing data used to support the findings of this study are restricted by the IRB committees at the CC and JGM to protect participant privacy. For participants who have consented to future data use, sequencing data have been deposited to the European Genome-phenome Archive (EGA). The accession number for the sequence data reported in this paper is EGA: EGAD50000002109 (Table S2). Variant classification data used to support the findings of this study have been deposited in the ClinVar repository for individual 8442C (ID 3081741). The LR-GS analysis pipeline has been converted to Nextflow and adapted for public release; the workflow is publicly available at https://github.com/TheJacksonLaboratory/jax-apml-lrs.

Acknowledgments

We thank all families for their participation in this work. We thank Charles Lee, Juan C. Salazar, Christine R. Beck, Alyx Vogle, and Kunal Sanghavi for their intellectual contributions. We thank Tomi Pastinen and Emily Farrow at Children's Mercy Hospital Kansas City for granting GA4K authorized data access. All sequencing was performed by Genome Technologies and the Advanced Precision Medicine Laboratory at the Jackson Laboratory for Genomic Medicine. This work was funded by Connecticut Children's Research Institute and the Jackson Laboratory.

Author contributions

Manuscript and manuscript editing, E.A.W., M.A.K., G.E.R., M.D.A., A.P.M., L.K., and P.N.R.; figures, E.A.W.; participant recruitment and sample collection, A.P.M., L.K., M.P., and C.K.; sample extraction and sequencing, P.V. and R.D.G.; data analysis, E.A.W., M.A.K., G.E.R., and P.A.A.; IRB management, M.D.A., A.P.M., and E.J.C.

Declaration of interests

The authors declare no competing interests.

Supplemental information

Supplemental information can be found online at https://doi.org/10.1016/j.xhgg.2026.100620.

Web resources

AlphaMissense, https://alphamissense.hegelab.org/
ClinVar, https://www.ncbi.nlm.nih.gov/clinvar/
Combined Annotation Dependent Depletion (CADD), https://cadd.gs.washington.edu
dbGaP, https://dbgap.ncbi.nlm.nih.gov/home/
DDMut, https://biosig.lab.uq.edu.au/ddmut/
GenBank, https://www.ncbi.nlm.nih.gov/genbank/
MetaDome, stuart.radboudumc.nl/metadome
MetaScape, https://metascape.org/
Online Mendelian Inheritance in Man (OMIM), http://www.omim.org
PDB, https://www.rcsb.org
PolyPhen-2, http://genetics.bwh.harvard.edu/pph2/
SpliceAI, https://spliceailookup.broadinstitute.org/
UCSC Genome Browser, https://genome.ucsc.edu/
UniProt, https://www.uniprot.org/

Received: December 22, 2025
Accepted: April 23, 2026

References

1. 1000 Genomes Project Consortium, Auton, A., Brooks, L.D., Durbin, R.M., Garrison, E.P., Kang, H.M., Korbel, J.O., Marchini, J.L., McCarthy, S., McVean, G.A., and Abecasis, G.R. (2015). A global reference for human genetic variation. Nature *526*, 68–74. https://doi.org/10.1038/nature15393.
2. Wojcik, M.H., Lemire, G., Berger, E., Zaki, M.S., Wissmann, M., Win, W., White, S.M., Weisburd, B., Wieczorek, D., Waddell, L.B., et al. (2024). Genome Sequencing for Diagnosing Rare Diseases. N. Engl. J. Med. *390*, 1985–1997. https://doi.org/10.1056/NEJMoa2314761.
3. Eisfeldt, J., Ek, M., Nordenskjöld, M., and Lindstrand, A. (2025). Toward clinical long-read genome sequencing for rare diseases. Nat. Genet. *57*, 1334–1343. https://doi.org/10.1038/s41588-025-02160-y.
4. Cohen, A.S.A., Farrow, E.G., Abdelmoity, A.T., Alaimo, J.T., Amudhavalli, S.M., Anderson, J.T., Bansal, L., Bartik, L., Baybayan, P., Belden, B., et al. (2022). Genomic answers for children: Dynamic analyses of >1000 pediatric rare disease genomes. Genet. Med. *24*, 1336–1348. https://doi.org/10.1016/j.gim.2022.02.007.
5. Mortazavi, M., Guevara, J., Diaz, J., Tran, S., Ziaei Jam, H., Reeves, C., Batalov, S., Jepsen, K., Bainbridge, M., Besterman, A.D., et al. (2026). Long-read genome sequencing improves detection and functional interpretation of structural and repeat variants in autism. Cell Genom. 101186.
6. Hiatt, S.M., Lawlor, J.M.J., Handley, L.H., Latner, D.R., Bonnstetter, Z.T., Finnila, C.R., Thompson, M.L., Boston, L.B., Williams, M., Rodriguez Nunez, I., et al. (2024). Long-read genome sequencing and variant reanalysis increase diagnostic yield in neurodevelopmental disorders. Genome Res. *34*, 1747–1762. https://doi.org/10.1101/gr.279227.124.
7. Sui, Y., Lin, J., Noyes, M.D., Kwon, Y., Wong, I., Koundinya, N., Harvey, W.T., Wu, M., Hoekzema, K., Munson, K.M., et al. (2026). Using the linear references from the pangenome to discover missing autism variants. Nat. Commun. *17*, 1681. https://doi.org/10.1038/s41467-026-68378-4.
8. Groza, C., Schwendinger-Schreck, C., Cheung, W.A., Farrow, E.G., Thiffault, I., Lake, J., Rizzo, W.B., Evrony, G., Curran, T., Bourque, G., and Pastinen, T. (2024). Pangenome graphs improve the analysis of structural variants in rare genetic diseases. Nat. Commun. *15*, 657. https://doi.org/10.1038/s41467-024-44980-2.
9. Thiffault, I., Farrow, E., Barrett, C., Scott, M., Ross, A., Means, J.C., Cheung, W.A., Johnson, A.F., Koseva, B., McLennan, R., et al. (2025). Clinical Long-Read Sequencing Test for Genetic Disease Diagnosis. JAMA Pediatr. *179*, 1355–1357. https://doi.org/10.1001/jamapediatrics.2025.3320.
10. Ebert, P., Audano, P.A., Zhu, Q., Rodriguez-Martin, B., Porubsky, D., Bonder, M.J., Sulovari, A., Ebler, J., Zhou, W., Serra

Mari, R., et al. (2021). Haplotype-resolved diverse human genomes and integrated analysis of structural variation. Science *372*, eabf7117. https://doi.org/10.1126/science.abf7117.
11. Cheng, H., Concepcion, G.T., Feng, X., Zhang, H., and Li, H. (2021). Haplotype-resolved de novo assembly using phased assembly graphs with hifiasm. Nat. Methods *18*, 170–175. https://doi.org/10.1038/s41592-020-01056-5.
12. Poplin, R., Chang, P.C., Alexander, D., Schwartz, S., Colthurst, T., Ku, A., Newburger, D., Dijamco, J., Nguyen, N., Afshar, P.T., et al. (2018). A universal SNP and small-indel variant caller using deep neural networks. Nat. Biotechnol. *36*, 983–987. https://doi.org/10.1038/nbt.4235.
13. Martin, M., Ebert, P., and Marschall, T. (2023). Read-Based Phasing and Analysis of Phased Variants with WhatsHap. Methods Mol. Biol. *2590*, 127–138. https://doi.org/10.1007/978-1-0716-2819-5_8.
14. Smolka, M., Paulin, L.F., Grochowski, C.M., Horner, D.W., Mahmoud, M., Behera, S., Kalef-Ezra, E., Gandhi, M., Hong, K., Pehlivan, D., et al. (2024). Detection of mosaic and population-level structural variants with Sniffles2. Nat. Biotechnol. *42*, 1571–1580. https://doi.org/10.1038/s41587-023-02024-y.
15. Richards, S., Aziz, N., Bale, S., Bick, D., Das, S., Gastier-Foster, J., Grody, W.W., Hegde, M., Lyon, E., Spector, E., et al. (2015). Standards and guidelines for the interpretation of sequence variants: a joint consensus recommendation of the American College of Medical Genetics and Genomics and the Association for Molecular Pathology. Genet. Med. *17*, 405–424. https://doi.org/10.1038/gim.2015.30.
16. Danis, D., Jacobsen, J.O.B., Balachandran, P., Zhu, Q., Yilmaz, F., Reese, J., Haimel, M., Lyon, G.J., Helbig, I., Mungall, C.J., et al. (2022). SvAnna: efficient and accurate pathogenicity prediction of coding and regulatory structural variants in long-read genome sequencing. Genome Med. *14*, 44. https://doi.org/10.1186/s13073-022-01046-6.
17. Nicholas, T.J., Cormier, M.J., and Quinlan, A.R. (2022). Annotation of structural variants with reported allele frequencies and related metrics from multiple datasets using SVAFotate. BMC Bioinf. *23*, 490. https://doi.org/10.1186/s12859-022-05008-y.
18. Taliun, D., Harris, D.N., Kessler, M.D., Carlson, J., Szpiech, Z.A., Torres, R., Taliun, S.A.G., Corvelo, A., Gogarten, S.M., Kang, H.M., et al. (2021). Sequencing of 53,831 diverse genomes from the NHLBI TOPMed Program. Nature *590*, 290–299. https://doi.org/10.1038/s41586-021-03205-y.
19. Koenig, Z., Yohannes, M.T., Nkambule, L.L., Zhao, X., Goodrich, J.K., Kim, H.A., Wilson, M.W., Tiao, G., Hao, S.P., Sahakian, N., et al. (2024). A harmonized public resource of deeply sequenced diverse human genomes. Genome Res. *34*, 796–809. https://doi.org/10.1101/gr.278378.123.
20. Logsdon, G.A., Ebert, P., Audano, P.A., Loftus, M., Porubsky, D., Ebler, J., Yilmaz, F., Hallast, P., Prodanov, T., Yoo, D., et al. (2025). Complex genetic variation in nearly complete human genomes. Nature *644*, 430–441. https://doi.org/10.1038/s41586-025-09140-6.
21. Abel, H.J., Larson, D.E., Regier, A.A., Chiang, C., Das, I., Kanchi, K.L., Layer, R.M., Neale, B.M., Salerno, W.J., Reeves, C., et al. (2020). Mapping and characterization of structural variation in 17,795 human genomes. Nature *583*, 83–89. https://doi.org/10.1038/s41586-020-2371-0.
22. Chen, S., Francioli, L.C., Goodrich, J.K., Collins, R.L., Kanai, M., Wang, Q., Alföldi, J., Watts, N.A., Vittal, C., Gauthier, L.D., et al. (2024). A genomic mutational constraint map using variation in 76,156 human genomes. Nature *625*, 92–100. https://doi.org/10.1038/s41586-023-06045-0.
23. Velmeshev, D., Schirmer, L., Jung, D., Haeussler, M., Perez, Y., Mayer, S., Bhaduri, A., Goyal, N., Rowitch, D.H., and Kriegstein, A.R. (2019). Single-cell genomics identifies cell type-specific molecular changes in autism. Science *364*, 685–689. https://doi.org/10.1126/science.aav8130.
24. Schuster, J., Laan, L., Klar, J., Jin, Z., Huss, M., Korol, S., Noraddin, F.H., Sobol, M., Birnir, B., and Dahl, N. (2019). Transcriptomes of Dravet syndrome iPSC derived GABAergic cells reveal dysregulated pathways for chromatin remodeling and neurodevelopment. Neurobiol. Dis. *132*, 104583. https://doi.org/10.1016/j.nbd.2019.104583.
25. Beetz, C., Ameziane, N., Kdissa, A., Karageorgou, V., Bauer, P., Suleiman, J., Sutton, V.R., and El-Hattab, A.W. (2020). VPS26C homozygous nonsense variant in two cousins with neurodevelopmental deficits, growth failure, skeletal abnormalities, and distinctive facial features. Clin. Genet. *97*, 644–648. https://doi.org/10.1111/cge.13690.
26. Seaby, E.G., Godwin, A., Meyer-Dilhet, G., Clerc, V., Grand, X., Fletcher, T., Monteiro, L., Kerkhofs, M., Carelli, V., Palombo, F., et al. (2025). Monoallelic de novo variants in DDX17 cause a neurodevelopmental disorder. Brain *148*, 1155–1168. https://doi.org/10.1093/brain/awae320.
27. Herbst, C., Bothe, V., Wegler, M., Axer-Schaefer, S., Audebert-Bellanger, S., Gecz, J., Cogne, B., Feldman, H.B., Horn, A.H.C., Hurst, A.C.E., et al. (2024). Heterozygous loss-of-function variants in DOCK4 cause neurodevelopmental delay and microcephaly. Hum. Genet. *143*, 455–469. https://doi.org/10.1007/s00439-024-02655-4.
28. Salfati, E.L., Spencer, E.G., Topol, S.E., Muse, E.D., Rueda, M., Lucas, J.R., Wagner, G.N., Campman, S., Topol, E.J., and Torkamani, A. (2019). Re-analysis of whole-exome sequencing data uncovers novel diagnostic variants and improves molecular diagnostic yields for sudden death and idiopathic diseases. Genome Med. *11*, 83. https://doi.org/10.1186/s13073-019-0702-2.
29. Wiel, L., Baakman, C., Gilissen, D., Veltman, J.A., Vriend, G., and Gilissen, C. (2019). MetaDome: Pathogenicity analysis of genetic variants through aggregation of homologous human protein domains. Hum. Mutat. *40*, 1030–1038. https://doi.org/10.1002/humu.23798.
30. Jumper, J., Evans, R., Pritzel, A., Green, T., Figurnov, M., Ronneberger, O., Tunyasuvunakool, K., Bates, R., Žídek, A., Potapenko, A., et al. (2021). Highly accurate protein structure prediction with AlphaFold. Nature *596*, 583–589. https://doi.org/10.1038/s41586-021-03819-2.
31. Sim, S.I., Chen, Y., Lynch, D.L., Gumbart, J.C., and Park, E. (2023). Structural basis of mitochondrial protein import by the TIM23 complex. Nature *621*, 620–626. https://doi.org/10.1038/s41586-023-06239-6.
32. Werren, E.A., Kalsner, L., Ewald, J.M., Peracchio, M., King, C., Vats, P., Audano, P.A., Robinson, P.N., Adams, M.D., Kelly, M.A., and Matson, A.P. (2025). Phenotypic Expansion of Knobloch Syndrome Type 2 in an Individual With a De Novo PAK2 Variant. Am. J. Med. Genet. *197*, e64006. https://doi.org/10.1002/ajmg.a.64006.
33. Kour, S., Rajan, D.S., Fortuna, T.R., Anderson, E.N., Ward, C., Lee, Y., Lee, S., Shin, Y.B., Chae, J.H., Choi, M., et al. (2021). Loss of function mutations in GEMIN5 cause a neurodevelopmental disorder. Nat. Commun. *12*, 2558. https://doi.org/10.1038/s41467-021-22627-w.

34. Sagie, S., Lerman-Sagie, T., Maljevic, S., Yosovich, K., Detert, K., Chung, S.K., Rees, M.I., Lerche, H., and Lev, D. (2018). Expanding the phenotype of TRAK1 mutations: hyperekplexia and refractory status epilepticus. Brain *141*, e55. https://doi.org/10.1093/brain/awy129.
35. ENCODE Project Consortium, Moore, J.E., Purcaro, M.J., Pratt, H.E., Epstein, C.B., Shoresh, N., Adrian, J., Kawli, T., Davis, C.A., and Dobin, A. (2022). Author Correction: Expanded encyclopaedias of DNA elements in the human and mouse genomes. Nature *605*, E3.
36. GTEx Consortium (2013). The Genotype-Tissue Expression (GTEx) project. Nat. Genet. *45*, 580–585. https://doi.org/10.1038/ng.2653.
37. Patowary, A., Zhang, P., Jops, C., Vuong, C.K., Ge, X., Hou, K., Kim, M., Gong, N., Margolis, M., Vo, D., et al. (2024). Developmental isoform diversity in the human neocortex informs neuropsychiatric risk mechanisms. Science *384*, eadh7688. https://doi.org/10.1126/science.adh7688.
38. Reese, F., Williams, B., Balderrama-Gutierrez, G., Wyman, D., Çelik, M.H., Rebboah, E., Rezaie, N., Trout, D., Razavi-Mohseni, M., Jiang, Y., et al. (2023). The ENCODE4 long-read RNA-seq collection reveals distinct classes of transcript structure diversity. Preprint at bioRxiv. https://doi.org/10.1101/2023.05.15.540865.
39. Zhou, Y., Pan, Q., Pires, D.E.V., Rodrigues, C.H.M., and Ascher, D.B. (2023). DDMut: predicting effects of mutations on protein stability using deep learning. Nucleic Acids Res. *51*, W122–W128. https://doi.org/10.1093/nar/gkad472.
40. Popov-Celeketić, D., Mapa, K., Neupert, W., and Mokranjac, D. (2008). Active remodelling of the TIM23 complex during translocation of preproteins into mitochondria. EMBO J. *27*, 1469–1480. https://doi.org/10.1038/emboj.2008.79.
41. Warren, E.B., Gordon-Lipkin, E.M., Cheung, F., Chen, J., Mukherjee, A., Apps, R., Tsang, J.S., Jetmore, J., Schlein, M.L., Kruk, S., et al. (2023). Inflammatory and interferon gene expression signatures in patients with mitochondrial disease. J. Transl. Med. *21*, 331. https://doi.org/10.1186/s12967-023-04180-w.
42. Kapnick, S.M., Pacheco, S.E., and McGuire, P.J. (2018). The emerging role of immune dysfunction in mitochondrial diseases as a paradigm for understanding immunometabolism. Metabolism *81*, 97–112. https://doi.org/10.1016/j.metabol.2017.11.010.
43. Landrum, M.J., Lee, J.M., Benson, M., Brown, G.R., Chao, C., Chitipiralla, S., Gu, B., Hart, J., Hoffman, D., Jang, W., et al. (2018). ClinVar: improving access to variant interpretations and supporting evidence. Nucleic Acids Res. *46*, D1062–D1067. https://doi.org/10.1093/nar/gkx1153.
44. ClinGen Consortium Electronic address splon@bcm edu; and ClinGen Consortium (2025). The Clinical Genome Resource (ClinGen): Advancing genomic knowledge through global curation. Genet. Med. *27*, 101228. https://doi.org/10.1016/j.gim.2024.101228.
45. Firth, H.V., Richards, S.M., Bevan, A.P., Clayton, S., Corpas, M., Rajan, D., Van Vooren, S., Moreau, Y., Pettett, R.M., and Carter, N.P. (2009). DECIPHER: Database of Chromosomal Imbalance and Phenotype in Humans Using Ensembl Resources. Am. J. Hum. Genet. *84*, 524–533. https://doi.org/10.1016/j.ajhg.2009.03.010.
46. Sánchez-Gaya, V., Mariner-Faulí, M., and Rada-Iglesias, A. (2020). Rare or Overlooked? Structural Disruption of Regulatory Domains in Human Neurocristopathies. Front. Genet. *11*, 688. https://doi.org/10.3389/fgene.2020.00688.
47. Pande, S., Dawood, M., and Grochowski, C.M. (2025). Structural Variants: Mechanisms, Mapping, and Interpretation in Human Genetics. Genes *16*, 905. https://doi.org/10.3390/genes16080905.
48. Liu, Z., Roberts, R., Mercer, T.R., Xu, J., Sedlazeck, F.J., and Tong, W. (2022). Towards accurate and reliable resolution of structural variants for clinical diagnosis. Genome Biol. *23*, 68. https://doi.org/10.1186/s13059-022-02636-8.
49. Scott, A.J., Chiang, C., and Hall, I.M. (2021). Structural variants are a major source of gene expression differences in humans and often affect multiple nearby genes. Genome Res. *31*, 2249–2257. https://doi.org/10.1101/gr.275488.121.
50. Sagai, T., Hosoya, M., Mizushina, Y., Tamura, M., and Shiroishi, T. (2005). Elimination of a long-range cis-regulatory module causes complete loss of limb-specific Shh expression and truncation of the mouse limb. Development *132*, 797–803. https://doi.org/10.1242/dev.01613.
51. Kleinjan, D.A., and van Heyningen, V. (2005). Long-range control of gene expression: emerging mechanisms and disruption in disease. Am. J. Hum. Genet. *76*, 8–32. https://doi.org/10.1086/426833.
52. Monahan, K., Horta, A., and Lomvardas, S. (2019). LHX2- and LDB1-mediated trans interactions regulate olfactory receptor choice. Nature *565*, 448–453. https://doi.org/10.1038/s41586-018-0845-0.
53. Bashkirova, E., and Lomvardas, S. (2019). Olfactory receptor genes make the case for inter-chromosomal interactions. Curr. Opin. Genet. Dev. *55*, 106–113. https://doi.org/10.1016/j.gde.2019.07.004.
54. Harner, M.K., Bishop, D.V., Pollak, R.M., Purcell, R.H., and Mulle, J.G. (2025). Copy Number Variants: Deletion and Duplication Syndromes. Annu. Rev. Genomics Hum. Genet. *26*, 261–277. https://doi.org/10.1146/annurev-genom-121222-120601.
55. Smajlagić, D., Lavrichenko, K., Berland, S., Helgeland, Ø., Knudsen, G.P., Vaudel, M., Haavik, J., Knappskog, P.M., Njølstad, P.R., Houge, G., and Johansson, S. (2021). Population prevalence and inheritance pattern of recurrent CNVs associated with neurodevelopmental disorders in 12,252 newborns and their parents. Eur. J. Hum. Genet. *29*, 205–215. https://doi.org/10.1038/s41431-020-00707-7.
56. Goh, S., Dudding-Byth, T., Pinese, M., and Kirk, E.P. (2026). Updated penetrance estimates for recurrent copy number variants - an improved definition and formula. Eur. J. Hum. Genet. *34*, 119–127. https://doi.org/10.1038/s41431-025-01948-0.
57. Wang, F., Peng, H., Lou, G., Ren, Y., and Liao, S. (2024). Prenatal ultrasound phenotype of fetuses with recurrent 1q21.1 deletion and duplication syndrome. Front. Pediatr. *12*, 1504122. https://doi.org/10.3389/fped.2024.1504122.
58. Dastan, J., Chijiwa, C., Tang, F., Martell, S., Qiao, Y., Rajcan-Separovic, E., and Lewis, M.E.S. (2016). Exome sequencing identifies pathogenic variants of VPS13B in a patient with familial 16p11.2 duplication. BMC Med. Genet. *17*, 78. https://doi.org/10.1186/s12881-016-0340-0.
59. Pehlivan, D., Bengtsson, J.D., Bajikar, S.S., Grochowski, C.M., Lun, M.Y., Gandhi, M., Jolly, A., Trostle, A.J., Harris, H.K., Suter, B., et al. (2024). Structural variant allelic heterogeneity in MECP2 duplication syndrome provides insight into clinical severity and variability of disease expression. Genome Med. *16*, 146. https://doi.org/10.1186/s13073-024-01411-7.

HGGA, Volume 7

Supplemental information

Diagnostic utility of clinical genome reanalysis

in rare pediatric disorders using long-read sequencing

Elizabeth A. Werren, Purva Vats, Gabriel E. Rech, Michael Peracchio, Cameron King, Elizabeth J. Charnysh, Ryan D. Gorham, Peter A. Audano, Peter N. Robinson, Melissa A. Kelly, Adam P. Matson, Mark D. Adams, and Louisa Kalsner

TABLE OF CONTENTS

SUPPLEMENTAL NOTE: CLINICAL SUMMARIES 2
FIGURES 9
MATERIALS AND METHODS 18
REFERENCES 25

Supplemental Note: Clinical Summaries

8442P: Three-year-old male with hypotonia, global developmental delay, bilateral retinal detachment, and failure to thrive. He had feeding intolerance as a newborn and had pyloric stenosis repair and then gastrostomy tube placement and required jejunal feeding. He had a large patent ductus arteriosus which was closed surgically. He had abnormal eye movements and was diagnosed with bilateral retinal detachment. He can sit and scoot but does not walk. He uses a few words and signs. On exam he has microcephaly (Z= -2.8), with normal weight and height. He has simply shaped, posteriorly rotated ears and deep-set eyes. He has roving eye movements with intermittent nystagmus and irregular, non-reactive pupils. He has hypotonia and reduced reflexes. MRI brain showed prominent ventricles and sulci and abnormal signal within the globes of both eyes.

2598P: Four-year-old male with hypotonia, speech delay, hearing loss, and unsteady gait. Hypotonia was noted by one year of age and he walked late, after age two. He has mild to moderate high frequency sensorineural hearing loss bilaterally and wears hearing aids. On exam there are no dysmorphic features. He has scanning quality to his speech with articulation difficulty. He has hypotonia with mild ataxia and dysmetria. He had normal brain MRI.

1048P-1: Nine-year-old male with hypotonia, global developmental delay with intellectual disability, and history of epilepsy with infantile spasms. He had feeding difficulty at birth. He presented with infantile spasms at two months of age, successfully treated with ACTH. He is non-ambulatory and non-verbal. He has history of obstructive sleep apnea. Exam is notable for short stature (Z= -2), brachycephaly with relatively larger head circumference (Z=0) and a broad forehead. He has bilateral esotropia, diffuse hypotonia, and reduced reflexes. MRI of the brain was normal.

1048P-2: Four-year-old male with global developmental delay, hypotonia, and failure to thrive. He is non-ambulatory and non-verbal. He had gastrostomy tube placed due to failure to thrive but has continued poor weight gain. He has thoracic scoliosis. He had surgery to address esotropia of the right eye with amblyopia. Exam is notable for small size involving weight (Z= -3) and height (Z = -2.2) with relatively larger head size (Z=0). MRI spine identified a cauda equina arachnoid cyst. Brain MRI is normal.

7170P: Sixteen-year-old female with multiple medical issues including severe GI dysmotility, recurrent sinopulmonary infections, progressive dystonia of the right foot, postural orthostatic tachycardia syndrome (POTS), and joint hypermobility. She receives IVIG infusions for possible common variable immune deficiency (CVID). She had rectal prolapse and severe constipation leading to appendicocecostomy. Dystonia of the right foot began at 14 years of age and is treated with botulinum toxin injections. Physical exam is notable for dystonia of the right foot and brisk reflexes in both lower extremities. She had normal MRI of the brain and spine and normal nerve conduction study.

3982P: Eleven-year-old male with hypotonia, autism spectrum disorder, intellectual disability and epilepsy. He remains non-verbal and has prominent stereotypies. He drinks formula only and will not eat solid foods. Seizures began at age six and are controlled with two anti-epileptic medications. He has persistent hypotonia and tires quickly when ambulating. Exam is notable for triangular facial shape with widely spaced teeth, and long fingers and toes. EEG showed right sided focal spikes. MRI brain was normal.

8779P: Eight-year-old female with global developmental impairment and refractory epilepsy. She had feeding difficult with poor weight gain in infancy. She remains non-ambulatory and non-verbal.

Seizures began at age three years and have been refractory to treatment. She has daily seizures despite treatment with three anti-epileptic medications. She has amblyopia. On exam she has relative macrocephaly, large simply shaped ears, high arched palate, narrow nose and furrowed tongue. She has facial weakness and diffuse hypotonia. She had a normal brain MRI.

9548P: Nine-year-old male with global developmental delay with intellectual disability, movement disorder and epilepsy. He had failure to thrive in infancy leading to gastrostomy tube placement which continues to provide most of his nutrition. He developed chorea in early childhood and later dystonia managed with trihexyphenidyl and amantadine. He had two episodes of status epilepticus at age seven and is seizure free on levetiracetam. He is non-ambulatory and can speak in single words. He has normal growth parameters. On exam, he has no dysmorphic features. He has hypotonia, orofacial and limb dystonia, and choreiform movements. He has diffuse hypomyelination on brain MRI.

3162P: Four-year-old female with severe global developmental delay, feeding difficulty and mild hepatomegaly. She can sit briefly without support and can crawl for short distances. She is non-verbal. Diet is limited to formula and pureed foods. She has mildly enlarged liver on ultrasound with normal liver enzymes. Physical exam is notable for low weight and height ($Z < -2$). She has partially erupted teeth and prominent gums, full cheeks, heavy eyebrows, and up-turned nose. She has mildly distended abdomen with translucent skin and prominent veins over her chest. She has hypotonia and reduced muscle bulk in her legs with brisk reflexes. Brain MRI was notable for scattered non-specific signal change in the supratentorial white matter.

3534P: Seven-year-old male with refractory epilepsy, spastic quadriparesis and global developmental impairment. He was non-ambulatory and non-verbal. He had daily seizures despite treatment with three anti-epileptic medications. He was fed by gastrostomy tube due to

oropharyngeal dysphagia. Head imaging showed progressive cerebral volume loss. Family history was notable for parents being first cousins. He had a progressive decline in neurological function including autonomic dysfunction. He passed away in the setting of viral pneumonia with respiratory failure at the age of nine.

4161P: Six-year-old male with global developmental delay and autism spectrum disorder. He had gross motor delay, walking independently at 27 months of age. He used a few words but had mild regression after age three and is now nonverbal. He had a gastrostomy tube placed to augment nutrition as he takes only formula and purees by mouth. He has depressed nasal bridge, long eyelashes and prominent forehead as well as mild diffuse hypotonia. He had normal brain MRI.

6600P: Sixteen-year-old female with refractory epilepsy beginning at 6 months of age with infantile spasms and later evolution to Lennox-Gastaut syndrome. Her epilepsy is refractory to treatment with numerous medications, vagal nerve stimulator, and ketogenic diet and she continues to have daily seizures. She has severe global developmental impairment with loss of skills such as ability to crawl and ambulate with a walker. She is non-verbal. She has a gastrostomy tube for nutrition due to poor weight gain. She has intermittent hand wringing and unusual respiratory pattern with periodic hyperventilation followed by breath holding. Physical exam is notable for low weight and height ($Z = < -3$ SD). She has small ears, mild prognathism, and smooth philtrum. She has a broad chest and abdomen, narrow feet, and long toes. She has increased tone in her extremities with brisk reflexes. MRI is notable for cavum septum vergae and mild cerebellar vermian hypoplasia or volume loss.

3888P: Nine-year-old female with global developmental impairment and refractory epilepsy and clinical diagnosis of Aicardi syndrome. Seizures began on the third day of life. She had gastrostomy tube placed due to oropharyngeal dysphagia. She had refractory epileptic spasms,

not responding to treatment with steroids and multiple medications and has persistent myoclonic and tonic-clonic seizures. She can sit with support but cannot crawl or walk and is non-verbal. On exam, she has short stature (Z= -2) and microcephaly (Z= -2.4). She has left-sided coloboma and microphthalmia. She has up-turned nares and ears with over-folded superior helix. She has axial weakness with spastic diplegia of her lower extremities with brisk reflexes and contractures at the knees. Brain MRI shows bilateral peri-sylvian polymicrogyria and subependymal nodular heterotopia along the margins of the lateral ventricles. The posterior aspect of the corpus callosum is hypoplastic and dysmorphic and massa intermedia is enlarged.

3235P: Two-and-a-half-year-old male who had status epilepticus in the setting of a febrile illness at 22 months of age and was found to have leukodystrophy on brain imaging. He has mild developmental delay, walking at 17 months of age, with persistent unsteady gait. He has speech delay, using only a few words. He is non-dysmorphic on exam, though with borderline microcephaly (Z= -1.7) and mildly ataxic gait. Brain MRI revealed extensive confluent signal abnormality involving the cerebral and cerebellar white matter suggestive of a leukoencephalopathy. Immune and infectious work-up was negative.

1195P: Twenty-three-year-old male with autism spectrum disorder, intellectual disability, short stature, sensorineural hearing loss, and epilepsy. He has a history of hypospadias with penoscrotal transposition repaired in childhood. He has central hypothyroidism. Seizures began at 11 months of age, and he remains on medication for focal epilepsy. He is non-verbal and has progressive sensorineural hearing loss, now severe to profound bilaterally. He has aggressive behavior treated with aripiprazole. Physical exam is notable for short stature (Z= -2.5) and bifid uvula. Brain MRI is normal.

9909P: Two-year-old male with global developmental delay and dysmorphic features. He has dysphagia and does not eat solid foods. He can sit but cannot walk. He has pointy shaped ears with bow shaped upper lip, flattened nasal bridge, and small nose. He has hypotonia and stereotypic hand movements. Brain MRI was normal.

2990P: Eighteen-year-old female with autism spectrum disorder, intellectual disability and epilepsy. She was diagnosed with autism at age two. She uses a few single words. She has convulsive seizures treated with topiramate. EEG demonstrated focal and generalized spike wave discharges. MRI brain showed mild cerebellar tonsillar ectopia.

3787P: Newborn male with severe metabolic decompensation. Baby was born at term with birth weight of 7 lb 9 oz. Apnea was noted shortly after delivery, and he was intubated and required chest compressions. He had severe metabolic acidosis with pH of 6.63. Ammonia was elevated to 507, and lactic acid rose to > 20 raising concern for an inborn error of metabolism. He had bleeding with disseminated intravascular coagulation (DIC). He developed abnormal posturing and therapeutic cooling was initiated. He had profound hypotonia and developed fixed, dilated pupils. He expired at 24 hours of life after withdrawal of care.

7191P: One-year-old female born prematurely at 26.5 weeks with agenesis of corpus callosum, dysmorphic facial features and persistent oropharyngeal dysphagia requiring gastrostomy tube. She has had slow weight gain, shorter stature and microcephaly (Z= -2.2). She remains mostly tube fed. She has short palpebral fissures, and small nose and mouth with flattened appearance to facial features. She has trichiasis of the lower eyelids and nasolacrimal duct obstruction. MRI revealed agenesis of the corpus callosum and small optic nerves and chiasm.

9646P: Eight-year-old female with muscle weakness and autism spectrum disorder. She walked at 16 months of age but has persistent weakness with positive Gower maneuver. She has mild scoliosis. She was diagnosed with megalocornea without glaucoma. She has macrocephaly (Z= 2.2) and partial 2/3 syndactyly of the toes. She has reduced muscle bulk in her legs with weakness and trace to absent lower extremity reflexes. She had normal CPK and normal EMG/nerve conduction study. She had normal MRI of brain and lumbar spine.

8784P: Twenty-one-year-old male with intellectual disability, autism, and epilepsy. He had developmental delay noted in the first months of life. He was able to sit at four years and began taking steps at 10 years. He is non-verbal. He has contractures at the knees but can take a few steps independently. He has thoracic scoliosis. Tonic seizures began at 19 years of age and are controlled with lamotrigine. He did not lose his deciduous teeth. On exam, he has microcephaly (Z = -4.5), short stature (Z= -2.7) and low weight (Z= -2). He has prognathism and prominent columella, prominent interphalangeal joints and small feet with hammer toes. He has pectus excavatum and reduced muscle bulk, and walks with crouched gait. Brain MRI was normal.

Figures

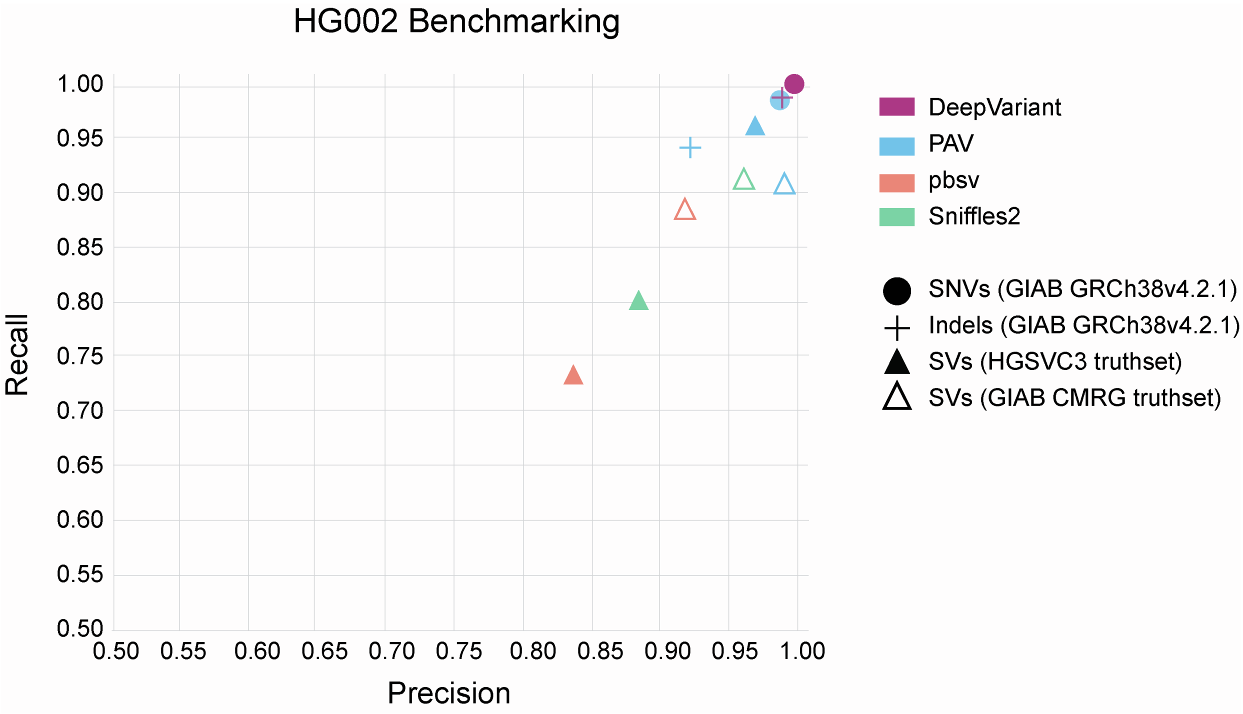

Figure S1. Benchmarking variant calling in LR-GS pipeline. Concordance plot of precision vs. recall values across variant callers (DeepVariant (dark blue), PAV (light blue), pbsv (purple), Sniffles2 (green)) and variant types (SNVs (·), indels (+), SVs (▲)) against the benchmarks: HG002 GIAB GRCh38 v4.2.1 (SNVs/indels), HGSVC3 (SVs, ▲), and GIAB challenging medically relevant genes (CMRG, SVs, △). For small variants, the highest precision (p) and recall (r) was obtained for DeepVariant SNV calls (p=99.9%, r=99.9%), followed by DeepVariant indels (p= 98.9%, r=98.6%), PAV SNVs (p= 98.7%, r=98.3%), and lastly PAV indels (p=92.3%, r=94.0%). For SVs, the highest concordance was observed for PAV (p=96.9%, r=96.1%), followed by Sniffles2 (p=88.4%, r=80.1%), and lastly pbsv (p=83.7%, r=73.3%). We further compared with the GIAB challenging medically relevant gene calls and find the highest precision for PAV (p=99.0%, r=90.7%), highest recall for Sniffles2 (p=96.1%, r=91.2%), and the lowest prevision and recall for pbsv (p=91.8%, r=88.4%).

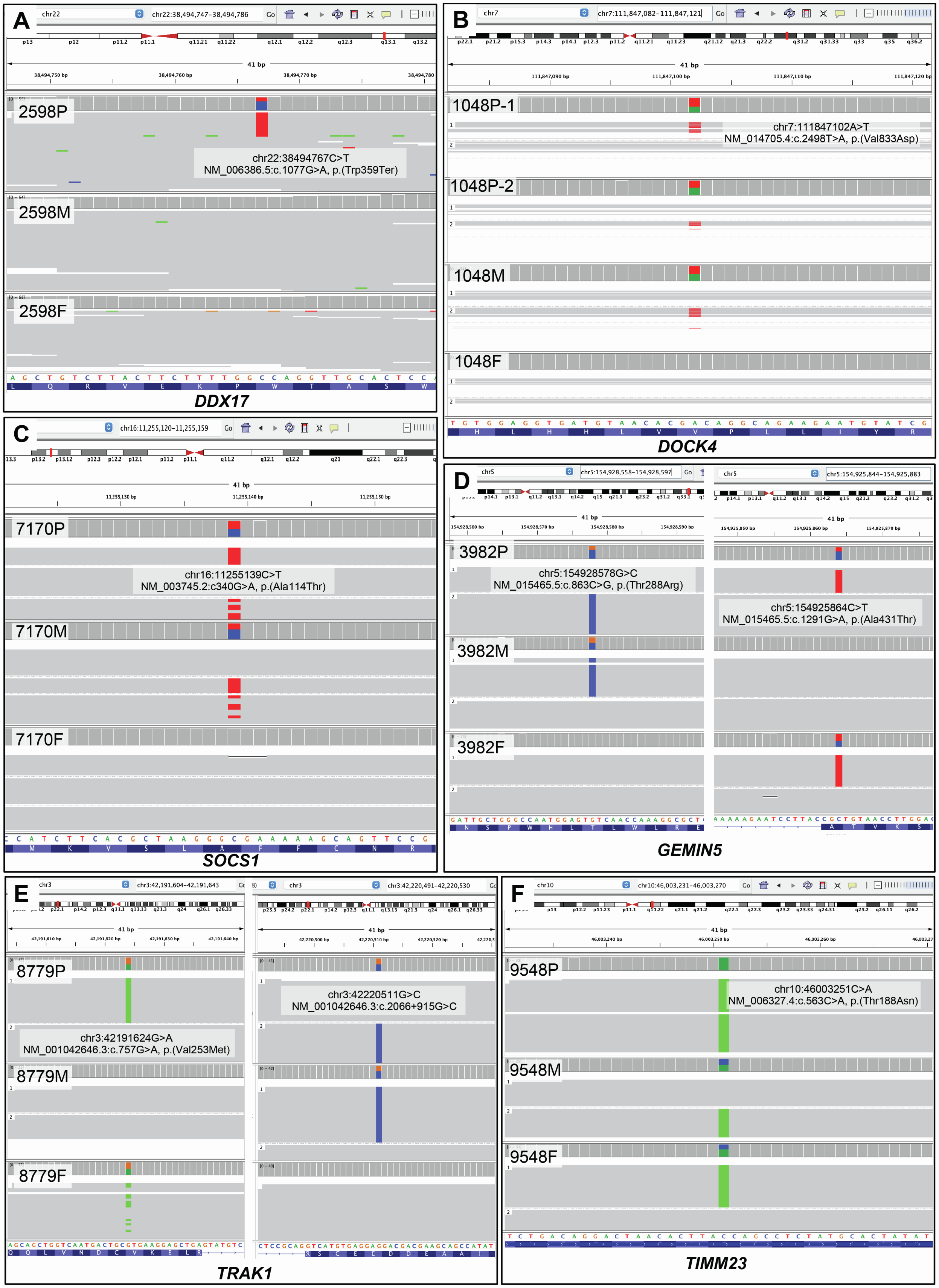
A
2598P
chr22:38494767C>T
NM_006386.5:c.1077G>A, p.(Trp359Ter)
2598M
2598F
DDX17
B
1048P-1
chr7:111847102A>T
NM_014705.4:c.2498T>A, p.(Val833Asp)
1048P-2
1048M
1048F
DOCK4
C
7170P
chr16:11255139C>T
NM_003745.2:c340G>A, p.(Ala114Thr)
7170M
7170F
SOCS1
D
3982P
chr5:154928578G>C
NM_015465.5:c.863C>G, p.(Thr288Arg)
chr5:154925864C>T
NM_015465.5:c.1291G>A, p.(Ala431Thr)
3982M
3982F
GEMIN5
E
8779P
chr3:42191624G>A
NM_001042646.3:c.757G>A, p.(Val253Met)
chr3:42220511G>C
NM_001042646.3:c.2066+915G>C
8779M
8779F
TRAK1
F
9548P
chr10:46003251C>A
NM_006327.4:c.563C>A, p.(Thr188Asn)
9548M
9548F
TIMM23

Figure S2. IGV assessment of clinically confirmed variants in Illumina SR bams aligned to hg38 no-alt reference for family 2598 (A) and in PacBio HiFi whatshap-phased bams aligned to hg38 no-alt reference, grouped by phase for families 1048 (B), 7170 (C), 3982 (D), 8779 (E), 9548 (F).

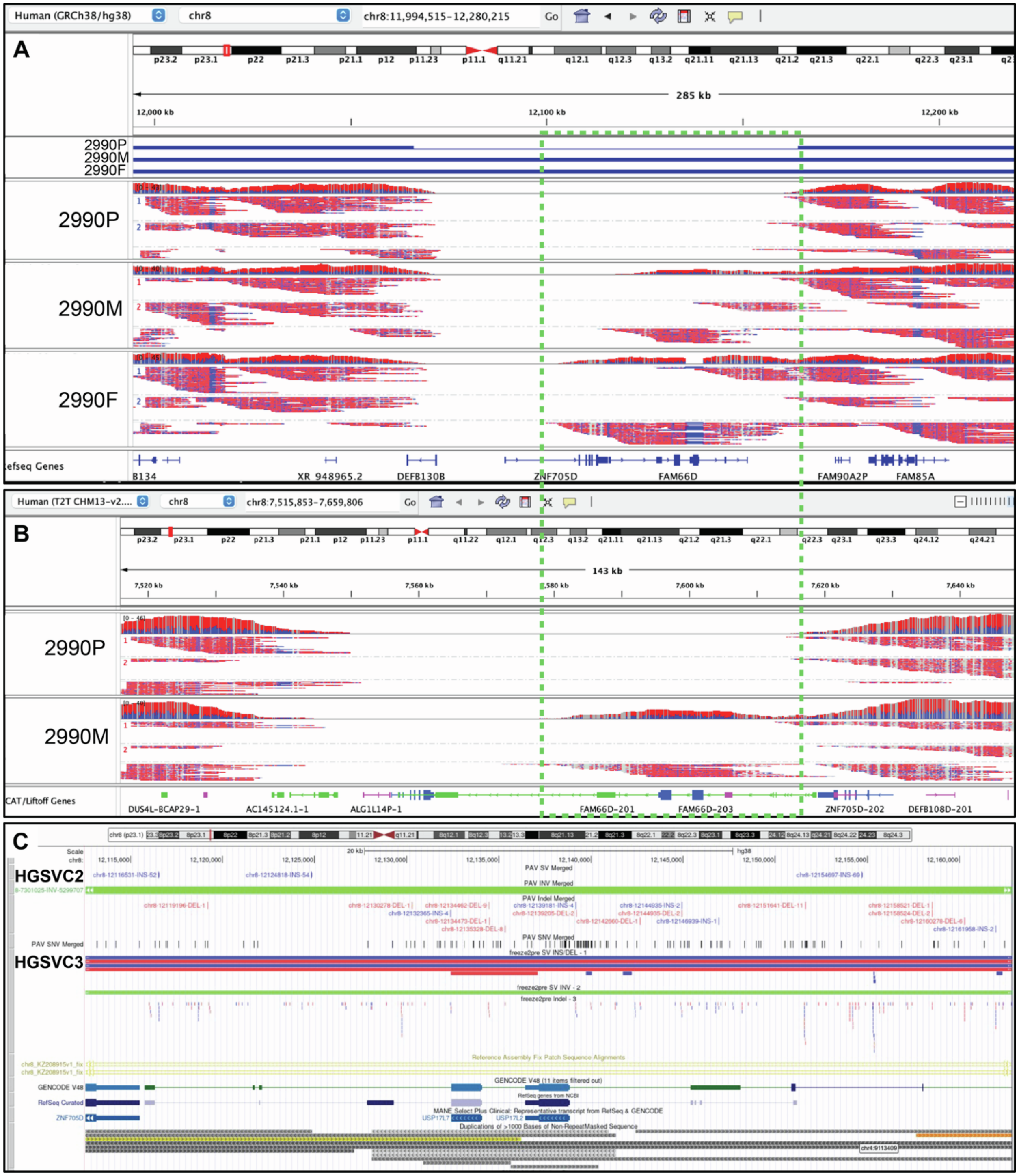

Figure S3. 8p23.1 deletion in family 2990 in (A) IGV of whatshap-phased bams aligned to reference hg38, with HiFiCNV copy number bedgraphs (top, blue), and (B) IGV of 2990P and 2990M whatshap-phased bams aligned to reference hs1 (T2T CHM13-v2.0). Tracks colored by 5mC tag and grouped by phase. Green box shows homozygous deletion region in 2990P. C) UCSC Genome Browser with HGSVC2 and HGSVC3 track hubs displayed, showing SVs in general population within region.

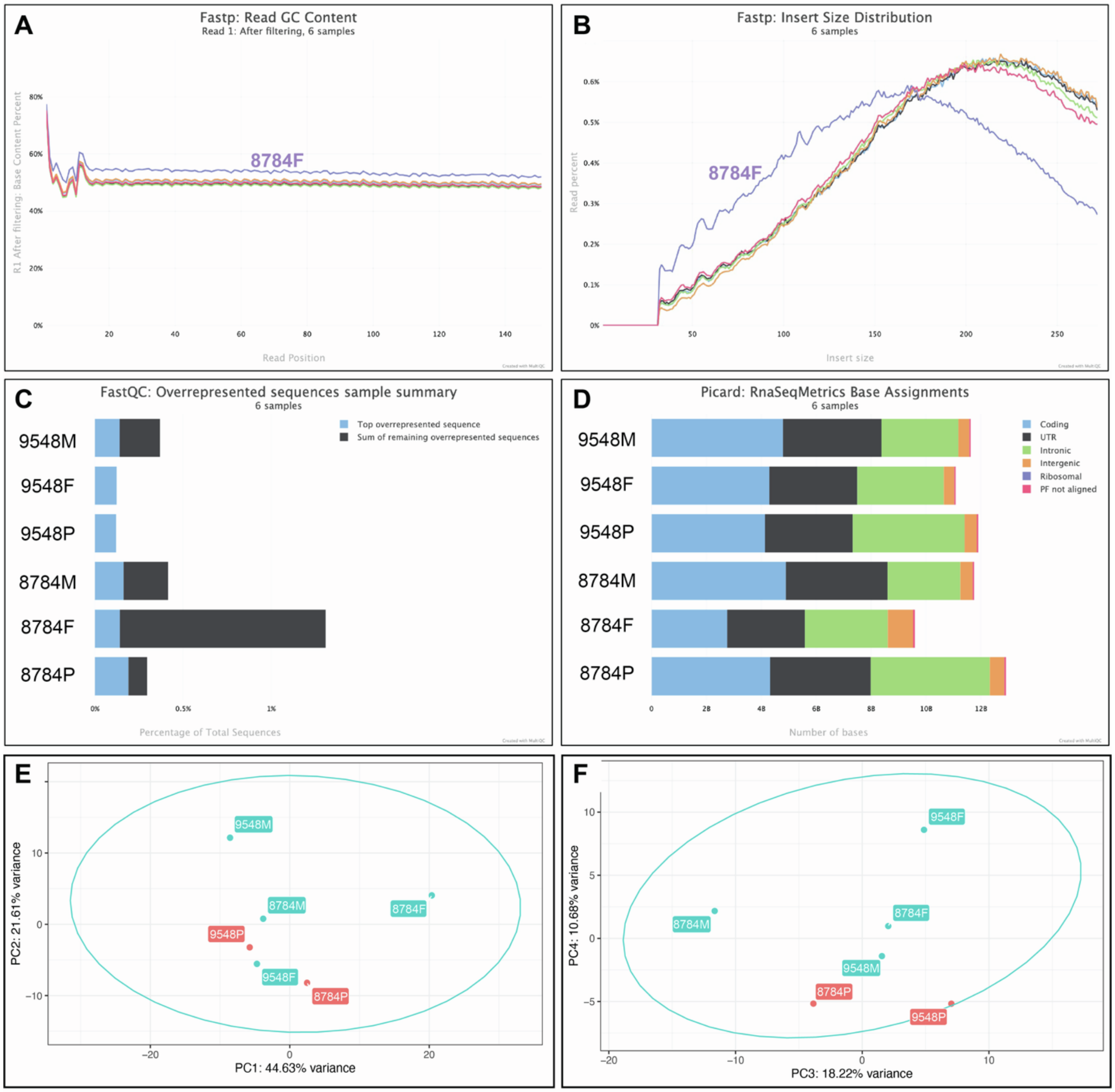

Figure S4. Quality metrics of PBMC RNAseq data for family trios 8784 and 9548. A) Fastp read GC content. B) Fastp insert size distribution. C) FastQC summary of overrepresented sequences. D) Picard base assignments. PCA plot of PC1 vs PC2 (E), and PC3 vs PC4 (D). Red represents affected children; blue represents unaffected parents; ellipse represents the 95% confidence interval.

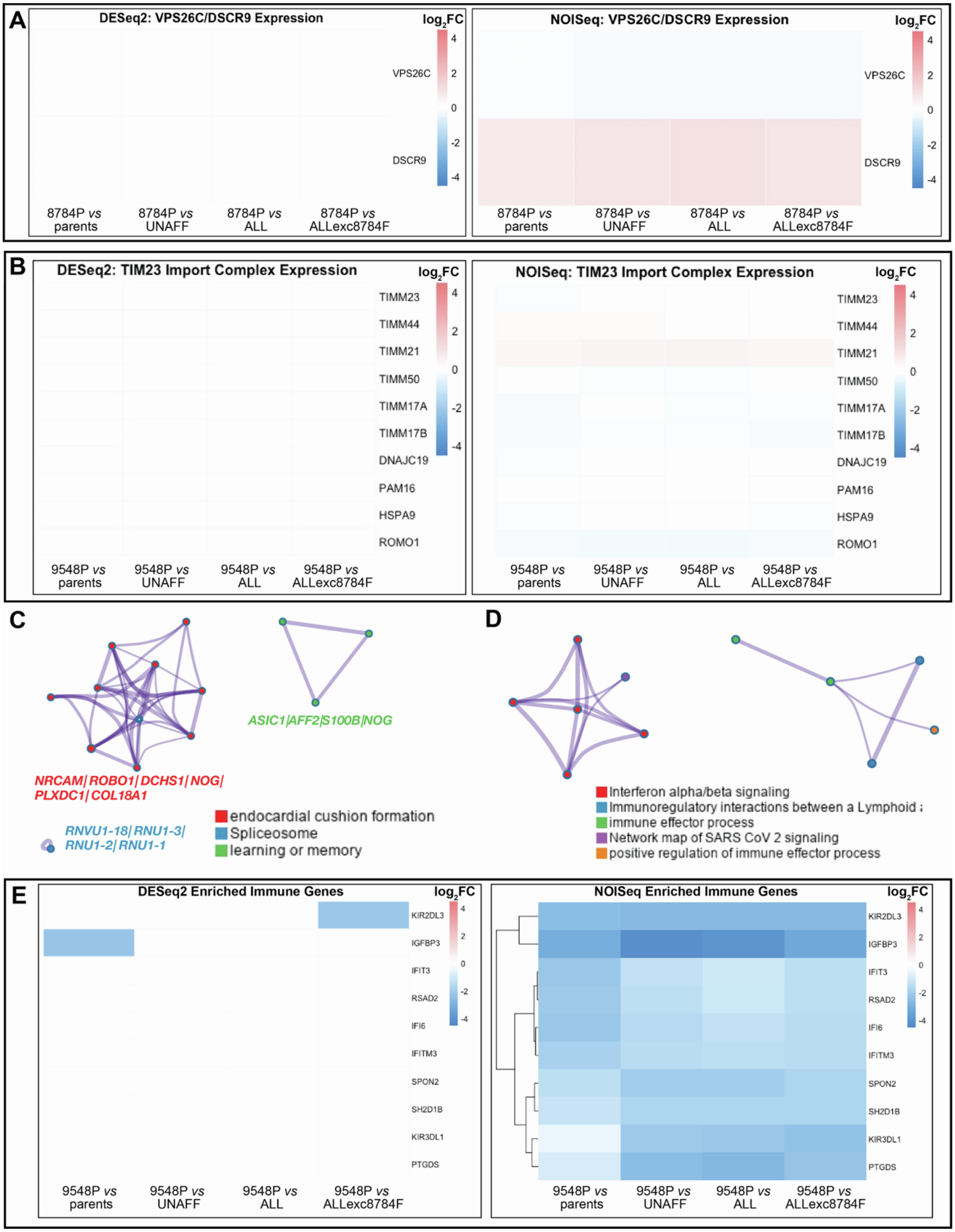

Figure S5. PBMC RNAseq findings in family trios 8784 and 9548. A) Heatmaps of DESeq2 and NOISeq Log_2FC data across expression comparisons for (A) *VPS26C* (and nearby lncRNA

DSCR9) in 8784P and (B) TIM23 complex members in 9548P. Comparisons: UNAFF, all unaffected adults; ALL, all other samples; ALLexc8784F, all other samples excluding 8784F. (C) MetaScape network graphs of enriched pathway and processes in genes showing elevated (C) and reduced (D) expression in 9548P PBMCs compared to unaffected PBMCs. (E) Heatmaps of DESeq2 and NOISeq Log_2FC data across expression comparisons of negatively enriched immune signaling genes identified in the MetaScape enrichment analysis.

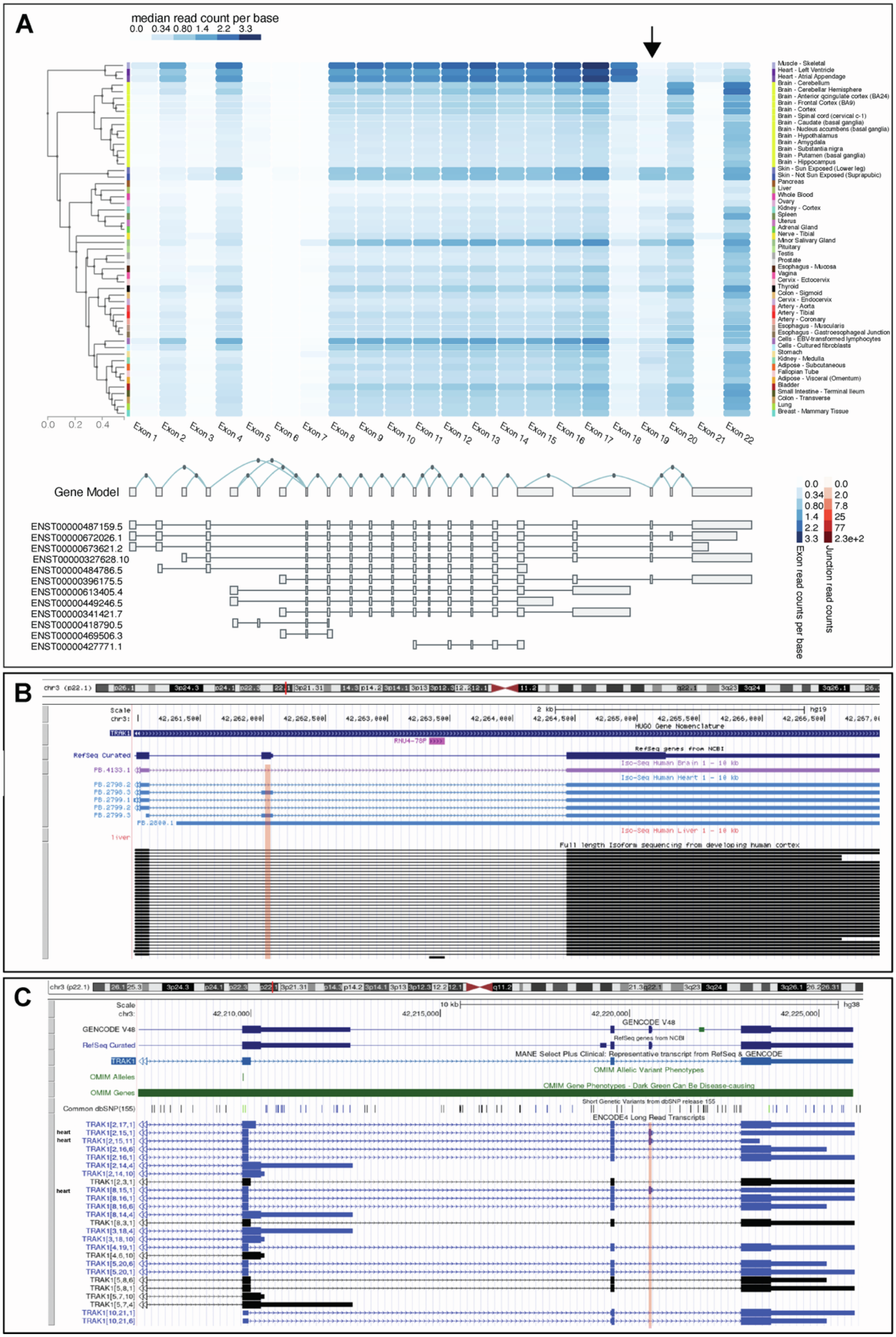
A
median read count per base
0.0 0.34 0.80 1.4 2.2 3.3
Muscle - Skeletal
Heart - Left Ventricle
Heart - Atrial Appendage
Brain - Cerebellum
Brain - Cerebellar Hemisphere
Brain - Anterior qcingulate cortex (BA24)
Brain - Frontal Cortex (BA9)
Brain - Cortex
Brain - Spinal cord (cervical c-1)
Brain - Caudate (basal ganglia)
Brain - Nucleus accumbens (basal ganglia)
Brain - Hypothalamus
Brain - Amygdala
Brain - Substantia nigra
Brain - Putamen (basal ganglia)
Brain - Hippocampus
Skin - Sun Exposed (Lower leg)
Skin - Not Sun Exposed (Suprapubic)
Pancreas
Liver
Whole Blood
Ovary
Kidney - Cortex
Spleen
Uterus
Adrenal Gland
Nerve - Tibial
Minor Salivary Gland
Pituitary
Testis
Prostate
Esophagus - Mucosa
Vagina
Cervix - Ectocervix
Thyroid
Colon - Sigmoid
Cervix - Endocervix
Artery - Aorta
Artery - Tibial
Artery - Coronary
Esophagus - Muscularis
Esophagus - Gastroesophageal Junction
Cells - EBV-transformed lymphocytes
Cells - Cultured fibroblasts
Stomach
Kidney - Medulla
Adipose - Subcutaneous
Fallopian Tube
Adipose - Visceral (Omentum)
Bladder
Small Intestine - Terminal Ileum
Colon - Transverse
Lung
Breast - Mammary Tissue
0.0 0.2 0.4
Exon 1 Exon 2 Exon 3 Exon 4 Exon 5 Exon 6 Exon 7 Exon 8 Exon 9 Exon 10 Exon 11 Exon 12 Exon 13 Exon 14 Exon 15 Exon 16 Exon 17 Exon 18 Exon 19 Exon 20 Exon 21 Exon 22
Gene Model
ENST00000487159.5
ENST00000672026.1
ENST00000673621.2
ENST00000327628.10
ENST00000484786.5
ENST00000396175.5
ENST00000613405.4
ENST00000449246.5
ENST00000341421.7
ENST00000418790.5
ENST00000469506.3
ENST00000427771.1
0.0 0.34 0.80 1.4 2.2 3.3
Exon read counts per base
0.0 2.0 7.8 25 77 2.3e+2
Junction read counts
B
chr3 (p22.1)
Scale
chr3:
2 kb
hg19
42,261,500 42,262,000 42,262,500 42,263,000 42,263,500 42,264,000 42,264,500 42,265,000 42,265,500 42,266,000 42,266,500
HUGO Gene Nomenclature
TRAK1
RNU4-78P
RefSeq genes from NCBI
RefSeq Curated
Iso-Seq Human Brain 1 - 10 kb
PB.4133.1
Iso-Seq Human Heart 1 - 10 kb
PB.2798.2
PB.2798.3
PB.2799.1
PB.2799.2
PB.2799.3
PB.2800.1
Iso-Seq Human Liver 1 - 10 kb
liver
Full length Isoform sequencing from developing human cortex
C
chr3 (p22.1)
Scale
chr3:
10 kb
hg38
42,210,000 42,215,000 42,220,000 42,225,000
GENCODE V48
RefSeq Curated
RefSeq genes from NCBI
TRAK1
MANE Select Plus Clinical: Representative transcript from RefSeq & GENCODE
OMIM Alleles
OMIM Allelic Variant Phenotypes
OMIM Genes
OMIM Gene Phenotypes - Dark Green Can Be Disease-causing
Common dbSNP(155)
Short Genetic Variants from dbSNP release 155
ENCODE4 Long Read Transcripts
TRAK1[2,17,1]
heart TRAK1[2,15,1]
heart TRAK1[2,15,11]
TRAK1[2,16,6]
TRAK1[2,16,1]
TRAK1[2,14,4]
TRAK1[2,14,10]
TRAK1[2,3,1]
heart TRAK1[8,15,1]
TRAK1[8,16,1]
TRAK1[8,16,6]
TRAK1[8,14,4]
TRAK1[8,3,1]
TRAK1[3,18,4]
TRAK1[3,18,10]
TRAK1[4,19,1]
TRAK1[4,6,10]
TRAK1[5,20,6]
TRAK1[5,20,1]
TRAK1[5,8,6]
TRAK1[5,8,1]
TRAK1[5,7,10]
TRAK1[5,7,4]
TRAK1[10,21,1]
TRAK1[10,21,6]

Figure S6. Expression data of *TRAK1* alternative isoform predicted to harbor NM_001349247.2:c.2079G>C, p.(Glu693Asp) in individual 8779C. A) Heat map of exon expression as median read counts per base for TRAK1 isoforms across different adult tissue types in the Adult Genotype Tissue Expression (GTEx) database, with isoform maps illustrated below.[1] Black arrow pointing to exon 19 which harbors NM_001349247.2:c.2079. UCSC Genome Browser screenshot surrounding NM_001349247.2:c.2079 region (red box) in PacBio Iso-Seq data from human brain, heart, and liver as well as developing cortex[2] in hg19 (B) and (C) the ENCODE long-read transcript dataset in hg38.[3]

Materials and Methods

Participant Recruitment and Informed Consent Procedures

All study participants were identified via their medical provider, or if they had previously participated in other suspected genetic disorder studies, at Connecticut Children's (CC). Participant eligibility was determined by CC and The Jackson Laboratory for Genomic Medicine (JGM) investigators based on the following criteria: age (21 or under), medical and family history related to a suspected inherited disorder, and inconclusive prior genetic testing findings. Participants under 18 years of age were approached only if parent(s) were present. If eligible and interested, parents or adult participants were then referred to a genetic counselor within the study team for pre-enrollment counseling.

Following pre-enrollment counseling, interested parents or adult participants were provided a copy of the IRB-approved Informed Consent Form/HIPAA Authorization form (ICF) followed by a consent discussion. A designated member of the study team reviewed the ICF section by section in a private area at CC or via phone based on participant preference, encouraging questions and providing thorough responses. Parents who elected to participate with their child also completed a separate form.

Once complete, participants were asked if they wanted to join the study and were given the option to receive clinical findings (if any) after confirmation through a clinically ordered confirmatory test. Results not clinically confirmed were not returned. Results of confirmatory testing were entered into the affected participant's medical record and shared with their provided clinical care provider. A signed copy of the ICF by both participants and study team member was provided to all participants. Informed consent included the authorization for the use and disclosure of their Protected Health Information collected for use in this study by both CC and JGM study investigators. All enrolled participants were assigned a unique participant identifier (PID). The

original signed ICF was stored separately from the research record and with other study documents that contain personal identifiers.

Genome sequencing and analysis

Venous whole blood samples (at least 2 mL) were obtained from all consented subjects at CC or at Quest Diagnostics—CLIA/CAP-certified clinical laboratory and a contracted service provider for CC—using standard collection procedures and EDTA tubes. Labeled tubes were then securely transported to JGM within 24-48 hours of collection. Research trio/quad LR-GS and singleton SR-GS were performed at The Jackson Laboratory for Genomic Medicine.

High-molecular genomic DNA (gDNA) was extracted from whole blood using the PreAnalytix PAXgene Blood DNA kit (Cat. #761133) or the PacBio Nanobind PanDNA kit (PN: 103-260-000) (Table S2), according to the manufacturer's instructions. DNA concentration was measured by Qubit fluorometer with the Qubit 1X dsDNA HS Assay Kit (Thermo Fisher Scientific, Q33230). DNA integrity and fragment size distribution were assessed using the Agilent Femto Pulse system with the gDNA 165 kb Analysis Kit (Agilent FP-1002-0275). Samples with a genome quality number (GQN) ≥ 7.0 at 10 kb were selected for HiFi library preparation. For Single Molecule, Real-Time (SMRT) sequencing on the Pacific Biosciences platform, high–molecular weight genomic DNA (gDNA) was extracted from whole blood using the PreAnalytiX PAXgene Blood DNA Kit (Cat. #761133) or the PacBio Nanobind PanDNA Kit (PN: 103-260-000) (Table S2), according to the manufacturers' instructions.

DNA concentration was measured using a Qubit fluorometer with the Qubit 1X dsDNA HS Assay Kit (Thermo Fisher Scientific, Q33230). DNA integrity and fragment size distribution were assessed using the Agilent Femto Pulse system with the gDNA 165 kb Analysis Kit (Agilent FP-1002-0275). Samples with a genome quality number (GQN) ≥ 7.0 at 10 kb were selected for HiFi library preparation. Genomic DNA was sheared to a target fragment size of 16–18 kb using the Megaruptor 3 system (Diagenode, B06010003). HiFi SMRTbell libraries were prepared using the

SMRTbell Template Prep Kit 3.0 (PacBio, 102-182-700) and barcoded with SMRTbell Barcoded Adapters (PacBio, 102-009-200) to enable multiplexed sequencing. Libraries were size selected using the PippinHT system (Sage Science) to enrich for library fragments >10 kb. followed by purification, quantification, and size distribution assessment. Final size-selected HiFi SMRTbell libraries were sequenced on the PacBio Revio sequencing platform to generate highly accurate HiFi long reads achieving 30X genome coverage for comprehensive genomic analysis. For SR-GS, gDNA was fragmented and ligated to unique index adapters using an Illumina DNA PCR-free library preparation (Cat. 20041794). The library was assessed for quality using Qubit (Qubit HSDNA kit; ThermoFisher #Q32854), TapeStation (Agilent D1000 High Sensitivity Reagents #5067-5585), qPCR (KAPA library quantification universal kit; Roche #7960336001). High quality libraries were sequenced on an Illumina S2 flow cell using an Illumina NovaSeq 6000 instrument (150-bp paired-end reads) for 30X target mean coverage.

LR-GS analysis was performed on Revio fastq and unaligned bam output files with 5mC kinetics turned on using custom, in-house secondary and tertiary analysis pipelines with phased-assembly and read-based analysis workflows. In-house pipelines were run using custom scripts on the Google Cloud Platform. For the *de novo* assembly-based pipeline, trio-informed phased-assembly was performed using Hifiasm with Trio binning (v0.20.0), followed by variant calling with PAV (v2.3.4) and variant prioritization by Human Phenotype Ontology (HPO) terms using SvAnna (v1.0.5)[4,5]. For PAV, calls were made from the phased assembly against the no-alternative (no-ALT) hg38 reference genome assembly by Human Genome Structural Variation (HGSV) (ftp://ftp.1000genomes.ebi.ac.uk/vol1/ftp/data_collections/HGSVC2/technical/reference/20200513_hg38_NoALT/). For the alignment-based workflow, pbmm2 (v1.13.1) was used to align reads to the no-ALT hg38 reference. For 2990P and 2990M, alignment to the hs1 (T2T CHM13-v2.0) reference was also performed. Read-based phasing was then performed using DeepVariant (v1.6.1) and WhatsHap (v2.3)[6,7]. Small variant calling was performed with Deepvariant (v1.6.1)[6]. Structural variants were called with PacBio pbsv (v2.9.0) and Sniffles2 (v2.3.3) [8]. Quality control

analysis was performed using Nanoplot (v1.42.0) and Samtools (v1.19). CNVs were called with HiFiCNV (v1.0.0). Repeat expansions in the following 30 clinically relevant loci were called using TRGT (v1.0.0): *AFF2, AR, ATN1, ATXN1, ATXN10, ATXN2, ATXN3, ATXN7, ATXN8OS, C9ORF72, CACNA1A, CNBP, CSTB, DIP2B, DMPK, FMR1, FXN, GIPC1, GLS, HTT, JPH3, NOP56, PABPN1, PHOX2B, PPP2R2B, RFC1, STARD7, TBP, TCF4,* and *FGF14.* Variants in the following 16 clinically relevant high identity paralogs were called with Paraphase (v3.1.1): *SMN1-2, CYP21A2, TNXB, C4A/C4B, PMS2, STRC, IKBKG, NCF1, NEB, F8, CFC1, OPN1LW/OPN1MW, HBA1-2, GBA, CYP11B1-2,* and *CFH/CFHR1-4.* Population filtering was performed with SVAFotate (v0.2.0) [9] against the following LR- and SR-GS population databases, using a reciprocal overlap of 80%: Human Genome Structural Variation Consortium (HGSVC) versions 2-3[10,11], Consortium of Long Read Sequencing (CoLoRS) (colorsdb.org), Genome Answers for Kids (GA4K)[12], Trans-Omics for Precision Medicine (TOPMed),[13] Genome Aggregation Database (gnomAD)[14], 1000 Genomes[14], and the National Human Genome Research Institute Centers for Common Disease Genomics (NHGRI-CCDG) program[15]. For more information on the LR-GS pipeline, please see https://github.com/TheJacksonLaboratory/jax-apml-lrs.

Variant prioritization by HPO terms was performed using SvAnna (v1.0.4)[4,5]. Tertiary analysis was performed via ingestion of DeepVariant, pbsv, and PAV VCFs into Illumina Emedgene Software (v36.7.0). HPO terms for the proband were added and each family was analyzed as a trio or quad for segregation analysis. All 'candidate' and 'most likely' candidate variants annotated by Emedgene's explainable AI underwent variant interpretation. In addition, all variants present in several user-defined filter presets, including variants not flagged by Emedgene, were reviewed. Lastly, variant interpretation was performed on SvAnna outputs using a pathogenicity of structural variation (psv) score of >= 2 for the following tools: Sniffles2, pbsv, and PAV. Small variant (SNVs and indels) concordance analysis was performed using vcfeval within RTG Tools v3.12.1 (https://github.com/RealTimeGenomics/rtg-tools).

To benchmark the performance of the LR-GS pipeline, GIAB sample HG002 (NA24385) was sequenced to a mean coverage of 29.9X and analyzed (Table S2). Concordance of small variants (SNVs, indels) and SVs was evaluated for assembly- (PAV) and read-based (Sniffles2, pbsv, DeepVariant) variant callers. Small variant (SNVs and indels) concordance analysis was performed using vcfeval within RTG Tools v3.12.1 (https://github.com/RealTimeGenomics/rtg-tools) against the Genome-In-A-Bottle (GIAB) HG002 benchmark for small variants (GRCh38 v4.2.1)[16]. SV concordance was performed using Truvari v4.0.0 against the HGSVC3 callset for SVs[11] omitting low-confidence regions in GRCh38[10]. This approach was taken to avoid liftover operations from GRCh37 that could skew results for SVs (Figure S1). In addition, HG002 SV calls were also compared to the GIAB challenging medically relevant gene (CMRG) regions benchmark[17].

For SR-GS, output sequencing data were converted from BCL to FASTQ format using BCLConvert v4.2.7 within Illumina Connected Analytics. Alignment to the human reference genome GRCh38, variant calling, and annotation were performed using Illumina DRAGEN Germline Genome pipeline v10 within Emedgene (v36.7.0). Phenotype prioritization for both LR-GS and SR-GS pipelines was performed using the HPO terms provided in Table S1. Variants were interpreted using ACMG guidelines [18]. All variants were visually inspected in Integrative Genomics Viewer (IGV) v2.16.0.

RNA sequencing and analysis

PBMCs were isolated from whole blood samples collected from 8784 and 9548 family trios. Total RNA was extracted from ~9 million PBMCs per sample using Qiagen All prep RNA kit (#80404) according to manufacturer's instructions. Samples were ribo-depleted followed by 151 bp paired-end sequencing on the Illumina NovaSeq X Plus, 10B flow cell, ~30 million reads per sample. ERCC spike-ins (Invitrogen #4456740) were added as sequencing controls with starting concentrations according to the manufacturer's instructions (100 ng input with 1:500 dilution of

ERCC spiked in). Library preparation and sequencing were performed by the Genome Technologies Laboratory at The Jackson Laboratory for Genomic Medicine.

FASTQ preprocessing was performed using fastp (v0.23.2) (https://github.com/OpenGene/fastp). Read quality was assessed by FASTQC[19] (v0.11.9) (http://www.bioinformatics.babraham.ac.uk/projects/fastqc) and visualized with MultiQC[20] (v1.25.2) (https://github.com/MultiQC/MultiQC) (Figure S3). FASTQ reads were mapped to GRCh38.p13 reference genome with GTF annotation files as well as to the ERCC spike-in FASTA and GTF annotation files using STAR alignment (https://github.com/alexdobin/STAR). Gene and isoform expression counts were obtained using RSEM[21] (https://deweylab.github.io/RSEM). Principal component analysis (PCA) was performed on raw gene count data using pcaExplorer[22] package in R (v4.5.0). Based on PCA results, ERCC spike-in counts were used for normalization in differential expression analysis.

Given the lack of biological replicates for this analysis, differential expression analysis was performed using two computational approaches: DESeq2[23] (v1.49.4) and NOISeq[24] (v2.53.0). DESeq2 uses parametric negative binomial distribution modeling and requires biological replicates for accurate dispersion estimation with empirical Bayes shrinking. Since no biological replicates were present, we focused on hits with a $\log_2$ fold change (FC) value of ≥ |1| to explore expression trends. Conversely, NOISeq is a nonparametric tool that utilizes empirical noise modeling and does not require biological replicates when using the NOISeq-sim function, which can simulate noise for no-replicate datasets. Probabilistic score (p) of > 0.9 and $\log_2$FC > |1| were considered for analysis. To optimize information obtained from no-replicate data, and potentially reduce age or outlier effects, the following four comparisons were performed using DESeq2 and NOISeq for each trio: (1) proband (8784P or 9548P) vs parents, (2) proband vs unaffected adults (8784M/F, 9548M/F), (3) proband vs all other samples (8784M/F, 9548M/F, and 8784P or 9548P), (4) proband vs all other samples excluding 8784F (8784M, 9548M/F, and 8784P or 9548P) due to potential outlier effects as suggested by PCA and QC data. DESeq2 hits with $\log_2$FC ≥ |1| (and

NOISeq hits with $log_2FC \geq |1|$ plus $p > 0.9$) were merged across all four comparisons and approach per proband establishing a candidate list, and any hits that were also present in the other proband's candidate list were removed. Pathway and process enrichment analysis were performed on these candidate gene lists using MetaScape (https://metascape.org/)[25]. Plots were created using ggplot2 and pheatmap packages in R.

Computational tools and online resources

Combined Annotation Dependent Depletion (CADD) Phred scores were obtained using CADD v1.7 against GRCh38 (https://cadd.gs.washington.edu). MetaDome analysis was performed on primary transcripts for research findings using the online tool (stuart.radboudumc.nl/metadome).[26] The following additional *in silico* and deep learning tools were used for variant effect predictions against GRCh38 (Table S4): AlphaMissense (https://alphamissense.hegelab.org/)[27], PolyPhen-2 (http://genetics.bwh.harvard.edu/pph2/)[28], SpliceAI (https://spliceailookup.broadinstitute.org/)[29], PrimateAI (https://github.com/Illumina/PrimateAI)[30], and DDMut (https://biosig.lab.uq.edu.au/ddmut/)[31]. Visualization of the *H. sapiens* AlphaFold[32] model AF-O14925 F1 and *S. cerevisiae* TIM23 Complex cryogenic electron microscopy (cryo-EM) structure was performed using the PDB:8E1M model[33] in PyMOL (v2.5.2). Disease information was obtained from the Online Mendelian Inheritance in Man (OMIM, http://www.omim.org). Protein information was obtained from Uniprot.org.

References

1. Consortium, G. (2013). The Genotype-Tissue Expression (GTEx) project. Nat Genet *45*, 580-585. 10.1038/ng.2653.
2. Patowary, A., Zhang, P., Jops, C., Vuong, C.K., Ge, X., Hou, K., Kim, M., Gong, N., Margolis, M., Vo, D., et al. (2023). Developmental isoform diversity in the human neocortex informs neuropsychiatric risk mechanisms. bioRxiv. 10.1101/2023.03.25.534016.
3. Reese, F., Williams, B., Balderrama-Gutierrez, G., Wyman, D., Çelik, M.H., Rebboah, E., Rezaie, N., Trout, D., Razavi-Mohseni, M., Jiang, Y., et al. (2023). The ENCODE4 long-read RNA-seq collection reveals distinct classes of transcript structure diversity. bioRxiv. 10.1101/2023.05.15.540865.
4. Robinson, P.N., Kohler, S., Oellrich, A., Sanger Mouse Genetics, P., Wang, K., Mungall, C.J., Lewis, S.E., Washington, N., Bauer, S., Seelow, D., et al. (2014). Improved exome prioritization of disease genes through cross-species phenotype comparison. Genome Res *24*, 340-348. 10.1101/gr.160325.113.
5. Danis, D., Jacobsen, J.O.B., Balachandran, P., Zhu, Q., Yilmaz, F., Reese, J., Haimel, M., Lyon, G.J., Helbig, I., Mungall, C.J., et al. (2022). SvAnna: efficient and accurate pathogenicity prediction of coding and regulatory structural variants in long-read genome sequencing. Genome Med *14*, 44. 10.1186/s13073-022-01046-6.
6. Poplin, R., Chang, P.C., Alexander, D., Schwartz, S., Colthurst, T., Ku, A., Newburger, D., Dijamco, J., Nguyen, N., Afshar, P.T., et al. (2018). A universal SNP and small-indel variant caller using deep neural networks. Nat Biotechnol *36*, 983-987. 10.1038/nbt.4235.
7. Martin, M., Ebert, P., and Marschall, T. (2023). Read-Based Phasing and Analysis of Phased Variants with WhatsHap. Methods Mol Biol *2590*, 127-138. 10.1007/978-1-0716-2819-5_8.
8. Smolka, M., Paulin, L.F., Grochowski, C.M., Horner, D.W., Mahmoud, M., Behera, S., Kalef-Ezra, E., Gandhi, M., Hong, K., Pehlivan, D., et al. (2024). Detection of mosaic and population-level structural variants with Sniffles2. Nat Biotechnol. 10.1038/s41587-023-02024-y.
9. Nicholas, T.J., Cormier, M.J., and Quinlan, A.R. (2022). Annotation of structural variants with reported allele frequencies and related metrics from multiple datasets using SVAFotate. BMC Bioinformatics *23*, 490. 10.1186/s12859-022-05008-y.
10. Ebert, P., Audano, P.A., Zhu, Q., Rodriguez-Martin, B., Porubsky, D., Bonder, M.J., Sulovari, A., Ebler, J., Zhou, W., Serra Mari, R., et al. (2021). Haplotype-resolved diverse human genomes and integrated analysis of structural variation. Science *372*. 10.1126/science.abf7117.
11. Logsdon, G.A., Ebert, P., Audano, P.A., Loftus, M., Porubsky, D., Ebler, J., Yilmaz, F., Hallast, P., Prodanov, T., Yoo, D., et al. (2025). Complex genetic variation in nearly complete human genomes. Nature *644*, 430-441. 10.1038/s41586-025-09140-6.
12. Cohen, A.S.A., Farrow, E.G., Abdelmoity, A.T., Alaimo, J.T., Amudhavalli, S.M., Anderson, J.T., Bansal, L., Bartik, L., Baybayan, P., Belden, B., et al. (2022). Genomic answers for children: Dynamic analyses of >1000 pediatric rare disease genomes. Genet Med *24*, 1336-1348. 10.1016/j.gim.2022.02.007.
13. Taliun, D., Harris, D.N., Kessler, M.D., Carlson, J., Szpiech, Z.A., Torres, R., Taliun, S.A.G., Corvelo, A., Gogarten, S.M., Kang, H.M., et al. (2021). Sequencing of 53,831 diverse genomes from the NHLBI TOPMed Program. Nature *590*, 290-299. 10.1038/s41586-021-03205-y.
14. Koenig, Z., Yohannes, M.T., Nkambule, L.L., Zhao, X., Goodrich, J.K., Kim, H.A., Wilson, M.W., Tiao, G., Hao, S.P., Sahakian, N., et al. (2024). A harmonized public resource of

deeply sequenced diverse human genomes. Genome Res *34*, 796-809. 10.1101/gr.278378.123.
15. Abel, H.J., Larson, D.E., Regier, A.A., Chiang, C., Das, I., Kanchi, K.L., Layer, R.M., Neale, B.M., Salerno, W.J., Reeves, C., et al. (2020). Mapping and characterization of structural variation in 17,795 human genomes. Nature *583*, 83-89. 10.1038/s41586-020-2371-0.
16. Wagner, J., Olson, N.D., Harris, L., Khan, Z., Farek, J., Mahmoud, M., Stankovic, A., Kovacevic, V., Yoo, B., Miller, N., et al. (2022). Benchmarking challenging small variants with linked and long reads. Cell Genom *2*. 10.1016/j.xgen.2022.100128.
17. Wagner, J., Olson, N.D., Harris, L., McDaniel, J., Cheng, H., Fungtammasan, A., Hwang, Y.C., Gupta, R., Wenger, A.M., Rowell, W.J., et al. (2022). Curated variation benchmarks for challenging medically relevant autosomal genes. Nat Biotechnol *40*, 672-680. 10.1038/s41587-021-01158-1.
18. Richards, S., Aziz, N., Bale, S., Bick, D., Das, S., Gastier-Foster, J., Grody, W.W., Hegde, M., Lyon, E., Spector, E., et al. (2015). Standards and guidelines for the interpretation of sequence variants: a joint consensus recommendation of the American College of Medical Genetics and Genomics and the Association for Molecular Pathology. Genet Med *17*, 405-424. 10.1038/gim.2015.30.
19. Wingett, S.W., and Andrews, S. (2018). FastQ Screen: A tool for multi-genome mapping and quality control. F1000Res *7*, 1338. 10.12688/f1000research.15931.2.
20. Ewels, P., Magnusson, M., Lundin, S., and Käller, M. (2016). MultiQC: summarize analysis results for multiple tools and samples in a single report. Bioinformatics *32*, 3047-3048. 10.1093/bioinformatics/btw354.
21. Li, B., and Dewey, C.N. (2011). RSEM: accurate transcript quantification from RNA-Seq data with or without a reference genome. BMC Bioinformatics *12*, 323. 10.1186/1471-2105-12-323.
22. Marini, F., and Binder, H. (2019). pcaExplorer: an R/Bioconductor package for interacting with RNA-seq principal components. BMC Bioinformatics *20*, 331. 10.1186/s12859-019-2879-1.
23. Love, M.I., Huber, W., and Anders, S. (2014). Moderated estimation of fold change and dispersion for RNA-seq data with DESeq2. Genome Biol *15*, 550. 10.1186/s13059-014-0550-8.
24. Tarazona, S., García-Alcalde, F., Dopazo, J., Ferrer, A., and Conesa, A. (2011). Differential expression in RNA-seq: a matter of depth. Genome Res *21*, 2213-2223. 10.1101/gr.124321.111.
25. Zhou, Y., Zhou, B., Pache, L., Chang, M., Khodabakhshi, A.H., Tanaseichuk, O., Benner, C., and Chanda, S.K. (2019). Metascape provides a biologist-oriented resource for the analysis of systems-level datasets. Nat Commun *10*, 1523. 10.1038/s41467-019-09234-6.
26. Wiel, L., Baakman, C., Gilissen, D., Veltman, J.A., Vriend, G., and Gilissen, C. (2019). MetaDome: Pathogenicity analysis of genetic variants through aggregation of homologous human protein domains. Hum Mutat *40*, 1030-1038. 10.1002/humu.23798.
27. Minton, K. (2023). Predicting variant pathogenicity with AlphaMissense. Nat Rev Genet *24*, 804. 10.1038/s41576-023-00668-9.
28. Adzhubei, I., Jordan, D.M., and Sunyaev, S.R. (2013). Predicting functional effect of human missense mutations using PolyPhen-2. Curr Protoc Hum Genet *Chapter 7*, Unit7.20. 10.1002/0471142905.hg0720s76.
29. Jaganathan, K., Kyriazopoulou Panagiotopoulou, S., McRae, J.F., Darbandi, S.F., Knowles, D., Li, Y.I., Kosmicki, J.A., Arbelaez, J., Cui, W., Schwartz, G.B., et al. (2019). Predicting Splicing from Primary Sequence with Deep Learning. Cell *176*, 535-548.e524. 10.1016/j.cell.2018.12.015.

30. Sundaram, L., Gao, H., Padigepati, S.R., McRae, J.F., Li, Y., Kosmicki, J.A., Fritzilas, N., Hakenberg, J., Dutta, A., Shon, J., et al. (2018). Predicting the clinical impact of human mutation with deep neural networks. Nat Genet *50*, 1161-1170. 10.1038/s41588-018-0167-z.
31. Zhou, Y., Pan, Q., Pires, D.E.V., Rodrigues, C.H.M., and Ascher, D.B. (2023). DDMut: predicting effects of mutations on protein stability using deep learning. Nucleic Acids Res *51*, W122-W128. 10.1093/nar/gkad472.
32. Jumper, J., Evans, R., Pritzel, A., Green, T., Figurnov, M., Ronneberger, O., Tunyasuvunakool, K., Bates, R., Žídek, A., Potapenko, A., et al. (2021). Highly accurate protein structure prediction with AlphaFold. Nature *596*, 583-589. 10.1038/s41586-021-03819-2.
33. Sim, S.I., Chen, Y., Lynch, D.L., Gumbart, J.C., and Park, E. (2023). Structural basis of mitochondrial protein import by the TIM23 complex. Nature *621*, 620-626. 10.1038/s41586-023-06239-6.
